# ALKBH5/YTHDF2‐mediated m6A modification of circAFF2 enhances radiosensitivity of colorectal cancer by inhibiting Cullin neddylation

**DOI:** 10.1002/ctm2.1318

**Published:** 2023-06-28

**Authors:** Yingjie Shao, Zhenhua Liu, Xing Song, Rui Sun, You Zhou, Dachuan Zhang, Huihui Sun, Junchao Huang, Chenxi Wu, Wendong Gu, Xiao Zheng, Jingting Jiang

**Affiliations:** ^1^ Department of Radiation Oncology The Third Affiliated Hospital of Soochow University Changzhou China; ^2^ Department of Radiotherapy The Yancheng Clinical College of Xuzhou Medical University The First people's Hospital of Yancheng Yancheng China; ^3^ Department of Tumor Biological Treatment The Third Affiliated Hospital of Soochow University Changzhou China; ^4^ Jiangsu Engineering Research Center for Tumor Immunotherapy Changzhou China; ^5^ Institute of Cell Therapy Soochow University Changzhou China; ^6^ Department of Pathology The Third Affiliated Hospital of Soochow University Changzhou China

**Keywords:** ALKBH5, circAFF2, m6A, radiosensitivity, YTHDF2

## Abstract

**Background:**

Circular RNA (circRNA) and N6‐methyladenosine (m6A) play a critical role in tumour occurrence and development, including colorectal cancer (CRC). However, little is known about the interaction between circRNA and m6A in the radiosensitivity of CRC. Here, we investigated the role of a novel m6A‐regulated circRNA in CRC.

**Methods:**

Differentially expressed circRNAs from radiosensitive and radioresistant CRC tissues were screened. Modifications of the selected circRNAs were examined by methylated RNA immunoprecipitation assay. Finally, the selected circRNAs were subjected to radiosensitivity assay.

**Results:**

We identified that circAFF2 is closely related to both radiosensitivity and m6A in CRC. CircAFF2 was highly expressed in patients with radiosensitive rectal cancer, and patients with high expression of circAFF2 had a better prognosis. In addition, circAFF2 can enhance the radiosensitivity of CRC cells both in vitro and in vivo. The regulation of circAFF2 involves ALKBH5‐mediated demethylation, followed by its recognition and degradation via YTHDF2. Rescue experiments revealed that circAFF2 could reverse the radiosensitivity induced by ALKBH5 or YTHDF2. Mechanistically, circAFF2 binds with CAND1, promotes the binding of CAND1 to Cullin1 and inhibits its neddylation, subsequently impacting the radiosensitivity of CRC.

**Conclusion:**

We identified and characterised circAFF2 as a novel m6A‐modified circRNA and validated the ALKBH5/YTHDF2/circAFF2/Cullin‐NEDD8 axis as a potential radiotherapy target for CRC.

## Graphical abstract

The expression of circAFF2 is regulated by ALKBH5‐mediated demethylation, and YTHDF2 mainly mediates its degradation in an m6A‐dependent manner. CircAFF2 enhanced the radiosensitivity of CRC cells by binding to CAND1 and promoting the interaction of CAND1 with Cullin1, thereby inhibiting neddylation modification in CRC cells.

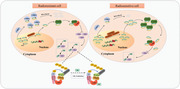

## 1 INTRODUCTION

Colorectal cancer (CRC) is a prevalent malignancy worldwide, ranking third in morbidity and second in mortality.^1^ The rectum is the most common site of colorectal tumours. The standard treatment for locally advanced rectal cancer now involves preoperative concurrent chemoradiotherapy followed by radical surgery.^2^, ^3^, ^4^ Nevertheless, after neoadjuvant therapy, 50%−60% of patients with locally advanced rectal cancer achieve partial response, while 15%−20% achieve pathological complete response (pCR).^5^ However, some patients still cannot benefit from neoadjuvant chemoradiotherapy. Particularly, preoperative radiotherapy can lead to disease progression in patients with limited response to radiotherapy, thereby delaying surgical treatment. Therefore, it is imperative to identify novel biomarkers to screen patients with rectal cancer sensitive to preoperative radiotherapy and chemotherapy, as well as to uncover radiosensitisers to enhance the curative effect.

Circular RNA (circRNA) has attracted increasing attention with the emergence of RNA next‐generation sequencing, circRNA.^6^ Unlike linear RNAs, circRNAs are a class of endogenous non‐coding RNA molecules that lack a 5′ end cap and 3′ end poly (A) tail and form a closed ring structure with covalent bonds.^7^ Substantial evidence shows that circRNAs play a significant role in various biological processes, including functioning as serving as sponges for proteins or miRNAs, encoding polypeptides, or forming stable complexes with RNA and proteins to govern downstream biological processes.^7^, ^8^

The most prevalent RNA modification in eukaryotes is N6‐methyladenosine (m6A),^9^ which is usually located in the coding sequence, the 3′ untranslated region and the stop codon.^10^ Methyltransferase enzymes (METTL3, METTL14, RMB15, WTAP, VIRMA), demethylase enzymes (FTO, ALKBH5) and methylated reading proteins (YTHDF1–3, YTHDC1–2, IGF2BPs) dynamically regulate m6A modification, which plays a critical role in RNA transcription, shearing, output, translation, localisation and stability.^11^ ALKBH5, an RNA demethylase, is a key factor that maintains the dynamic and reversible m6A methylation modification level.^12^ It directly catalyses the RNA adenosine to remove the methyl group, thereby regulating the various RNA functions.^12^ Abnormal expression of ALKBH5 in CRC has been shown to be associated with its proliferation and invasion ability.^13^, ^14^, ^15^, ^16^ YTHDF2, the first m6A reading protein to be identified and fully studied, decreases mRNA and circRNA stability through various pathways.^17^, ^18^, ^19^, ^20^

Neddylation is a type of post‐translational modification that is analogous to ubiquitination, where a protein called NEDD8, which is similar to ubiquitin, is attached to a specific target protein.^21^ This modification plays a critical role in tumour development and radiosensitivity.^22^, ^23^, ^24^ As an essential component of the polysubunit Cullin‐ring ligases (CRLs), the Cullin family is the most common substrate for neddylation.^25^ CAND1 selectively associates with Cullin1 and inhibits cellular neddylation modification.^26^, ^27^, ^28^

Recent research suggests that m6A post‐translational modification regulates critical biological functions of circRNAs,^29^ providing a new perspective of epigenetic regulation in tumours and a novel target to overcome tumour radioresistance.^11^ However, the function and mechanism of m6A‐modified circRNAs in rectal cancer radiosensitivity remain unclear. This study identified differentially expressed circRNAs from radiosensitive and radioresistant rectal cancer tissues and subjected them to methylated RNA immunoprecipitation (Me‐RIP) and radiosensitivity assays to explore the association between m6A‐modified circRNAs and radiosensitivity in rectal cancer. Subsequently, we demonstrated that m6A‐regulated circAFF2 inhibits neddylation by promoting the binding of CAND1 to Cullin1, thereby increasing the radiosensitivity of rectal cancer.

## 2 MATERIALS AND METHODS

### 2.1 Human tissue samples

The human tissue samples used in the current study were collected from the pre‐treatment colonoscopy specimens of patients with neoadjuvant chemoradiotherapy for rectal cancer in the Third Affiliated Hospital of Soochow University. Samples were stored in liquid nitrogen following collection. The rectal cancer specimens were collected from patients without any anti‐tumour therapies, including immunotherapy, targeted therapy, biological therapy and surgery. Tumour regression grading (TRG) was used for the pathological analysis of tumour specimens after neoadjuvant chemoradiotherapy to evaluate the therapeutic effect.^30^, ^31^ The TRG score was based on the National Comprehensive Cancer Network criteria.^31^ TRG grade 0 represents absence of residual cancer cells, that is, pCR. TRG grade 1 represents only a single cancer cell or cancer cell cluster and TRG grade 2 is characterised by fibrosis overgrowing cancer cells. TRG grade 3 indicates almost no fibrosis, and a large amount of residual cancer can be seen. Two independent pathologists generated the pathological diagnosis report of the specimen. We informed patients during specimen collection and pre‐treatment interviews, and informed consent was signed. The Ethics Committee of the Third Affiliated Hospital of Soochow University approved this study in accordance with the Declaration of Helsinki (Approval No. 190 [2018]).

### 2.2 Cell culture

Cell lines used in the study, including RKO, SW480, HT‐29, LoVo, HCT‐116, SW620 and NCM460, were obtained from the American Type Culture Collection. The human colorectal cancer cell line was cultured in Dulbecco's modified Eagle's medium (Gibco), supplemented with 10% foetal bovine serum (Gibco), 100 units/mL penicillin and 100 μg/mL streptomycin (Gibco). The cells were cultured in 5% CO_2_ at 37°C. The medium was changed every 3 days. When the cells reached 80%−90% confluency, they were passaged with .05% Trypsin + .53 mM Ethylene Diamine Tetraacetic Acid (EDTA) (Gibco).

Plasmids overexpressing circRNAs, short hairpin RNA (shRNA) of circRNAs, and negative control plasmids were purchased from Generbiol (Shanghai, China). Sequences of siRNAs and shRNAs used in the plasmid construction are shown in Table S1.

Details about in situ hybridisation (ISH), immunohistochemistry (IHC), RNA preparation, real‐time Quantitative Polymerase Chain Reaction (qPCR), immunoblotting, colony formation assay, apoptosis detection, γ‐H2AX quantification, RNase R resistance assay, nuclear‐cytoplasmic fractionation, fluorescence in situ hybridisation (FISH), RNA m6A methylation quantification, dot blot, RNA immunoprecipitation (RIP), Me‐RIP‐qPCR, luciferase reporter assay, co‐immunoprecipitation (co‐IP) assay, RNA pull‐down analysis and mass spectrometry and animal experiments are described in Supporting Information. The primers are shown in Table S2 and S3.

### 2.3 Statistical analysis

The mean ± SD of data from three independent experiments was presented, and the difference between two groups was analysed using Student's *t*‐test. The survival outcome of patients with different levels of circAFF2 (high/low groups) was evaluated by Kaplan–Meier analysis (log‐rank test). Statistically significant differences were defined as those with *p* < .05. Otherwise, there was no statistical difference. All statistical analyses were conducted using GraphPad Prism 9.0 with bilateral statistical tests.

## 3 RESULTS

### 3.1 Screening and characterising circAFF2 in CRC

**FIGURE 1:**
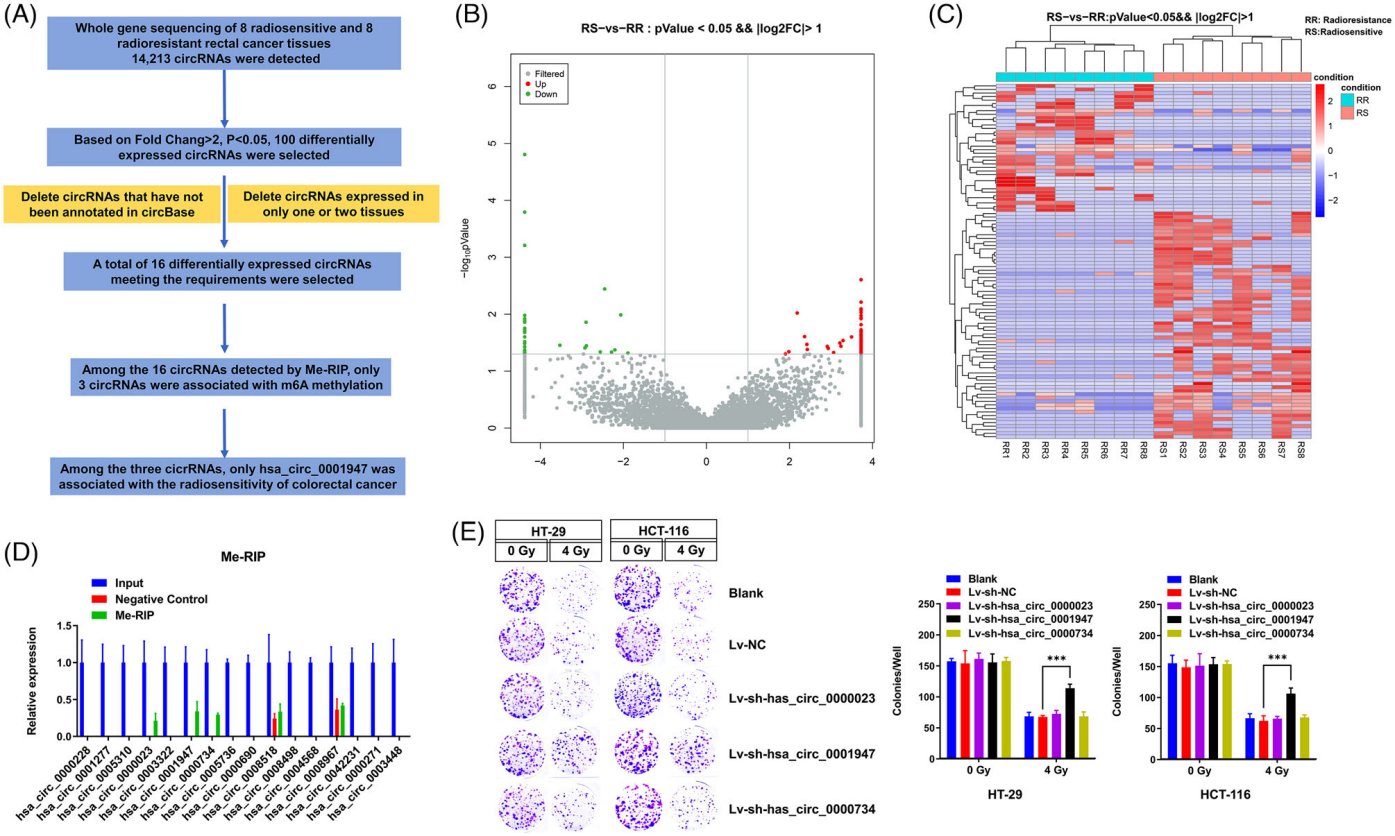
Screening m6A‐related circAFF2 in colorectal cancer (CRC). (A) The flowchart showing the selection processes of hsa_circ_0001947. (B and C) After the gene sequencing of the radiosensitive and radioresistant tissues of rectal cancer, the heat map and volcano plot (fold change >2, *p* < .05) of the differential circular RNAs (circRNAs) were analysed. (D) Methylated RNA immunoprecipitation (Me‐RIP) screened the methylation modification of candidate circRNAs. (E) The radiosensitivity of candidate circRNAs in colon cancer HT‐29 and HCT‐116 cells was detected using a colony formation assay preliminarily detected the radiosensitivity of candidate circRNAs in colon cancer HT‐29 and HCT‐116 cells.

The screening process of m6A‐modified circRNAs in rectal cancer radiosensitivity is shown in Figure 1A. We collected tissue specimens from 16 locally advanced rectal cancer cases before neoadjuvant chemoradiotherapy. The treatment plan for these patients was a combination of neoadjuvant chemoradiotherapy and surgical removal. Of these, eight cases with postoperative pathology of TRG 0 and TRG 1 were selected as the radiosensitive group, while eight cases of TRG 2 and TRG 3 were selected as the radioresistant group. Using a genome‐wide investigation, 14 213 new circRNAs with a total length of 35 039 505 nt and an average length of 2465.31 nt were detected. We identified 100 differentially expressed circRNAs between radiosensitive and radioresistance groups (fold change > 2, *p* < .05, Figure 1B,C). We further excluded circRNAs that were not annotated in circBase (http://www.circbase.org/) and those that were only expressed in one or two specimens to obtain 16 circRNAs for further study. Of these, nine were highly expressed in the radiosensitive group (hsa_circ_0000690, hsa_circ_0005736, hsa_circ_0000734, hsa_circ_0001947, hsa_circ_0003322, hsa_circ_0000023, hsa_circ_0005310, hsa_circ_0001277 and hsa_circ_0000228) and seven had low expression levels in the radiosensitive group (hsa_circ_0003448, hsa_circ_0000271, hsa_circ_0042231, hsa_circ_0008967, hsa_circ_0004568, hsa_circ_0008498 and hsa_circ_0008518). To investigate whether these circRNAs have m6A methylation modification, we performed a Me‐RIP assay in HCT‐116 CRC cells and found that hsa_circ_0000023, hsa_circ_0001947 and hsa_circ_0000734 were m6A methylated (Figure 1D). To further investigate the association of these three circRNAs with the radiosensitivity of CRC, CRC cells with downregulated hsa_circ_0000023, hsa_circ_0001947 or hsa_circ_0000734 were constructed. The expression levels of circRNAs in the transfected HCT‐116 and HT‐29 are shown in Figure S1A. Colony formation assays revealed that only hsa_circ_0001947 was associated with the radiosensitivity of CRC cells (Figure 1E). Therefore, hsa_circ_0001947 was selected to further investigate the association between circRNAs and the radiosensitivity of CRC.

**FIGURE 2:**
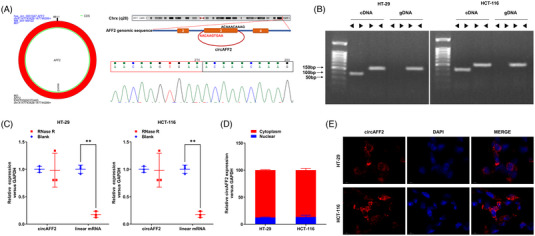
CircAFF2 was identified with circular features. (A) hsa_circ_0001947 is formed by the circularisation of exon 3 of AFF2 gene with a spliced length of 861 bp, which was verified by Sanger sequencing. (B) cDNA and genomic gDNA reverse transcribed from circAFF2 were subjected to PCR amplification in HT‐29 and HCT‐166 cells. (C) Differences between circAFF2 and linear RNA upon RNase R treatment in HT‐29 and HCT‐116 cells. (D) Nucleocytoplasmic separation assay to characterise the distribution of circAFF2 in HT‐29 and HCT‐116 cells. (E) The distribution of circAFF2 was examined by fluorescence in situ hybridisation (FISH) assay in HT‐29 and HCT‐116 cells. ^***^ *p* < .001, ^**^ *p* < .01, ^*^ *p* < .05.

The circBase database revealed that hsa_circ_0001947 is located on chromosome X (147 743 428–147 744 289) and formed by the circularisation of the single exon 3 of the *AFF2* gene with a spliced length of 861 bp (hereinafter referred to as circAFF2). The circular structure of circAFF2 was further verified by Sanger sequencing (Figure 2A). A study by Zhou et al.^19^ suggested that long single‐exon circRNAs are more susceptible to m6A methylation. Both the cDNA from the reverse transcription of circAFF2 and the directly extracted gDNA were amplified by PCR with the convergent primers. However, only cDNA from reverse transcription of circAFF2 can amplify the circRNA fragment with the divergent primer, which indicated that circAFF2 is a circular structure (Figure 2B). Furthermore, RNase R treatment of the HT‐29 and HCT‐116 cells revealed that circAFF2 was more stable than linear RNA (Figure 2C). Nucleocytoplasmic separation and FISH assays demonstrated that circAFF2 was primarily located in the cytoplasm of CRC cells (Figure 2D,E). Together, our findings suggest that circAFF2 is a circular structure and is stably expressed in the cytoplasm of CRC cells.

### 3.2 CircAFF2 increases the radiosensitivity of CRC

**FIGURE 3:**
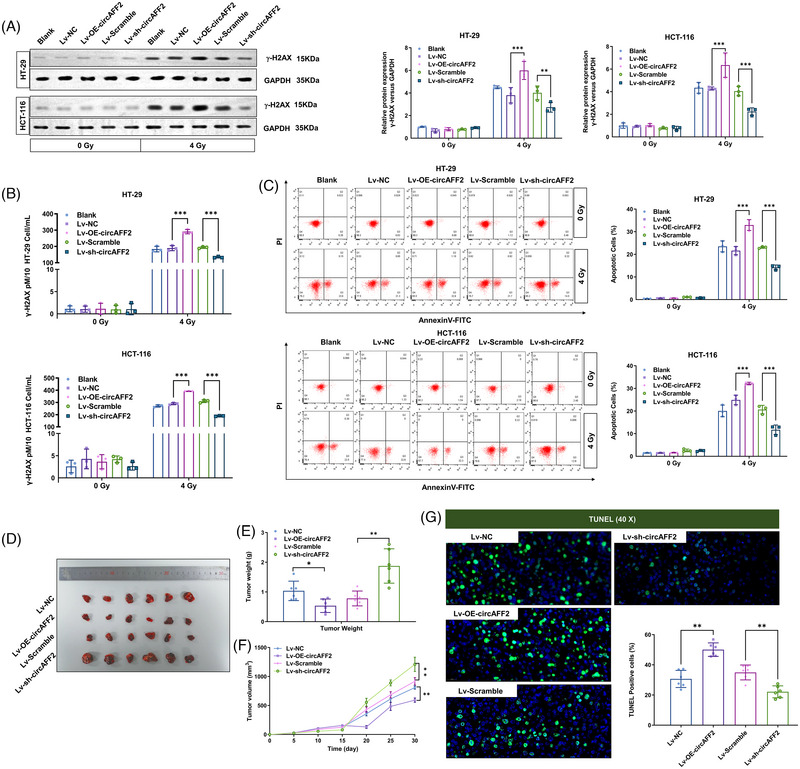
CircAFF2 increases the radiosensitivity of colorectal cancer (CRC) cells in vitro and in vivo. Western blot (A) and ELISA assay (B) detected γ‐H2AX expression in circAFF2‐overexpressed or circAFF2‐depleted HCT‐116 and HT‐29 cells after 4 Gy irradiation. (C) One day after 4 Gy irradiation, apoptosis of circAFF2‐overexpressed or circAFF2‐depleted HCT‐116 and HT‐29 cells was examined by flow cytometry. (D) Representative images of collected tumours. (E) Subcutaneous tumour weights in tumour‐bearing mice. (F) Growth curve of subcutaneous tumour in tumour‐bearing mice. (G) Representative images of TUNEL staining in mouse tumour tissue. ^***^ *p* < .001, ^**^ *p* < .01, ^*^ *p* < .05.

Our data revealed that among RKO, SW480, HT‐29, LoVo, HCT‐116, SW620 and NCM460 cell lines, HCT‐116 is the most radiosensitive, while HT‐29 is the least radiosensitive (Figure S2). Therefore, HCT‐116 and HT‐29 were used in the following in vitro experiments. We observed that the expression levels of circAFF2 in HCT‐116 were higher than in HT‐29 (Figure S1B). As demonstrated in our colony formation assay, downregulation of circAFF2 significantly increased the cell survival fraction (Figure 1E). γ‐H2AX is an indicator of cellular response to DNA damage, in cells 6 h after 4 Gy irradiation. We used western blotting to detect the expression of γ‐H2AX in HCT‐116 and HT‐29 cells at different time points (0, 1, 2, 4, 6, 8 and 20 h) after being exposed to 4 Gy radiation. The expression of γ‐H2AX peaked at 1 h and then rapidly decreased afterwards. We found that the expression of γ‐H2AX in HCT‐116 and HT‐29 cell lines overexpressing circAFF2 was significantly different from that in the control group at 4−20 h after irradiation (Figure S3). This demonstrates that it is feasible to detect the expression of γ‐H2AX between 4 and 20 h after radiation therapy. Combined with the work and rest arrangement of experimental personnel, as well as the convenience and rationality of the operation process from irradiation room to laboratory, we chose to detect γ‐H2AX at 6 h post‐irradiation. We found that γ‐H2AX levels were dramatically upregulated in cells overexpressing circAFF2, while γ‐H2AX expression was significantly decreased in circAFF2‐depleted cells (Figure 3A,B). Consistently, overexpression of circAFF2 in CRC cells significantly increased cell apoptosis, while cell apoptosis was attenuated in circAFF2‐depleted CRC cells (Figure 3C), suggesting that circAFF2 could increase the radiosensitivity of CRC cells.

Next, we investigated the effect of circAFF2 on the radiosensitivity of CRC in vivo. To achieve this, we utilised a subcutaneous tumour grafting mouse model by injecting CRC HCT‐116 cells transfected with Lv‐NC, Lv‐OE‐circAFF2, Lv‐Scramble or Lv‐sh‐circAFF2. Ten days after tumour grafting (tumour volume reaches 200 mm^3^), mice were exposed to 10 Gy radiation (single dose). Tumour sizes were recorded every 5 days following radiation. Mice were anaesthetised and tissues were collected 30 days after irradiation. Representative images of collected tumours are presented in Figure 3D. The circAFF2‐overexpressed tumour weight was significantly reduced, while the weight of circAFF2‐depleted tumours was significantly increased in comparison to control tumours (Figure 3E). Additionally, the growth rate of circAFF2‐overexpressed tumours was slower, and the tumour volume was smaller than that of the control tumours. On the other hand, circAFF2‐depleted tumours exhibited a faster growth rate and larger tumour volume (Figure 3F). Moreover, we performed TUNEL assay to evaluate the cell apoptosis and observed that apoptosis was significantly increased in circAFF2‐overexpressed tumours and reduced in circAFF2‐depleted tumours following radiation compared to the control tumours (Figure 3G). Collectively, our data suggest that circAFF2 can increase the radiosensitivity of CRC both in vitro and in vivo.

### 3.3 CircAFF2 expression is regulated by ALKBH5/YTHDF2‐mediated m6A methylation

**FIGURE 4:**
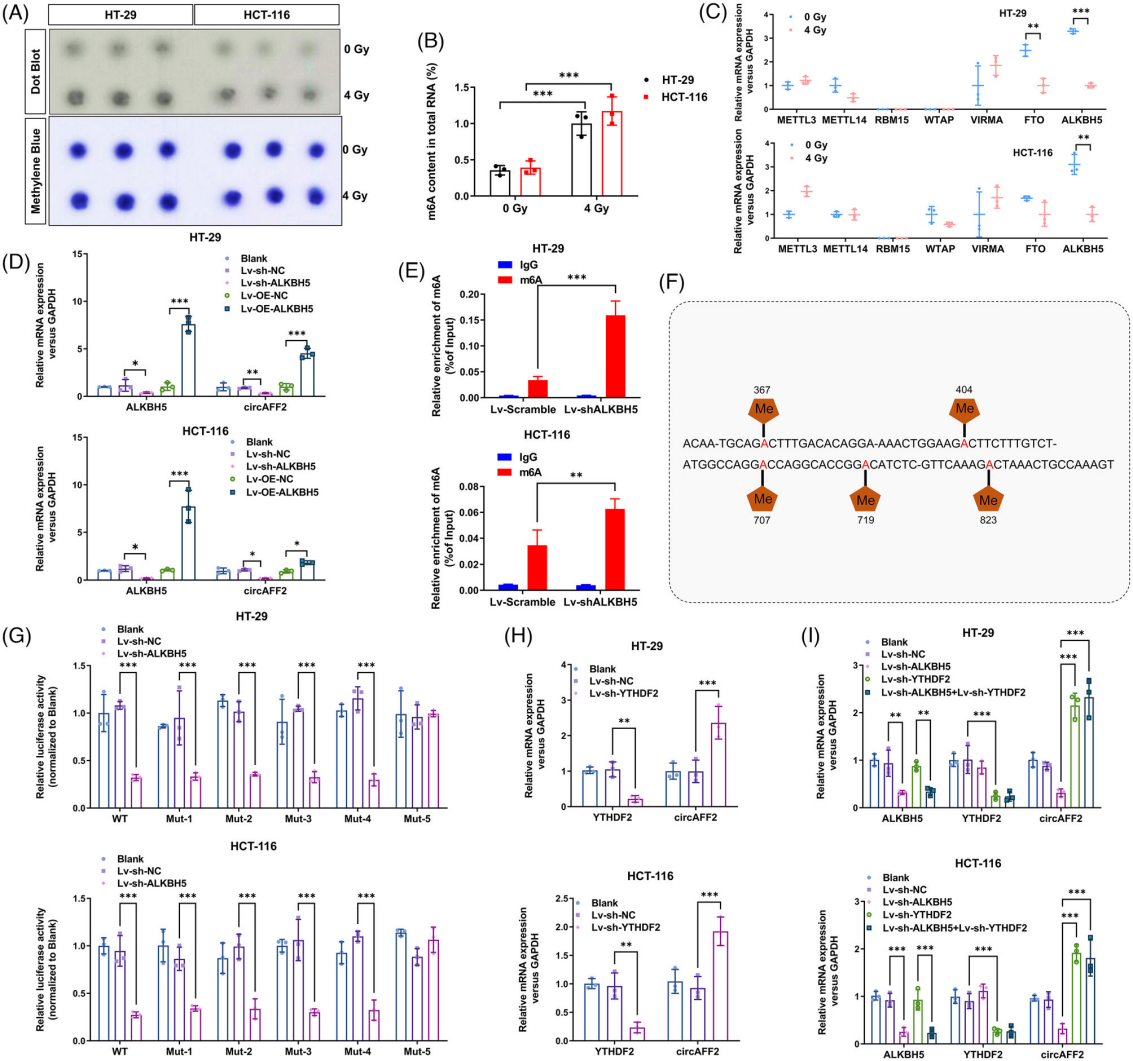
CircAFF2 expression is regulated by ALKBH5/YTHDF2‐mediated m6A methylation. Dot blot (A) and m6A RNA methylation assay (B) showing m6A levels in HT‐29 and HCT‐116 cells before and after irradiation. (C) mRNA levels of methylases and demethylases in HT‐29 and HCT‐116 cells following 4 Gy irradiation. (D) qRT‐PCR to detect circAFF2 expression in ALKBH5‐overexpressed or ALKBH5‐depleted HT‐29 and HCT‐116 cells. (E) Methylated RNA immunoprecipitation (Me‐RIP)‐qPCR showed that depletion of ALKBH5 increases the m6A modification of circAFF2. (F) The constructed luciferase reporter plasmids by inserting partial wild‐type circAFF2 sequences or mutated m6A sites (M1–M5) circAFF2 sequences. (G) The luciferase reporters with wild‐type or mutant plasmids were transfected into HT‐29 and HCT‐116 cells (with the perturbation of ALKBH5), followed by the measurement of luciferase activity. (H) qRT‐PCR to detect circAFF2 expression in HT‐29 and HCT‐116 transfected downregulated YTHDF2. (I) qRT‐PCR to detect circAFF2 expression in HT‐29 and HCT‐116 transfected downregulated ALKBH5 or/both downregulated YTHDF2. ^***^ *p* < .001, ^**^ *p* < .01, ^*^ *p* < .05.

We then assessed m6A levels in radiation‐exposed CRC cells using Dot blot and m6A methylation assay. The m6A levels of HT‐29 and HCT‐116 cells were significantly increased after radiation, suggesting that m6A methylation was induced upon radiation (Figure 3A,B). The proteins involved in the regulation of m6A methylation mainly include methyltransferases (METTL3, METTL14, RMB15, WTAP, VIRMA), demethylases (FTO, ALKBH5) and RNA‐binding proteins (YTHDF1–3, YTHDC1–2, IGF2BPs). The level of ALKBH5, an m6A‐related demethylase, was reduced in both HCT‐116 and HT‐29 after radiation, while no differences in METTL3, METTL14, RBM15, WTAP and VIRMA were observed (Figure 4C). FTO was statistically different in HT‐29, but not in HCT‐116 (Figure 4C). Therefore, ALKBH5 could be related to radiotherapy‐induced m6A methylation in CRC cells. We then constructed a lentiviral vector for ALKBH5 overexpression and downregulation. Transfection efficiency is shown in Figure S1C. As expected, we observed a significant decrease in the m6A level in ALKBH5‐overexpressed CRC cells and an increase in the m6A level in ALKBH5‐downregulated CRC cells (Figure S1D,E). Interestingly, colony formation assay showed that the survival fraction of cells after radiation decreased significantly in ALKBH5‐overexpressed CRC HCT‐116 and HT‐29 cells. At the same time, the survival fraction of cells after radiation increased in ALKBH5‐downregulated CRC HCT‐116 and HT‐29 cells (Figure S4A). Moreover, we observed a significant increase in the expression of γ‐H2AX in the ALKBH5‐overexpressing cells, while the expression of γ‐H2AX was significantly decreased in ALKBH5‐downregulated cells (Figure S4B,C). Moreover, apoptosis of ALKBH5‐overexpressed CRC cells increased, whereas apoptosis was significantly reduced in ALKBH5‐downregulated CRC cells compared to control cells (Figure S4D). Together, these results indicate that ALKBH5 could increase the radiosensitivity of CRC cells.

Above, we showed that the expression of circAFF2 is associated with m6A. In addition, both ALKBH5 and circAFF2 impact radiosensitivity. Next, we sought to investigate whether circAFF2 may regulate ALKBH5 expression or vice versa. First, we overexpressed or downregulated ALKBH5 in CRC cells and examined circAFF2 levels. As shown in Figure 4D, the expression of circAFF2 was significantly increased in ALKBH5‐overexpressed CRC cell, whereas expression of circAFF2 was significantly decreased in ALKBH5‐depleted CRC cells compared to control cells. However, alterations in circAFF2 levels had no effects on the levels of m6A and ALKBH5 in CRC cells (Figure S5A,C), which was consistent in grafted tumours (Figure S5D,E). Furthermore, Me‐RIP‐qPCR showed that potential m6A‐modified segments of circAFF2 could be enriched by anti‐m6A rather than anti‐immunoglobulin G. ALKBH5 downregulation exhibited a significantly increase in the circAFF2 m6A modification levels (Figure 4E).

Next, we sought to explore whether the regulatory effect of ALKBH5 on circAFF2 depends on m6A methylation and identify specific m6A methylation sites of circAFF2. We utilised SRAMP software (http://www.cuilab.cn/sramp/) to predict the five possible m6A methylation sites of circAFF2 (Figure S6). Based on the predicted sites, construct luciferase reporter plasmids were constructed by inserting partial sequence of wild‐type circAFF2 or circAFF2 with mutant m6A methylation sites (MUT1: mutation site 367; MUT2: mutation site 404; MUT3: mutation site 707; MUT4: mutation site 719; MUT5: mutation site 823) (Figure 4F). The luciferase activity of CRC cells transfected with the wild‐type plasmid was attenuated, while the luciferase activity of cells transfected with MUT5 circAFF2 remained unchanged upon ALKBH5 downregulation (Figure 4G). Therefore, our data suggest that the regulation of circAFF2 by ALKBH5 depends on m6A methylation, and the main m6A methylation site of circAFF2 is 823.

**FIGURE 5:**
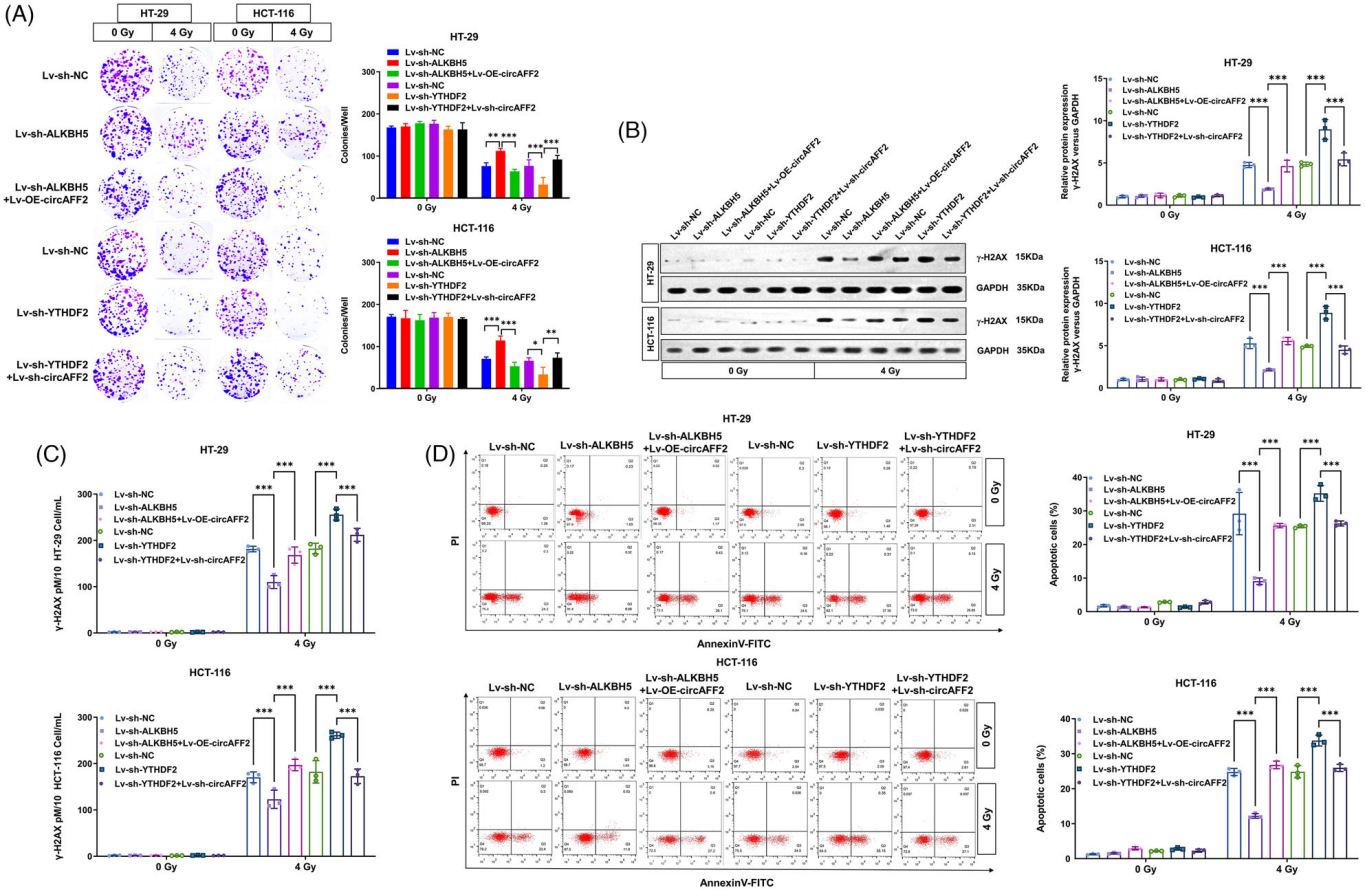
ALKBH5/YTHDF2 regulates colorectal cancer (CRC) radiosensitivity through circAFF2 in vitro. (A) Colony formation assay to detect the colony formation number of cells after 4 Gy irradiation in HT‐29 and HCT‐116 cells transfected with Len‐sh‐NC, Len‐sh‐ALKBH5, Len‐sh‐ALKBH5 + Len‐OE‐circAFF2, Len‐sh‐YTHDF2 and Len‐sh‐YTHDF2 + Len‐sh‐circAFF2. Western blot (B) and ELISA assay (C) to determine γ‐H2AX expression 6 h after 4 Gy irradiation in HT‐29 and HCT‐116 cells transfected with Len‐sh‐NC, Len‐sh‐ALKBH5, Len‐sh‐ALKBH5 + Len‐OE‐circAFF2, Len‐sh‐YTHDF2 and Len‐sh‐YTHDF2 + Len‐sh‐circAFF2. (D) One day after 4 Gy irradiation, apoptosis was determined in HT‐29 and HCT‐116 cells transfected with Len‐sh‐NC, Len‐sh‐ALKBH5, Len‐sh‐ALKBH5 + Len‐OE‐circAFF2, Len‐sh‐YTHDF2 and Len‐sh‐YTHDF2 + Len‐sh‐circAFF2 by flow cytometry. ^***^ *p* < .001, ^**^ *p* < .01, ^*^ *p* < .05.

**FIGURE 6:**
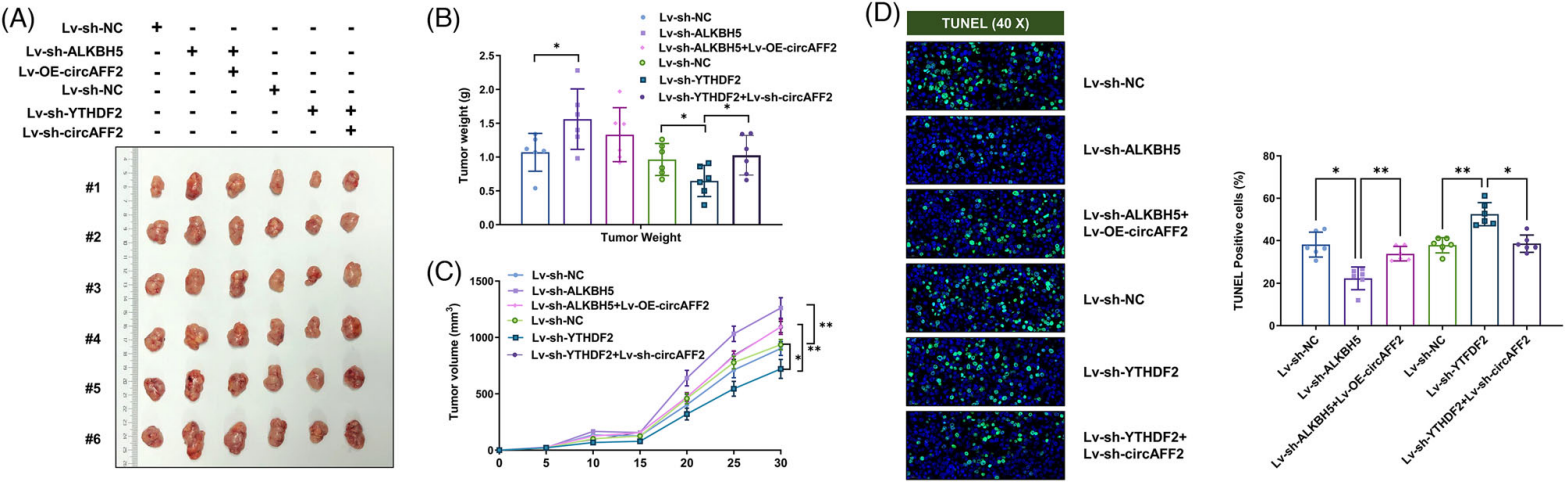
ALKBH5/YTHDF2 regulates colorectal cancer (CRC) radiosensitivity through circAFF2 in vivo. (A) After the tumour‐bearing mice were sacrificed, the subcutaneous tumours were collected for measurement. (B) The weight of the subcutaneous tumours in the tumour‐bearing mice. (C) The growth curve of the subcutaneous tumours in the tumour‐bearing mice. (D) The tumour tissue in the mice detect the expression of tunnel. ^***^ *p* < .001, ^**^ *p* < .01, ^*^ *p* < .05.

The main m6A‐related reader proteins are YTHDF1/2/3, YTHDC1/2 and IGF2BP1/2/3; among these, YTHDF2 has been widely involved in regulating the stability of m6A‐containing mRNA.^17^, ^20^ Recent studies have also demonstrated that YTHDF2 can initiate the decay of m6A‐containing circRNA.^18^, ^19^, ^32^ We observed a dramatic upregulation of circAFF2 expression in YTHDF2‐downregulated HCT‐116 and HT‐29 cells (Figure 4H). Moreover, we observed that knockdown of YTHDF2 could reverse the downregulation of circAFF2 caused by ALKBH5 knockdown in HCT‐116 and HT‐29 cells (Figure 4I), indicating that the expression of circAFF2 is regulated by ALKBH5‐mediated demethylation, while YTHDF2 mediates circAFF2 degradation in an m6A‐dependent manner. In vitro radiosensitivity rescue experiments showed that the radiosensitivity induced by ALKBH5 or YTHDF2 could be reversed by circAFF2 (Figure 5A,D), which was confirmed in grafted tumours (Figure 6A,D). Collectively, these data suggest that ALKBH5/YTHDF2 regulates the radiosensitivity of CRC through circAFF2.

### 3.4 CircAFF2 inhibits Cullin neddylation by binding with CAND1

**FIGURE 7:**
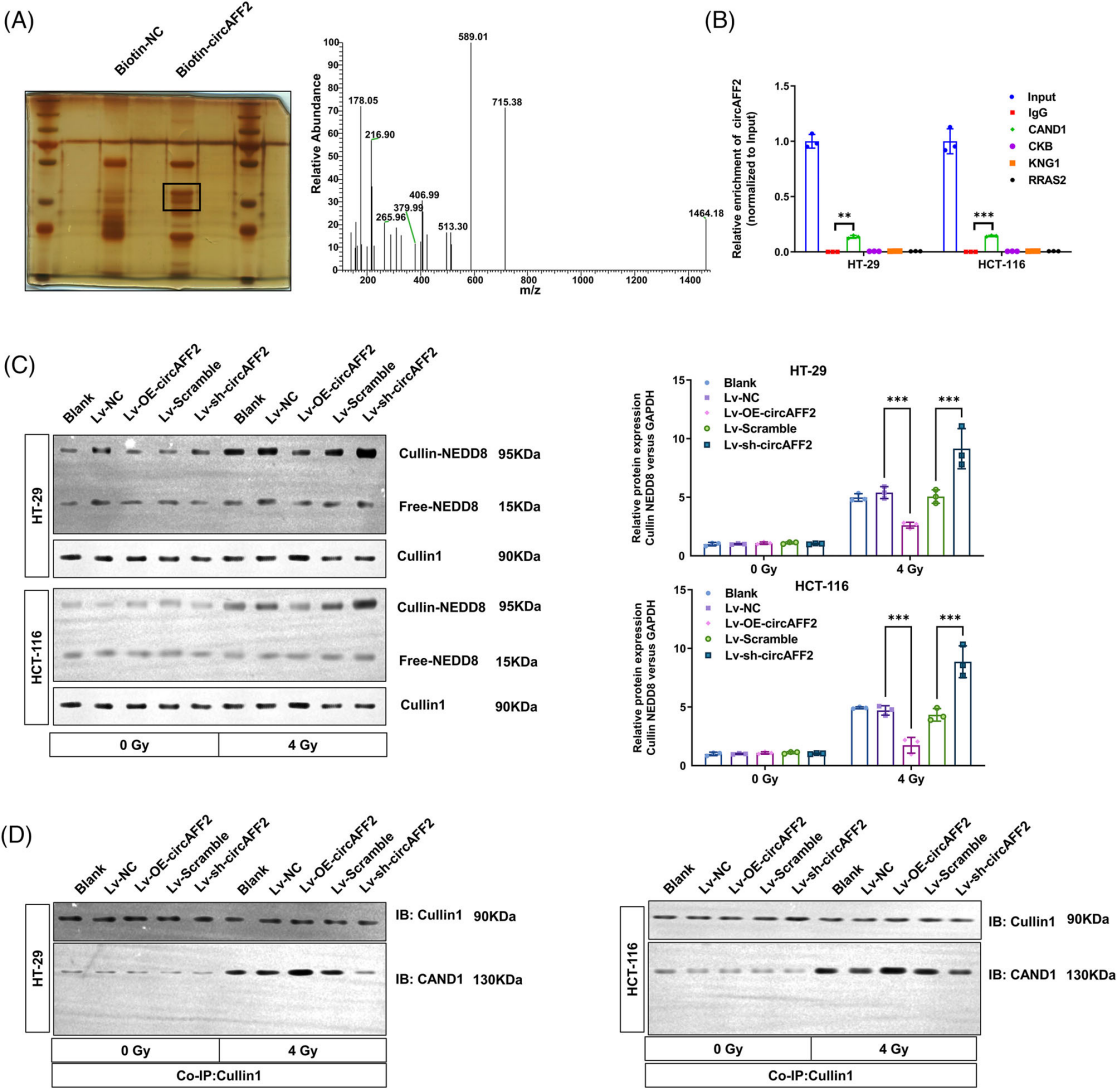
CircAFF2 inhibits Cullin neddylation by binding to CAND1. (A) RNA pull‐down and liquid chromatography–mass spectrometry (LC–MS) were used to detect possible circAFF2 binding proteins. (B) RNA immunoprecipitation (RIP) validates the binding of CAND1 with circAFF2. (C) Neddylation assay to detect Cullin‐neddylation level in circAFF2‐overexpressed or circAFF2‐depleted HT‐29 and HCT‐cells before and after irradiation. (D) Co‐immunoprecipitation (co‐IP) to detect binding levels of Cullin1–CAND1 in circAFF2‐overexpressed or circAFF2‐depleted HT‐29 and HCT‐116 cells before and after irradiation. ^***^ *p* < .001, ^**^ *p* < .01, ^*^ *p* < .05.

To study the downstream signaling pathways of circAFF2 in CRC cells, we performed RNA pull‐down experiments followed by mass spectrometry analysis and found 843 proteins that may bind to circAFF2 in HCT‐116 CRC cells (Figure 7A and Table S4). According to the RNA–protein interaction prediction website (http://pridb.gdcb.iastate.edu/RPISeq/) and literature review, CAND1, RRAS2, CKB and KNG1 were selected as candidate circAFF2‐binding proteins (Figure S7). We then performed RIP assay to evaluate the binding capacity of CAND1, RRAS2, CKB and KNG1 to circAFF2. As shown in Figure 7B, CAND1 was the only protein that bound to circAFF2. CAND1 has been shown to inhibit cellular neddylation modification by binding to CRL.^26^, ^27^, ^28^ Additionally, in the circAFF2 RNA pull‐down–liquid chromatography/mass spectrometry analysis, detectable enrichment of Cullin1 was found in the circAFF2 pull‐downed components, whereas no other Cullin subtypes were found (Table S4). Overexpression or downregulation of circAFF2 expression in HCT‐116 and HT‐29 CRC cells had no significant effects on CAND1 mRNA and protein expression both before and after irradiation (Figure S8). However, Cullin‐neddylation levels in radiation‐exposed cells were increased, which was significantly inhibited by circAFF2 overexpression and increased by circAFF2 knockdown (Figure 7C), suggesting that the interaction between circAFF2 and CAND1 can promote neddylation in CRC cells. Immunoprecipitation data showed that overexpression of circAFF2 could promote the binding of Cullin1 and CAND1, while downregulation of circAFF2 could inhibit the binding of Cullin1 and CAND1 (Figure 7D). Taken together, our data suggest that circAFF2 inhibits Cullin neddylation by promoting the binding of CAND1 and Cullin1.

**FIGURE 8:**
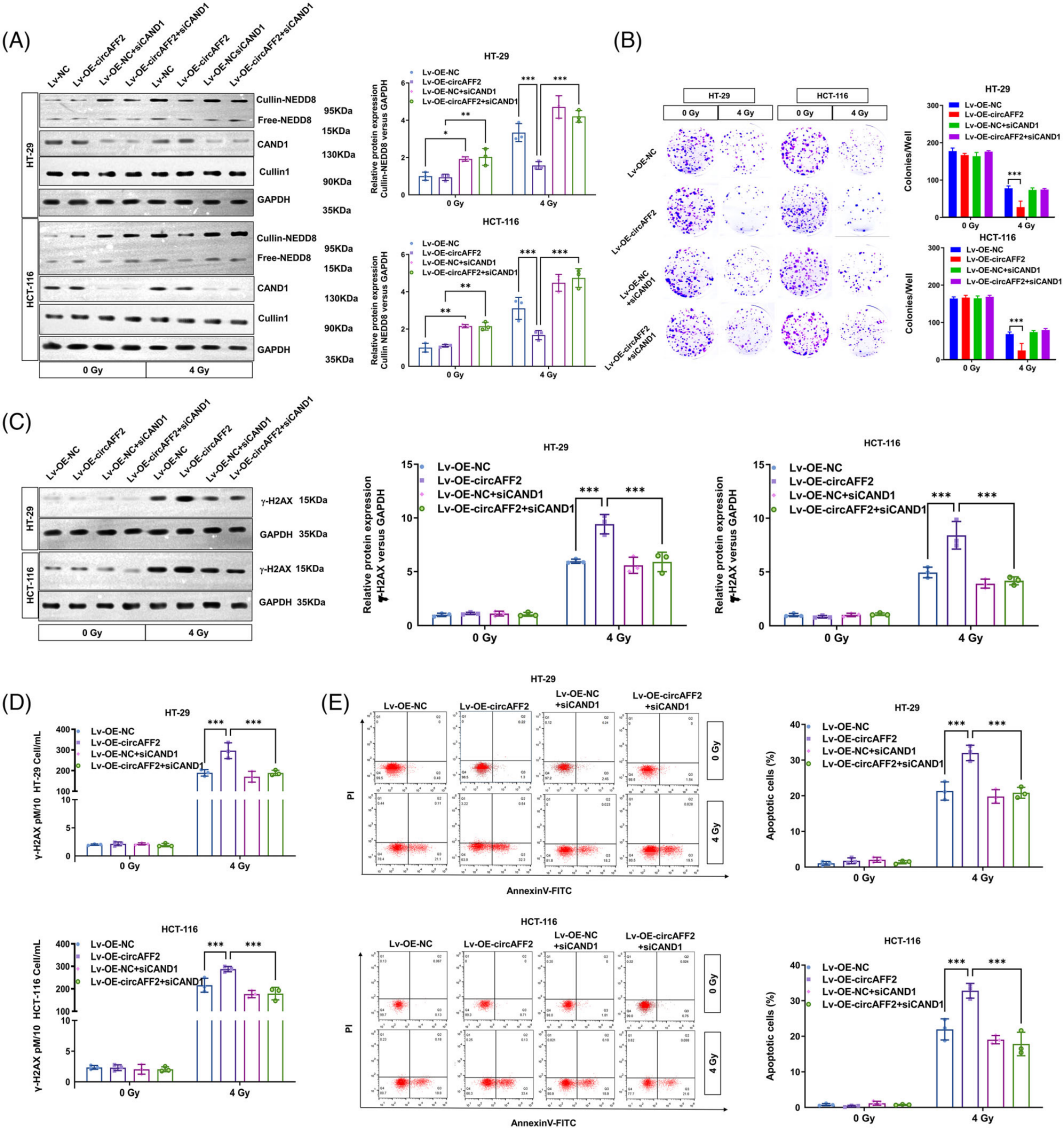
CircAFF2 regulates the radiosensitivity of colorectal cancer (CRC) by binding to CAND1. (A) Neddylation assay to detect Cullin‐neddylation level in HT‐29 and HCT‐116 cells transfected with Len‐OE‐NC, Len‐OE‐circAFF2, Len‐OE‐NC + siCAND and Len‐OE‐circAFF2 + siCAND1. (B) Colony formation assay to detect the colony formation number of cells after 4 Gy irradiation in HT‐29 and HCT‐116 cells transfected with Len‐OE‐NC, Len‐OE‐circAFF2, Len‐OE‐NC + siCAND and Len‐OE‐circAFF2 + siCAND1. Western blot (C) and ELISA assay (D) to determine the γ‐H2AX expression in HT‐29 and HCT‐116 cells transfected with Len‐OE‐NC, Len‐OE‐circAFF2, Len‐OE‐NC + siCAND and Len‐OE‐circAFF2 + siCAND1. (E) Flow cytometry to detect the apoptosis of HT‐29 and HCT‐116 cells transfected with Len‐OE‐NC, Len‐OE‐circAFF2, Len‐OE‐NC + siCAND and Len‐OE‐circAFF2 + siCAND1 1 day after 4 Gy irradiation. ^***^ *p* < .001, ^**^ *p* < .01, ^*^ *p* < .05.

Neddylation is a post‐translational protein modification that exerts a critical role in tumour development and radiosensitivity.^22^, ^23^, ^24^ To investigate the effect of neddylation on the radiosensitivity of CRC, we treated CRC cells with MLN4924, a neddylation inhibitor. We found that inhibition of neddylation strongly increased the radiosensitivity of CRC cells (Figure S9). We further performed rescue experiments to investigate whether circAFF2 enhances the radiosensitivity of CRC by promoting the binding of CAND1 to Cullin1. The circAFF2 overexpression reduced Cullin‐neddylation levels were restored by CAND1 silence (Figure 8A). Cloning formation assays, western blotting, ELISA assays and flow cytometry were used to demonstrate that CAND1 knockdown can restore the enhanced radiosensitivity caused by circAFF2 overexpression to the level of the control group (Figure 8B,E). The effects of circAFF2 on inhibiting neddylation and increasing radiosensitivity were eliminated in CAND1‐knockout CRC cells. Together, these data demonstrated that circAFF2 increased the radiosensitivity of CRC via promoting the binding between CAND1 and Cullin1, which led to the inhibition of Cullin neddylation. With the knockdown of CAND1, the Cullin neddylation was activated, which resulted a decreased radiosensitivity of CRC.

### 3.5 Expression and clinical significance of circAFF2 in rectal cancer patients with neoadjuvant chemoradiotherapy

**FIGURE 9:**
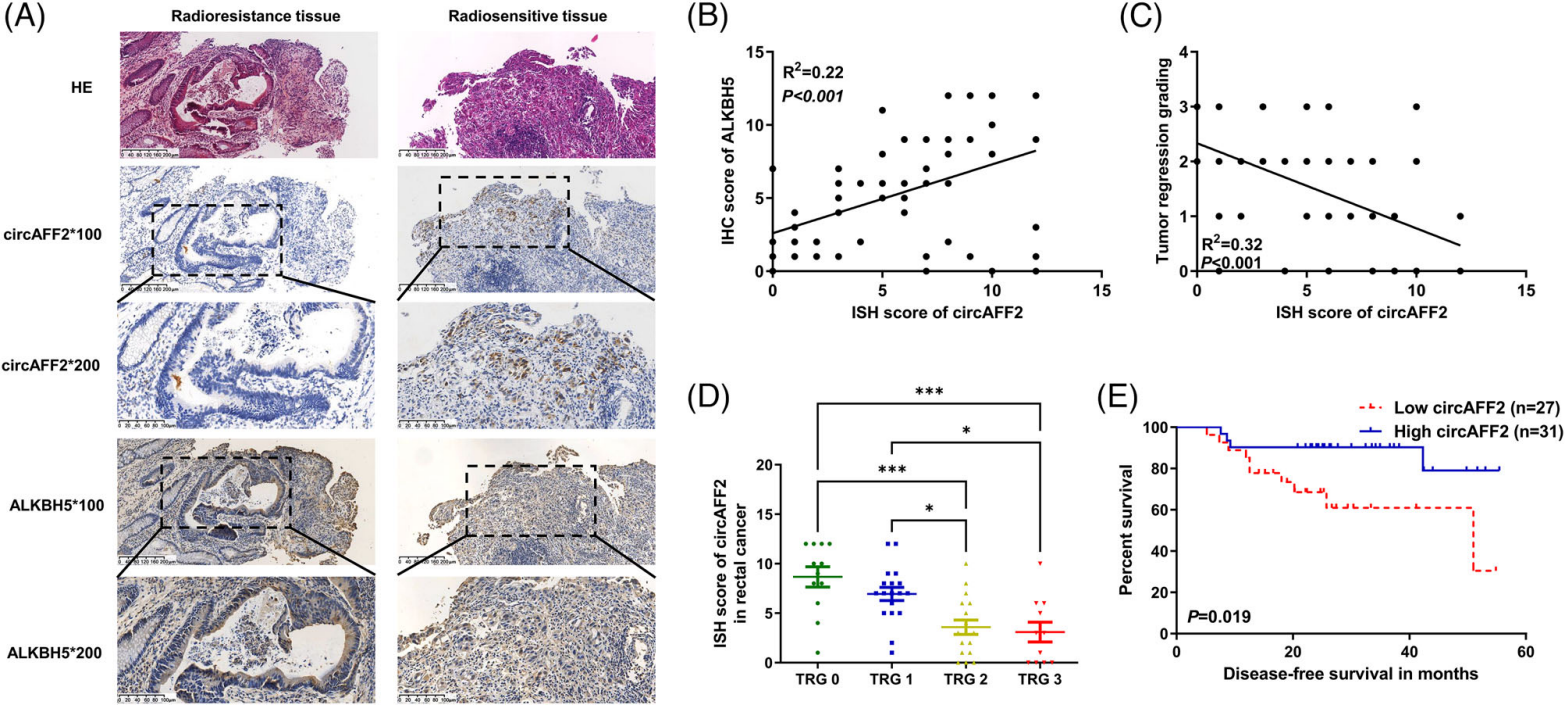
Expression and clinical significance of circAFF2 in rectal cancer patients with neoadjuvant chemoradiotherapy. (A) Representative images of in situ hybridisation (ISH) and immunohistochemistry (IHC) staining of circAFF2 and ALKBH5 in colorectal cancer (CRC) radioresistance and radiosensitive tissues. Scale bars, 100 and 200 μm, respectively. (B) CircAFF2 expression was positively correlated with ALKBH5 in CRC. (C) CircAFF2 expression was negatively correlated with tumour regression grading (TRG) score in CRC. (D) The expression of circAFF2 in CRC patients with TRG 0 and TRG 1 was significantly higher than that in TRG 2 and TRG 3. (E) Kaplan–Meier curve analysis showed that the disease‐free survival (DFS) of patients with high circAFF2 expression was significantly higher than that of patients with low circAFF2 expression. ^***^ *p* < .001, ^**^ *p* < .01, ^*^ *p* < .05.

Preoperative tissues were collected from 58 patients with locally advanced rectal cancer who underwent neoadjuvant chemoradiotherapy (radiation dose range of 4500–4800 cGy supplemented with single oral administration of capecitabine) and surgical removal. ISH assay was adopted to examine the expression of circAFF2 and IHC was used to evaluate ALKBH5 levels in the collected tissues. For all patients, the median age was 60 years, 79% were male and 91% had low‐to‐moderate rectal cancer. Twenty‐five (43%) and 33 (57%) patients were clinically stage II and III before neoadjuvant chemoradiotherapy, respectively. A total of 12 patients showed pCR by postoperative pathology. Compared with the clinical staging before neoadjuvant chemoradiotherapy, 36 cases were pathologically staged down after the operation. Postoperative pathology showed 12 cases (20.6%), 18 cases (32.8%), 17 cases (29.3%) and 11 cases (19.0%) in TRG = 0, 1, 2 and 3, respectively. The findings of ISH and IHC demonstrated that the expression of circAFF2 and ALKBH5 was high in radiosensitive CRC tissues and low in radioresistant tissues (Figure 9A). Moreover, the expression of circAFF2 positively correlated with ALKBH5 (Figure 9B), while negatively correlated with TRG score (Figure 9C). Specifically, patients with TRG 0−1 exhibited a notably higher expression of circAFF2 than those with TRG 2−3 (Figure 9D). An ISH score of circAFF2 ≥6 was defined as high expression, and Kaplan–Meier curve analysis showed that the disease‐free survival of patients with elevated circAFF2 levels was significantly higher than that of patients with relatively low circAFF2 expression (Figure 9E). Collectively, our data suggest that circAFF2 can be used as an indicator for screening and efficacy evaluation of sensitive populations before concurrent chemoradiotherapy for rectal cancer.

## 4 DISCUSSION

Neoadjuvant chemoradiotherapy in the treatment of locally advanced rectal cancer can not only improve the local tumour control rate, but also increase the chance of preserving the anal sphincter. However, the efficacy of preoperative radiotherapy for rectal cancer varies among patients. Specifically, patients with obvious response, partial response and insignificant response to radiotherapy accounted for 33%. Notably, patients with insignificant responses benefited less from neoadjuvant radiotherapy. Moreover, preoperative radiotherapy for patients with no obvious response can lead to disease progression during treatment, thereby delaying surgical treatment. Therefore, it is imperative to identify molecular biomarkers associated with radiosensitivity of rectal cancer.

Recent studies have revealed a close association between circRNAs and tumour radiosensitivity.^33^ For instance, Xia et al.^34^ reported that circ‐AKT3 inhibits the phosphorylation of AKT‐thr308 by encoding AKT3‐174aa, thereby improving the radiosensitivity of glioma cells. In addition, 2600 differentially expressed circRNAs have been identified in nasopharyngeal carcinoma radioresistant cell lines, among which hsa_circRNA_006660 enhanced radiosensitivity of nasopharyngeal carcinoma by adsorbing miR‐1276.^35^ In oral squamous carcinoma cells, the sponging action of circANTRL1 on miR‐23a‐3p may lead to an increase in PTEN expression and a consequent enhancement of radiosensitivity.^36^ In esophageal cancer radioresistant cell line KYSE‐150R, 74 differentially expressed circRNAs were found, of which 57 were upregulated and 17 were downregulated; these differential circRNAs mainly regulate the Wnt pathway.^37^ Exosome‐mediated knockout of circ_0067835 in CRC cells inhibited cell proliferation and cell cycle progression, enhancing apoptosis and radiosensitivity.^38^ Furthermore, circ_IFT80 acts as a sponge for miR‐296‐5p and suppresses the radiosensitivity of CRC by upregulating the expression of MSI1.^39^ In this study, differentially expressed circRNAs were screened in radiosensitive and radioresistant rectal cancer tissues and circAFF2 was selected for further investigation. It has been found that circAFF2 plays different roles in different tumours. For example, circAFF2 was downregulated in non‐small cell lung cancer,^40^ and acted as a sponge for miR‐661, inhibiting tumour progression by enhancing the expression of DOK7.^40^ In gastric cancer, circAFF2 upregulates ANTXR 1 expression by adsorbing miR‐6894‐5p, thereby promoting tumour proliferation.^41^ Additionally, circAFF2 is highly expressed in renal cell carcinoma (RCC), and high expression patients with RCC had a poor prognosis.^42^ Here, we revealed that patients with radiosensitive rectal cancer exhibited elevated levels of circAFF2 expression, and those with high circAFF2 expression had a more favourable prognosis. In addition, circAFF2 enhanced the radiosensitivity of CRC cells both in vitro and in vivo.

As the most common modification of eukaryotic RNA, the dynamic regulation of m6A methylation is governed by three regulatory factors, methyltransferase, demethylase and reader proteins that participate in RNA translation, mRNA stability and mRNA splicing.^43^ As one of the two main demethylases, ALKBH5 has been confirmed to be closely related to CRC.^13^, ^14^, ^15^, ^44^ Studies have shown reduced expression of ALKBH5 in CRC, and low expression of ALKBH5 in CRC patients predicted poor prognosis.^14^, ^15^ In addition, knockdown of ALKBH5 significantly enhanced the proliferation, migration and invasion of CRC.^15^ Wu et al.^44^ also showed similar results. These results confirmed the role of ALKBH5 as a tumour suppressor in CRC. However, ALKBH5 has been reported to promote cancer cell proliferation by demethylating the lncRNA NEAT1.^13^ Therefore, the function of ALKBH5 in CRC is controversial and unclear. Here, we found that ALKBH5 was highly expressed in radiosensitive rectal cancer tissues and expressed at low levels in radioresistant tissues. Furthermore, ALKBH5 significantly increased the radiosensitivity of CRC in vitro and in vivo, supporting the suppressive role of ALKBH5 in CRC.

Recently, circRNA m6A methylation modification has been demonstrated to play an important regulatory role in tumours.^29^ Previous studies have reported that m6A modification of circRNAs is involved in the nuclear export, translation and degradation of circRNAs.^18^, ^19^, ^45^, ^46^ YTHDF2 is an important m6A modification reader, and its primary function is to regulate the stability of m6A‐modified mRNA.^17^ Zhou et al. demonstrated that YTHDF2 could promote the degradation of m6A‐modified circRNAs.^19^ Furthermore, according to a recent study, the YTHDF2–HRSP12–RNase P/MRP axis can cleave m6A‐modified circRNAs.^18^ The co‐IP experiment revealed that HRSP12 played a role in linking YTHDF2 and RNase P/MRP. RNase P/MRP is an endoribonuclease that connects to YTHDF2 through HRSP12 to form a YTHDF2–HRSP12–RNase P/MRP complex, which cleaves circRNA.^18^ The current study confirmed that ALKBH5 could regulate the m6A modification of circAFF2, while YTHDF2 could promote the degradation of m6A‐modified circAFF2.

Neddylation is a novel post‐translational modification catalysed by NEDD8‐activating enzyme, conjugating enzyme and ligase successively, leading to the conjugation of NEDD8 to different substrates.^47^ The Cullin family is the most widely studied substrate for neddylation and is the backbone protein in the CRL E3 ubiquitin ligase complex.^48^ Neddylation is closely associated with the radiosensitivity of many tumours.^22^, ^24^, ^49^ For instance, in pancreatic cancer and head and neck squamous cell carcinoma, neddylation inhibitors can effectively inhibit Cullin neddylation in vitro and in vivo and promote the radiosensitivity of pancreatic cancer cells.^22^, ^49^ Moreover, neddylation inhibitors enhance radiation‐induced G2/M arrest, apoptosis and DNA damage responses in CRC by promoting p27 expression.^50^ Here, we uncovered a novel role of circAFF2 in promoting the radiosensitivity of CRC cells by enhancing the interaction of CAND1 and Cullin1, thereby effectively inhibiting Cullin1 neddylation.

However, there are still some limitations in this study. First, our study only described the downstream genes regulated by m6A and did not examine the upstream regulation of m6A methylation. Second, we recognise that the ways in which cricAFF2 and Cullin1 interact with each other may extend beyond just binding to RBPs. Additional mechanisms should be explored to fully elucidate their relationship. Therefore, it may be necessary to explore such mechanisms in the future. In addition, for screening and evaluating the expression and clinical significance of circAFF2 in rectal cancer, we cannot exclude the effects of chemotherapy on the expression of circAFF2.

**FIGURE 10:**
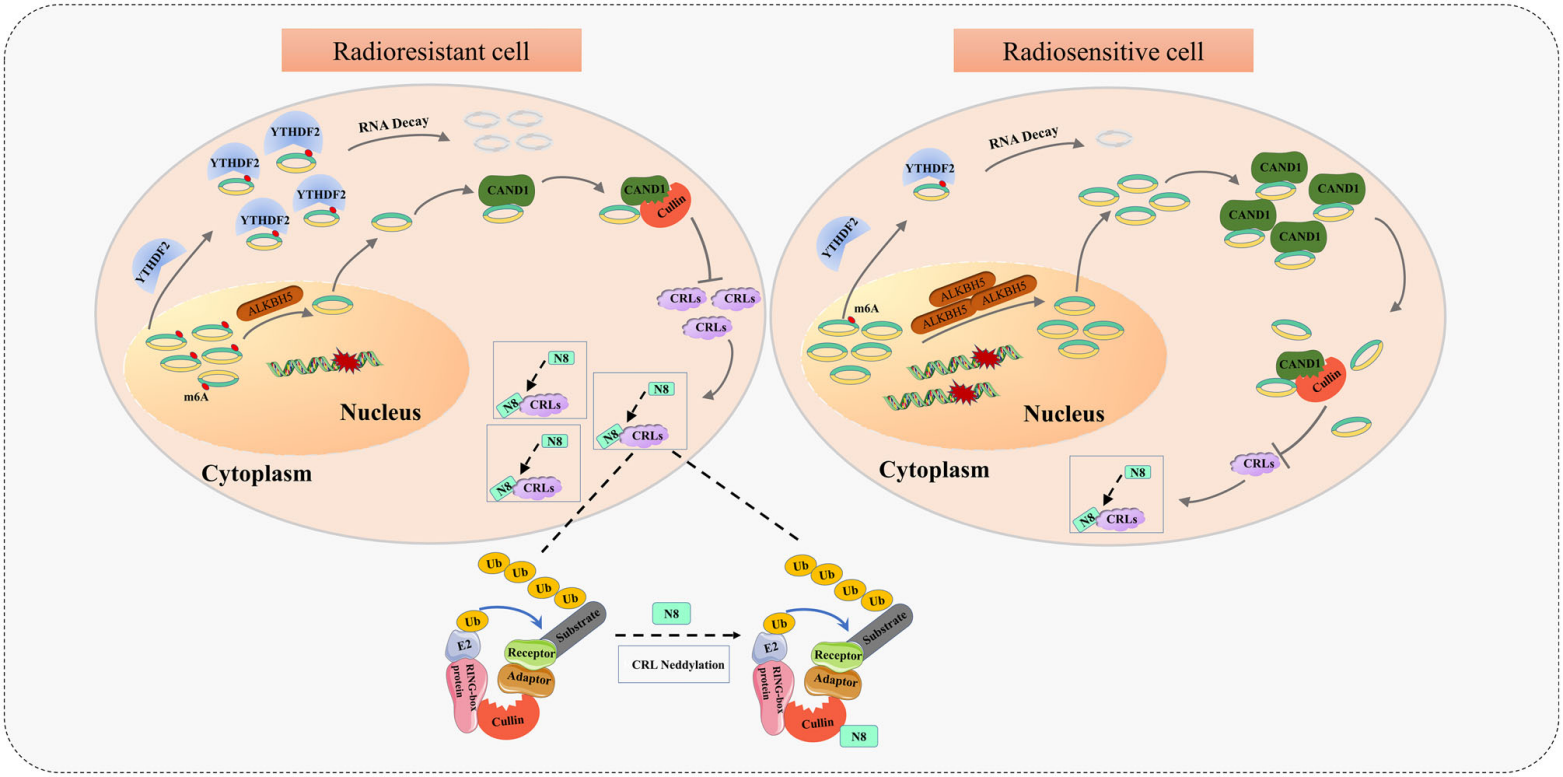
Schematic diagram depicting the proposed mechanisms of circAFF2 in colorectal cancer (CRC).

In conclusion, we identified and characterised circAFF2 as a novel m6A‐regulated circRNA in CRC. Mechanistically, circAFF2 expression is regulated by ALKBH5‐mediated demethylation, and YTHDF2 mainly mediates its degradation in an m6A‐dependent manner. CircAFF2 enhanced the radiosensitivity of CRC cells by binding to CAND1 and promoting the interaction of CAND1 to Cullin1, thereby inhibiting the neddylation modification in CRC cells (Figure 10). This study reveals the regulatory mechanism of ALKBH5/YTHDF2/circAFF2/CAND1/Cullin neddylation, which in turn affects the radiosensitivity of CRC cells. Our study highlights that circAFF2 could be used to screen radiosensitive populations. Importantly, circAFF2 serves as a potential radiosensitisers for rectal cancer with preoperative concurrent chemoradiotherapy.

## CONFLICT OF INTEREST STATEMENT

The authors declare they have no conflicts of interest.

## Supporting information

Supporting Information

Supporting Information

Supporting Information

## Data Availability

RNA‐seq data were uploaded in the NCBI GEO database with accession number GSE186940 (https://www.ncbi.nlm.nih.gov/geo/query/acc.cgi?acc=GSE186940).
