# Supplementary material for: ALKBH5/YTHDF2‐mediated m6A modification of circAFF2 enhances radiosensitivity of colorectal cancer by inhibiting Cullin neddylation
Source: Clin Transl Med. 2023 Jun 28;13(7):e1318. doi: 10.1002/ctm2.1318 (PMC10307995; doi:10.1002/ctm2.1318)
Supplement: Supplementary file 1 — Supporting Information [file CTM2-13-e1318-s003.DOCX]

**Supplementary Figure 1** (A) The efficiency o
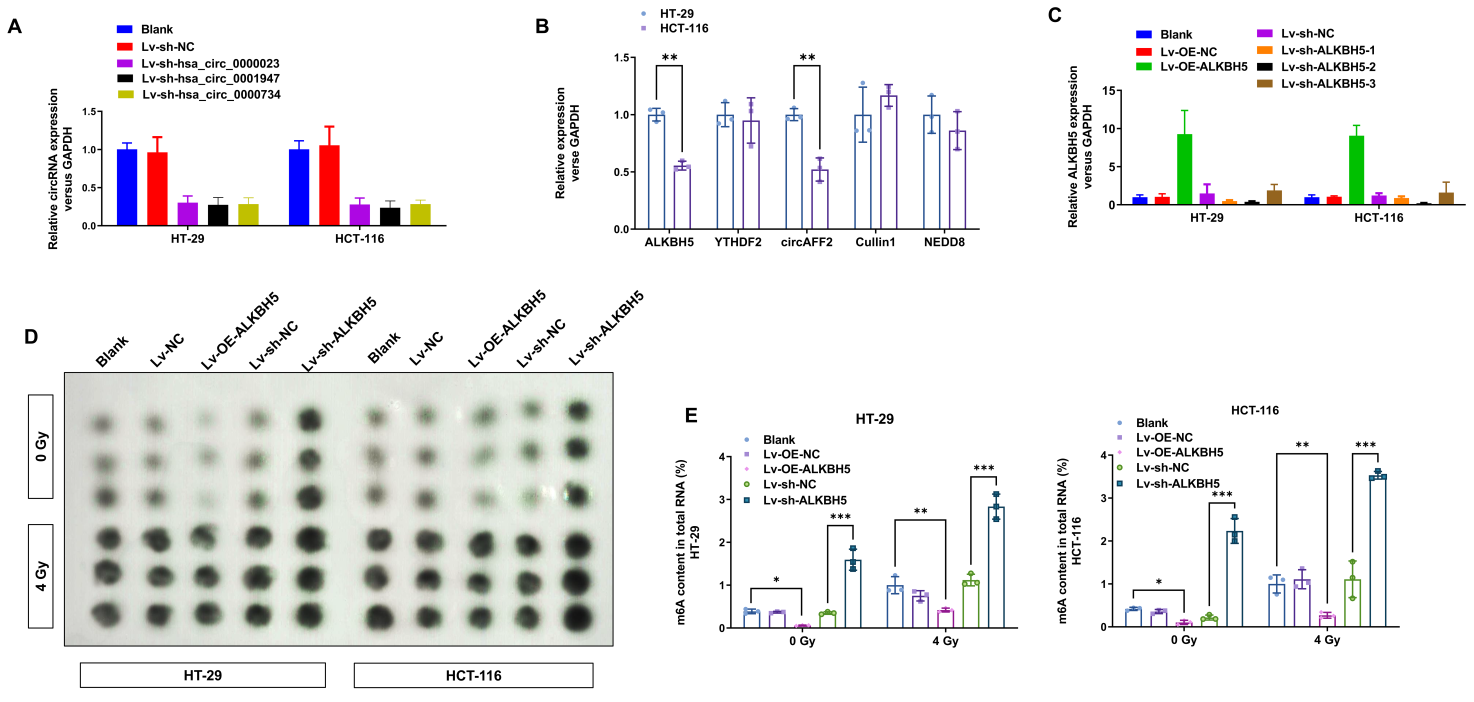
f hsa_circ_0000023, hsa_circ_0001947 or hsa_circ_0000734 down-regulation in HT-29 and HCT-116 cells was verified by qRT-PCR; (B) The expression of ALKBH5/YTHDF2/circAFF2/Cullin-NEDD8 were detected by qRT-PCR in HT-29 and HCT-116 cells; (C) The efficiency of ALKBH5 up-expression and down-expression in HT-29 and HCT-116 cells was verified by qRT-PCR; Dot blot (D) AND m6A Methylation Assay (E) detection of m6A levels in CRC cell lines after up- or down-regulation of ALKBH5. (****P* < 0.001, ***P* < 0.01, **P* < 0.05)

**
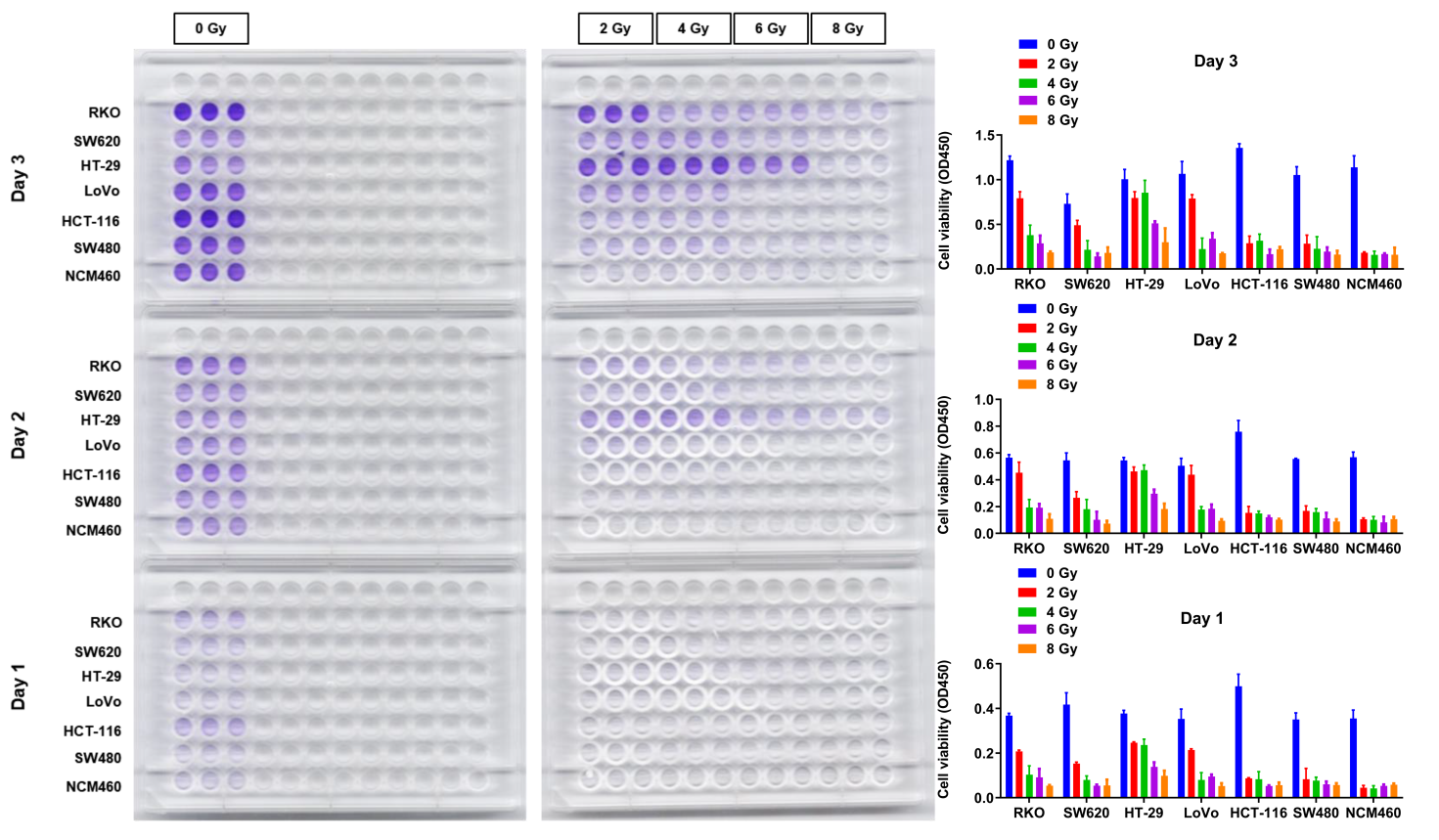
**

**Supplementary Figure 2** The radiosensitivity of RKO, sw480, HT-29, LoVo, HCT-116, SW620 and NCM460 under different irradiation doses detected by CCK-8.

**
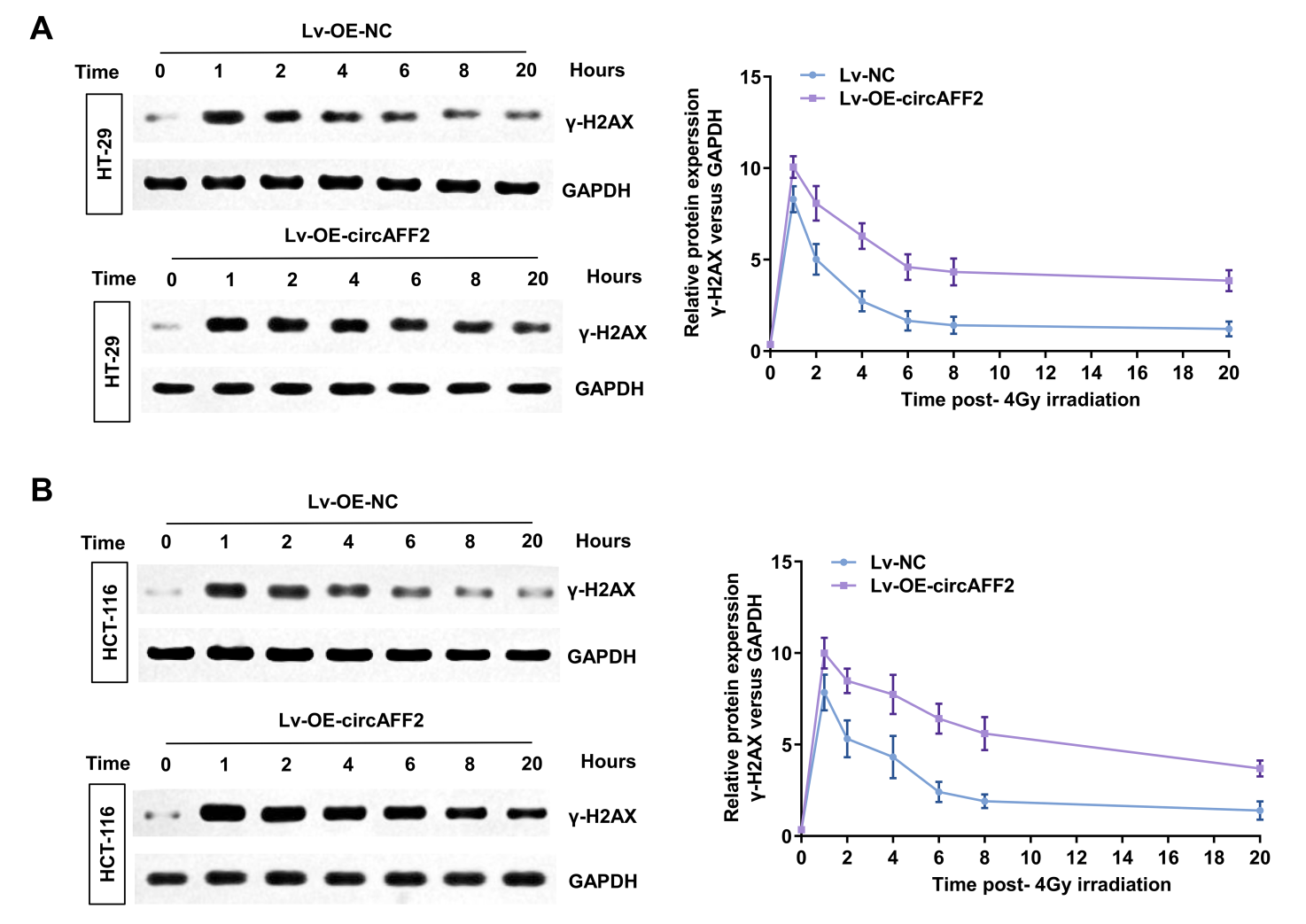
**

**Supplementary Figure 3** Western blot to detect the expression of γ-H2AX in HT-29 (A) and HCT-116 (B) cells at different time points (0, 1, 2, 4, 6, 8, and 20 hours) after being exposed to 4Gy radiation.

**
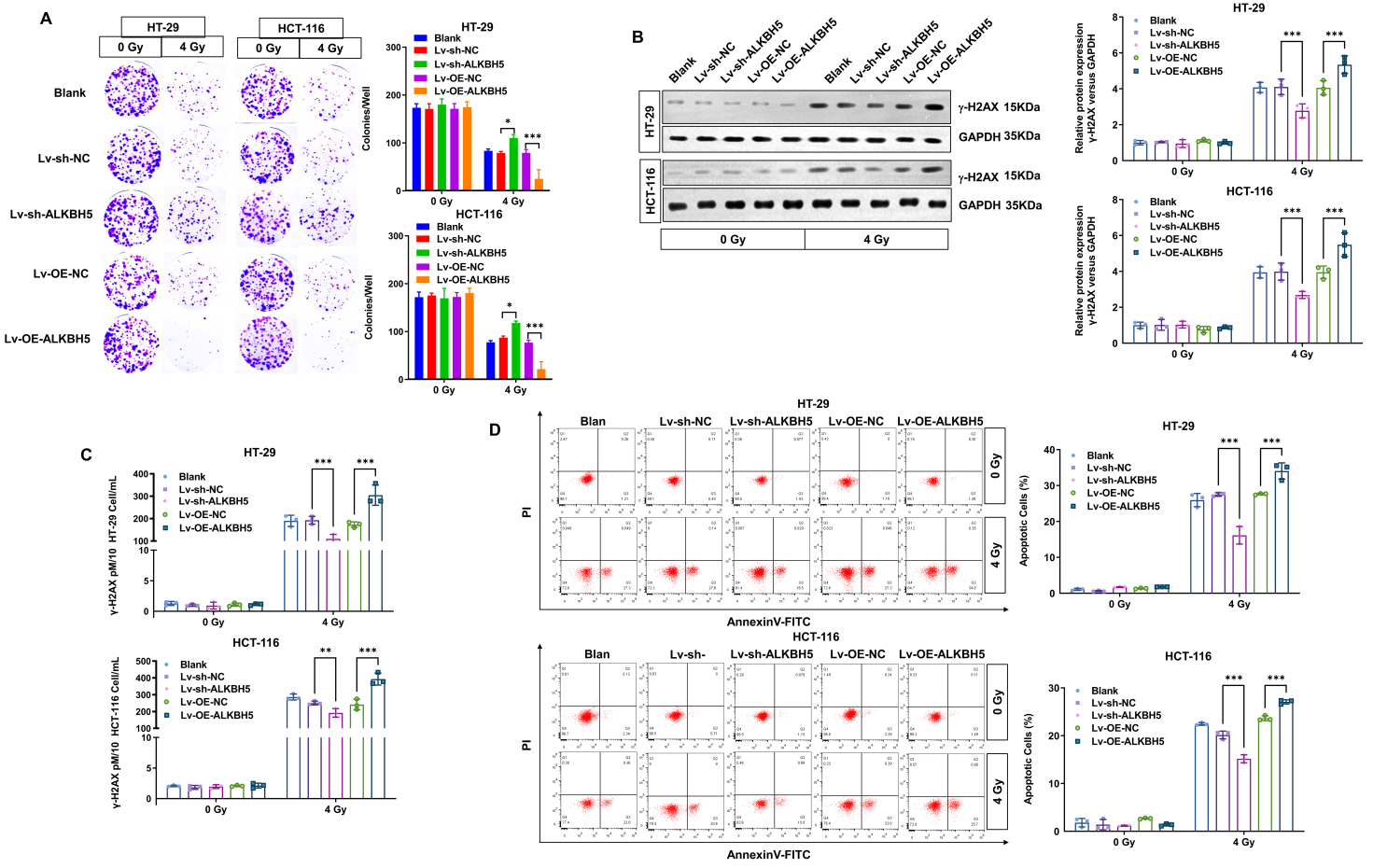
**

**Supplementary Figure 4** ALKBH5 increases the radiosensitivity of CRC cells. (A) Colony formation assay showing the number of clones in ALKBH5-overexpressed or ALKBH5-depleted HT-29 and HCT-116 cells after 4Gy irradiation. Western blot (B) and ELISA assay (C) to detect the expression of γ-H2AX in ALKBH5-overexpressed or ALKBH5-depleted HT-29 and HCT-116 cells after 4Gy irradiation for 6 hours. (D) One day after 4Gy irradiation, apoptosis of ALKBH5-overexpressed or ALKBH5-depleted HCT-116 and HT-29 cells was examined by flow cytometry. (****P* < 0.001, ***P* < 0.01, **P* < 0.05)

**
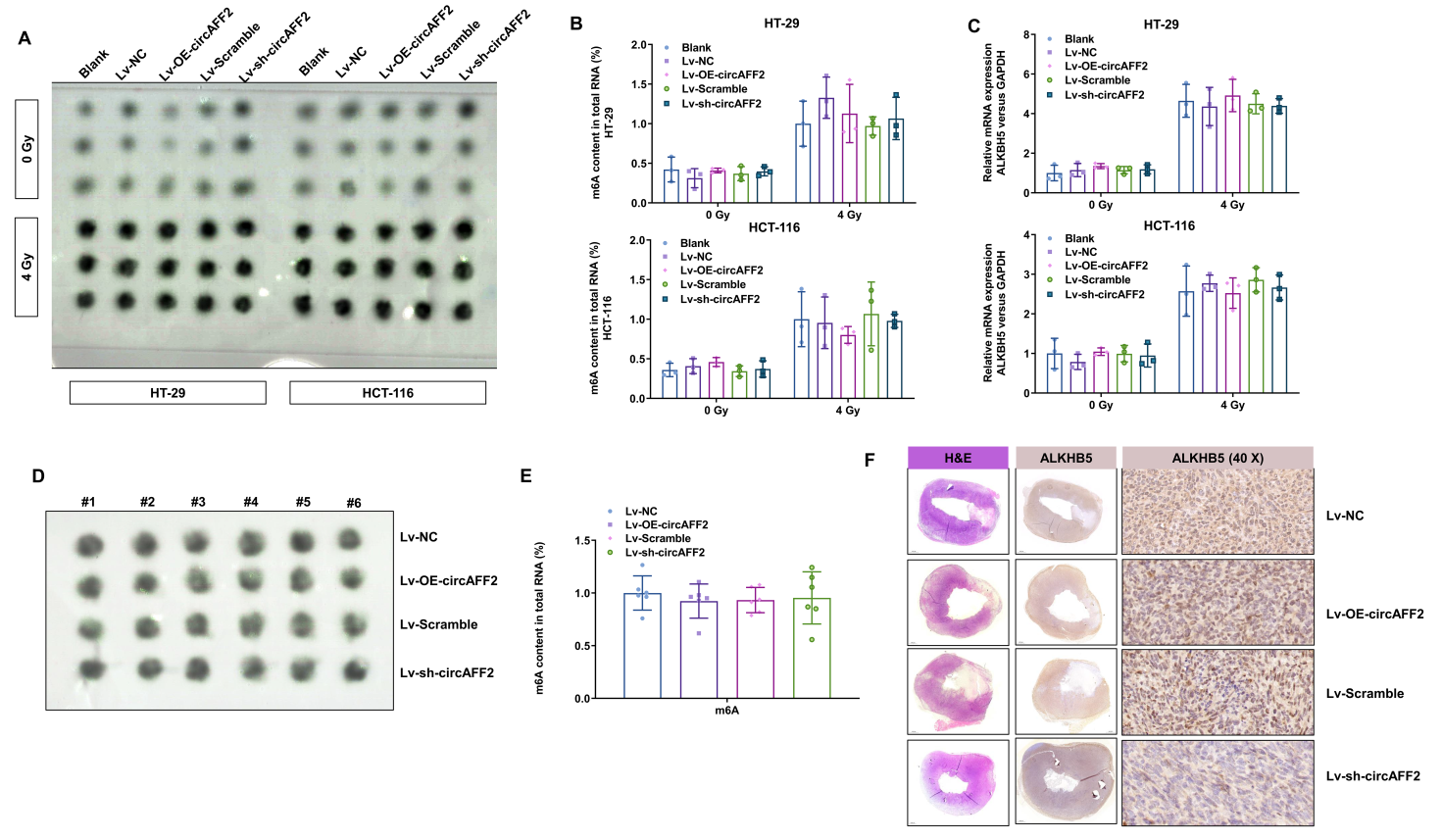
**

**Supplementary Figure 5** Dot blot (A) AND m6A Methylation Assay (B) detection of m6A levels in CRC cell lines after up- or down-regulation of circAFF2; (C) qRT-PCR detection of ALKBH5 expression in CRC cell lines after up- or down-regulation of circAFF2; Dot blot (D) AND m6A Methylation Assay (E) Detection of m6A levels in xenograft mouse models; (F) ICH detection of ALKBH5 expression in xenograft mouse models. (****P* < 0.001, ***P* < 0.01, **P* < 0.05)

**
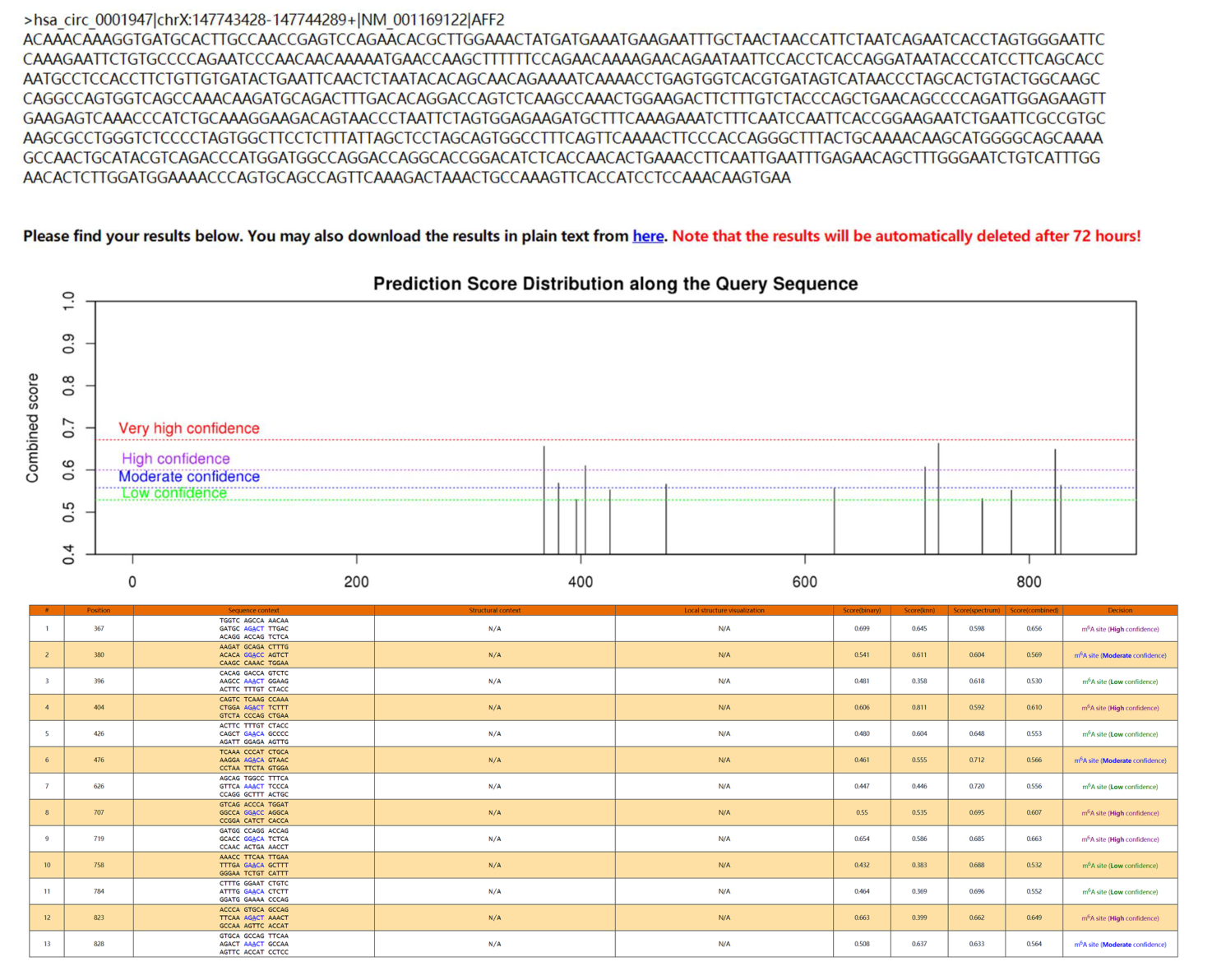
**

**Supplementary Figure 6** SRAMP software (http://www.cuilab.cn/sramp/) predicted the possible m6A methylation sites of circAFF2.

**
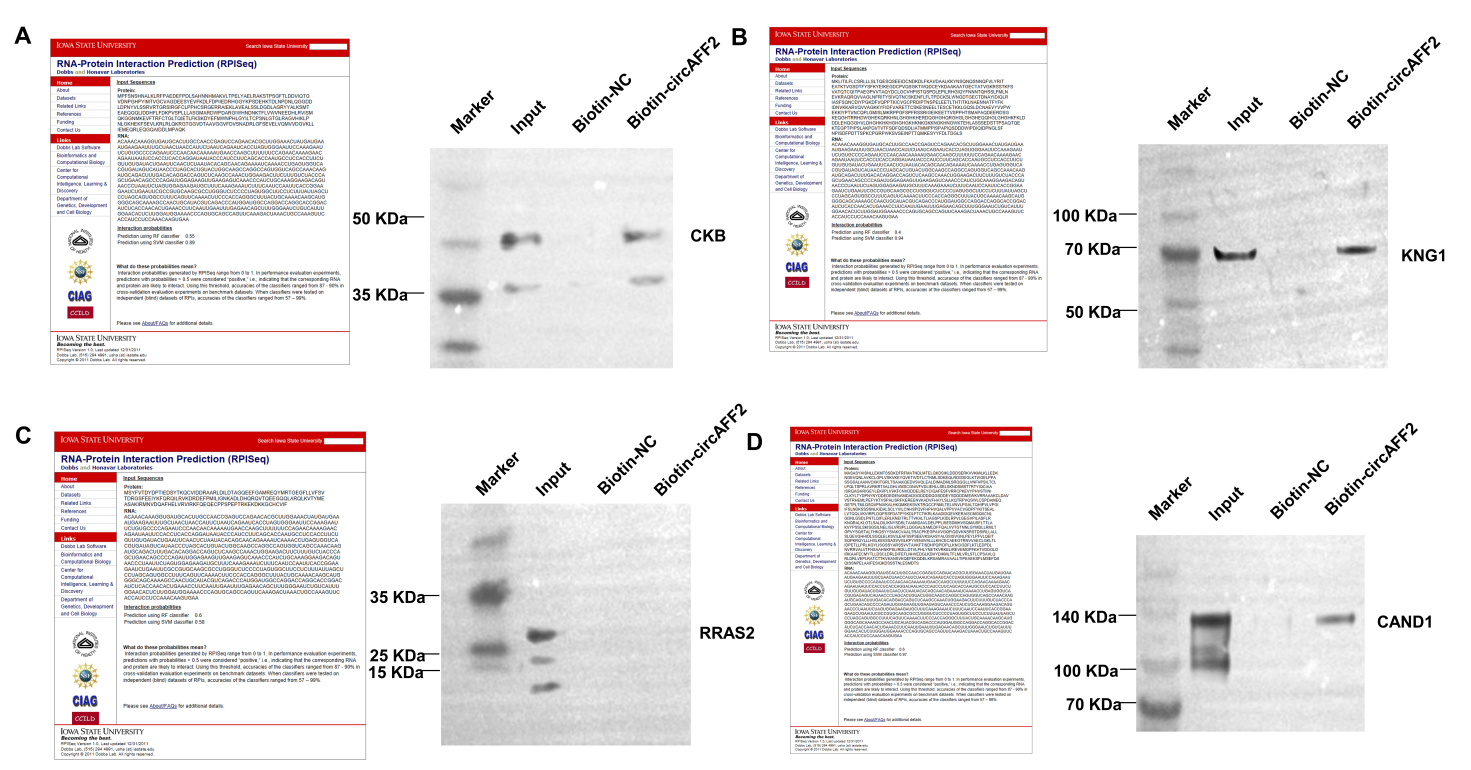
**

**Supplementary Figure 7** (A) RNA-protein interaction prediction (http://pridb.gdcb.iastate.edu/RPISeq/) predicts the binding fraction of circAFF2 to CKB and is verified by RNA pull down; (B) RNA-protein interaction prediction (http://pridb.gdcb.iastate.edu/RPISeq/) predicts the binding fraction of circAFF2 to KNG1 and is verified by RNA pull down; (C) RNA-protein interaction prediction (http://pridb.gdcb.iastate.edu/RPISeq/) predicts the binding fraction of circAFF2 to RRAS2 and is verified by RNA pull down; (D) RNA-protein interaction prediction (http://pridb.gdcb.iastate.edu/RPISeq/) predicts the binding fraction of circAFF2 to CAND1 and is verified by RNA pull down.

**
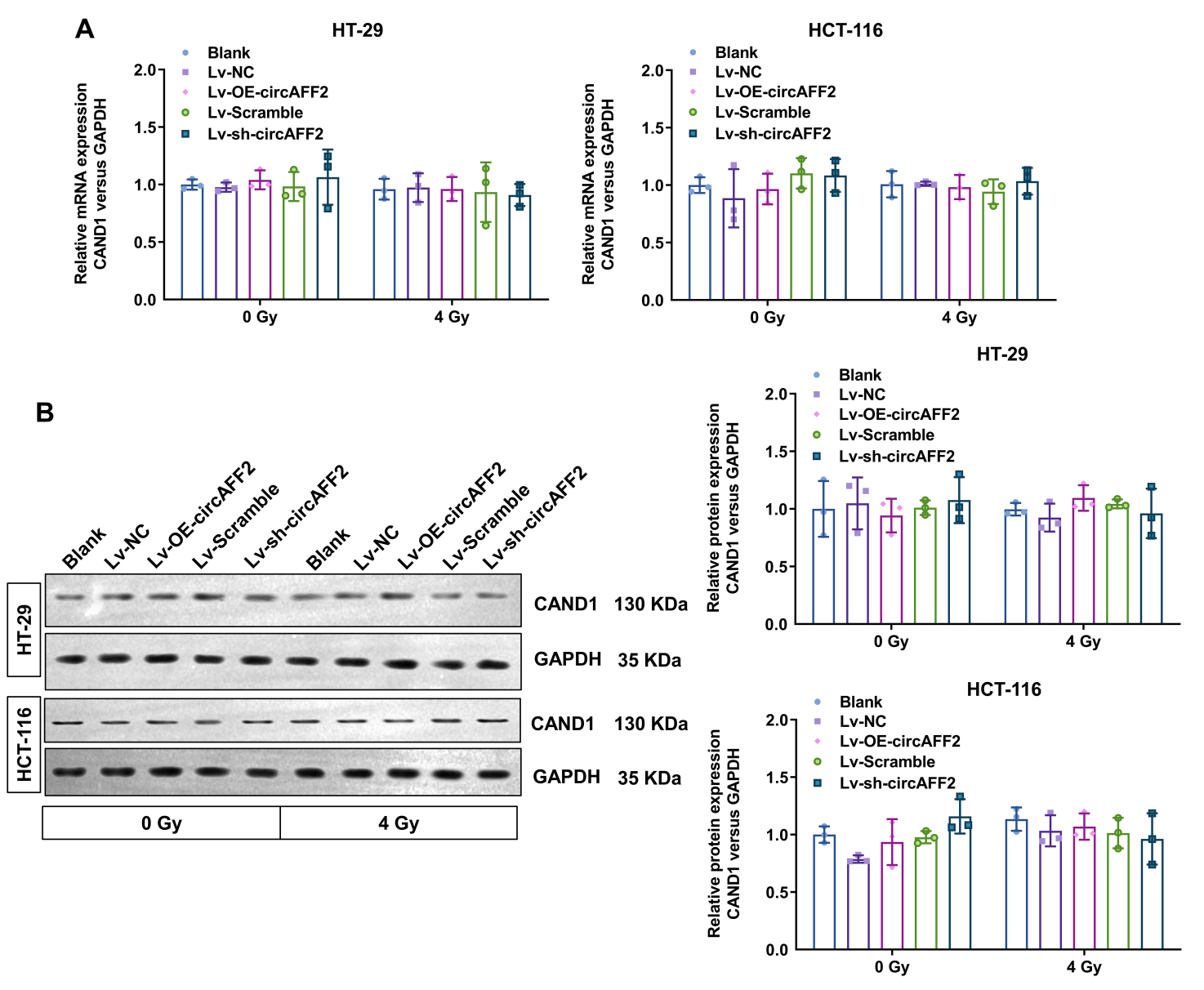
**

**Supplementary Figure 8** (A) qRT-PCR detection of CAND1 mRNA expression in CRC cell lines after up- or down-regulation of circAFF2; (B) Western blotting detection of CAND1 protein expression in CRC cell lines after up- or down-regulation of circAFF2. (****P* < 0.001, ***P* < 0.01, **P* < 0.05)

**
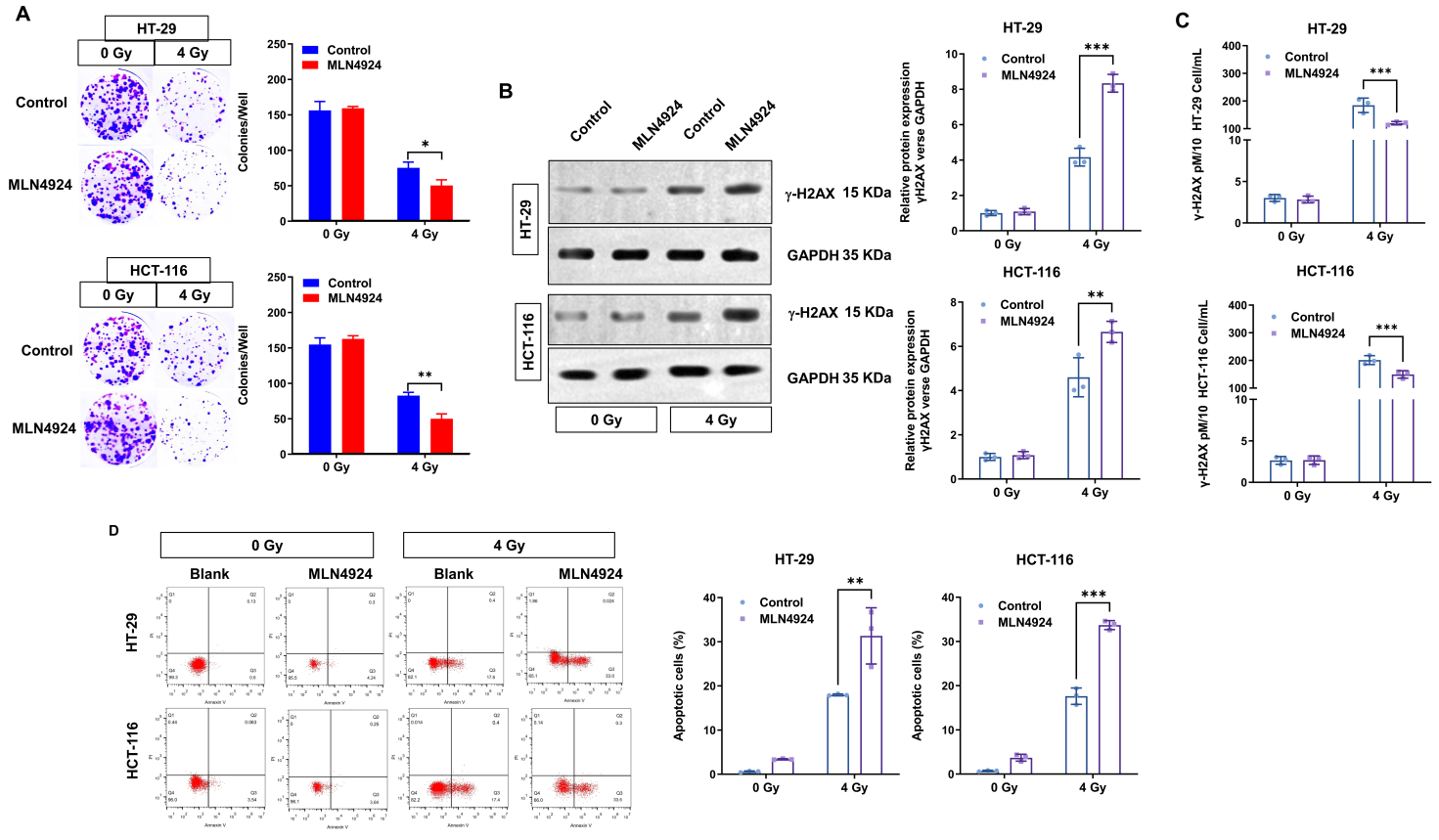
**

**Supplementary Figure 9** (A) Colony formation experiment to detect colony formation number after 4Gy irradiation in HT-29 and HCT-116 treated with MLN4924; (B) Western blotting to detect γ-H2AX after 4Gy irradiation in HT-29 and HCT-116 treated with MLN4924; (C) ELISA Assay to detect γ-H2AX after 4Gy irradiation in HT-29 and HCT-116 treated with MLN4924; (D) Flow cytometry detects apoptosis of cells after 4Gy irradiation in HT-29 and HCT-116 treated with MLN4924. ((****P* < 0.001, ***P* < 0.01, **P* < 0.05)
