# Supplementary material for: ALKBH5/YTHDF2‐mediated m6A modification of circAFF2 enhances radiosensitivity of colorectal cancer by inhibiting Cullin neddylation
Source: Clin Transl Med. 2023 Jun 28;13(7):e1318. doi: 10.1002/ctm2.1318 (PMC10307995; doi:10.1002/ctm2.1318)
Supplement: Supplementary file 2 — Supporting Information [file CTM2-13-e1318-s001.docx]

**Supplementary Materials and Methods**

**RNA interference experiment**

The shRNA sequences targeting the human hsa_circ_0000023, hsa_circ_0001947, hsa_circ_0000734, ALKBH5, YTHDF2 were designed by generbiol (Shanghai, China). The shRNA sequences were cloned into the lentiviral vector pLVX-sh1 (Colontech, CA, USA). Lentivirus (LV) (LV-shRNA-hsa_circ_0000023/hsa_circ_0001947/hsa_circ_0000734/ALKBH5/YTHDF2 and LV-shRNA-NC) amplification and packaging was conducted according to the lentiviral packaging protocol (Colontech, CA, USA). Briefly, the 293T packaging cell line was cotransfected with pLVX-sh1 vectors carrying shRNAs and pHelper plasmids. The next day, medium was replaced with fresh DMEM and culture was continued for 24 h at 37°C. The viral supernatant was then collected, filtered, concentrated and stored in small aliquots at −80°C for titration and cell infection.

SiRNA oligonucleotides targeting the protein coding-sequence of human ALKBH5 and CAND1 were synthesized as purified and annealed duplexes by generbiol (Shanghai, China). To initiate transfection, 50 μL of OPTI-MEM medium was mixed with 10 μL of oligofectamine reagent for 10 minutes. Next, the mixture was incubated with a solution containing 10 μL of siRNA and 200 μL of OPTI-MEM medium for 20 minutes at room temperature. An additional 130 μL of OPTI-MEM medium was then added to the mixture, which was subsequently added to HCT-116 and HT-29 cells cultured on a 6-well plate at 30%–40% confluency. The cells were transfected three times every 24 hours, and lysed 24 hr after the last transfection.

**In situ hybridization (ISH) and Immunohistochemistry (IHC)**

The tissues were saturated in prehybridization solution. After incubation for one hour at 37 °C, hybridization solution containing the circAFF2 probe (5’-AGTGCATCACCTTTGTTTGTTTCACTTGTTTGGAGGATGG-3’) was applied to tissues and incubated at 4 °C overnight. After incubation with circAFF2 probe, tissues were washed with 2 × SSC for 5 min at 37 °C, 1×SSC 5 min at 37 °C and 0.5 × SSC for 10 min at room temperature, followed by blocking with 5% BSA for 30 minutes at 25°C. The tissues were then incubated with mouse anti-biotin-labeled peroxidase (#7075, Cell Signaling Technology) at 37 °C for 40 min. After that, the tissues were stained with 0.05% 3,3‑diaminobenzidine for 1 minute, counterstained with 10% hematoxylin for 3 minutes, dehydrated with graded ethanol, and observed under microscope.

Tissues were incubated with the monoclonal ALKBH5 monoclonal antibody (1:200, Proteintech, USA) at 4 °C, followed by incubation with IgG (ab150113, Abcam, USA) for 2h at room temperature.

A scoring system was used to assess the degree of expression based on the percentage of positively labelled cells (0, 0%; 1, <10%; 2, 10–50% and 3, >50%) and staining intensity (0, no staining; 1, weak staining; 2, moderate staining and 3, strong staining).

**RNA preparation and real-time qPCR**

ABI Vii7 system (Applied Biosystems, USA) was used for qRT-PCR with the following setup: 95°C for thirty seconds, followed by 95°C for thirty seconds, 60°C for thirty seconds, 72°C for thirty seconds, for initial denaturation, denaturation, annealing, and extension, respectively, with a total of 40 cycles. SYBR green reagent (Thermo Fisher, Waltham, MA, USA) is used to stain DNA. Human glyceraldehyde-3-phosphate dehydrogenase (GAPDH) was used as the internal control. Comparative CT method (2^-ΔΔ^Ct) was adopted to calculate relative gene expression. The primers are shown in Supplementary Table 2.

**Immunoblotting**

Anti-ALKBH5 antibody (1:1000;16837-1-AP, Proteintech, Wuhan), anti-γ-H2AX antibody (1:2000;Ab11174, Abcam, USA), antibody against NEDD8 (1:1500; Ab139468, USA), anti-CAND1 antibody (1:1000;A14287, ABclonal, China), anti-CKB antibody (1:2000; A12632, ABclonal), anti-KNG1 antibody (1:800; A1670, ABclonal), anti-RRAS2 antibody (1:2000; A7076, ABclonal), anti-Cullin1 antibody (1:1500;Ab75817, Abcam, USA), HRP labeled anti-GAPDH (1:8000, AC035, ABclonal). The band was visualized using the enhanced chemiluminescence kit (Thermo Fisher, USA) and exposed by Tanon 4600 Automatic Chemiluminescence Imaging Analysis System (Tanon, China).

**Colony formation assay**

Suspension of transfected cells was inoculated into 6-well plates. After inoculation, irradiation with different doses of 6MV X-ray was applied to cells. After 10-14 days culture, methanol was used for colony fixation and 0.5% crystal violet (in 20% methanol) for colony staining and counting.

**Apoptosis detection**

Apoptotic cell death was assessed by AnnexinV/PI apoptosis kit (Beyotime, China). After incubation for 48 hours, about 1×10^5^ cells were stained with AnnexinV/PI avoiding light for 5 min and then analyzed in two-color flow cytometry. After treatment with the two probes, early apoptotic cells fluoresced green, and late apoptotic cells fluoresced red and green. Living cells have little or no fluorescence.

**γ-H2AX quantification**

The level of γ-H2AX in cells were evaluated with a γ-H2AX (Human Gamma H2AX ELISA Kit; C 4418-096-K, Bio-techne). The γ-H2AX antibody was used according to the ELISA principle. γ-H2AX levels were quantified at 450 nm with standard curve calculation.

**RNase R Resistance assay**

Total RNA (2 μg) was isolated and incubated at 37°C for 1 h with the presence or absence of RNase R (5 U/μg, R7092L, Beyotime, China). The remaining amount of circAFF2 was determined by qRT-PCR.

**Nuclear-cytoplasmic fractionation**

Total RNA was isolated from cells by utilizing TRIzol reagent (Takara, Dalian, China). Nuclear and cytoplasmic RNA was prepared from CRC HCT-116 and HT-29 cells using PARIS™Kit (Thermo Fisher, Austin, Texas, USA).

**Fluorescence in situ hybridization**

Cells (1 × 10^5^) were fixed in 4% paraformaldehyde (PFA) for 10 minutes and then rinsed with phosphate buffered saline (PBS). Then the PBS was precooled for fifteen minutes with 0.5% Triton X-100 at 4℃. A PE labeled probe (Geneseed, Guangzhou, China) for hsa_circ 0001947 was synthesized. The nuclei were incubated with FISH probe at 37°C for 16 hin hybridization buffer (Geneseed, Guangzhou, China) and incubated with DAPI. Images were acquired with the fluorescence microscope (Olympus CX53) (Tokyo, Japan).

**RNA m6A methylation quantification**

The level m6A methylation in total RNA was measured by m6A RNA Methylation Assay Kit (Abcam, Cambridge, MA USA).

**Dot Blot**

The total RNA (400ng) was doubled diluted and labeled on a nylon membrane (GE Healthcare). UV cross-link was applied to the membrane before incubation with m6A antibody and HRP conjugated anti-rabbit immunoglobulin G. The dot was developed by the enhanced chemiluminescence kit (Thermo Fisher, USA) and exposed by Tanon 4600 Automatic Chemiluminescence Imaging Analysis System (Tanon, Shanghai, China). Finally, membranes were labeled with the same 400ng RNA, incubated with 0.02% methylene blue (0.3M sodium acetate) for two hours for staining followed by washing with ribonuclease-free water for five hours.

**RNA immunoprecipitation (RIP)**

The interaction between RBP CAND1 and circAFF2 was detected according to the instruction of the RNA immunoprecipitation kit. In simple terms, 1×10^7^ cells were collected and lysed with lysis buffer. Cell lysates were then incubated with magnetic beads pre-coated with anti-IgG (CST, Beverly, MA, USA) or anti-CAND1 antibody(1:100; A14287, ABclonal, Shanghai, China). The coprecipitated RNA was detected by qRT-PCR with corresponding primers.

**Me-RIP-qPCR**

Me-RIP was performed using RNA immunoprecipitation kit following the manufacturer's protocol (17-10499, Merck Millipore). The cells were lysed with 1 mL RIP lysate for 10 min, then 100μL RIP lysate was taken and stored at −80℃.The anti-m6A antibody (1:50) or normal rabbit IgG (1:50) was mixed with protein A/G microbeads at 4℃ for 2 h, and then incubated with lysate 450μL at 4℃ for 2 h. Finally, qRT-PCR was performed for RNA analysis as described above. Sequences of primers for Me-RIP-qPCR were showed in Supplementary Table 3.

**Luciferase report assay**

We firstly constructed six luciferase reporter plasmids by inserting partial circAFF2 sequences with wild-type or mutated m6A sites (M1-M5). For luciferase assays, ALKHB5 over-expression or knockdown HT-29 and HCT-116 colon cancer cells were plated in 24-well plates (1 × 10^5^ cells/well). The cells were then transfected with the plasmid with Lipofectamine 3000 (Thermo Fisher, Waltham, MA, USA). Twenty-four hours following transfection, we measured the luciferase activity in the harvested cells using the Dual-Luciferase Reporter Assay System (Promega, San Luis Obispo, CA, USA). Renilla luciferase activity was used for normalization of relative activity of luciferase.

**Co- immunoprecipitation (Co-IP) assay**

Co-IP was determined using a Co-immunoprecipitation kit (Millipore, USA) in accordance with the manufacturer's procedures. In simple terms, cells were collected and lysed, and then mixed with 10μg or specific CAND1 antibody (1:50; A14287, AB Clone, Shanghai, China) coated with beads, rotated overnight at 4°C. Bead binding protein was released and detected by Western blot.

**RNA pull-down analysis and mass spectrometry**

The binding of circAFF2 with RNA binding protein was examined using the Magnetic RNA-protein pull-down Kit (ThermoFisher Scientific, USA). RiboBio (Guangzhou, China) synthesized a biotin-labeled probe targeting the circAFF2 junction site, using oligomer probes as controls. The cells were lysed and incubated with a biotin-labeled circAFF2 probe. Subsequently, cell lysates were treated with streptavidin agarose beads at room temperature. Finally, the interacting proteins were identified by mass spectrometry and Western blot.

**Animal experiments**

We performed all the animal experiments strictly following the Guide for the Care and Use of Laboratory Animals (NIH publication 80–23, revised 1996). The animal experiments were performed with approval by The Third Affiliated Hospital of Soochow University. Genetic modified HCT-116 cells were constructed by lentivirus (Len) transfection of Len-OE-circAFF2, Len-sh-circAFF2, Len-sh-ALKBH5, Len-sh-YTHDF2, Len-sh-ALKBH5 + Len-OE-circAFF2 and Len-sh-YTHDF2 + Len-sh-cirAFF2. Cell suspensions with a concentration of 1×10^7^ were prepared when their growth rate is logarithmic. Tumor grafting murine model was developed via subcutaneous injection of tumor cells to female BALB/c nude mice (4-5-week-old). 10 days following tumor grafting (tumor volume reached about 200 mm^3^), animals were received 10 Gy irradiation for one time. Tumor volume was measured and recorded by vernier calipers every 5 days after irradiation. 30 days after irradiation, mice were anesthetized, and tissues were collected.
