# Supplementary material for: ALKBH5/YTHDF2‐mediated m6A modification of circAFF2 enhances radiosensitivity of colorectal cancer by inhibiting Cullin neddylation
Source: Clin Transl Med. 2023 Jun 28;13(7):e1318. doi: 10.1002/ctm2.1318 (PMC10307995; doi:10.1002/ctm2.1318)
Supplement: Supplementary file 3 — Supporting Information [file CTM2-13-e1318-s002.docx]

**Supplementary table 1 Sequences of siRNA and shRNA used in the study**

| Oligo ids | Sequence |
| --- | --- |
| ALKBH5-siRNA-1 | CCCATCGTGTCCGTGTCCTTCTTTA |
| ALKBH5 siRNA-2 | GAAGCGTGACTGTGCTCAGTGGATA |
| ALKBH5 siRNA-3 | CGGTTGGAAACAAAGTCCCTGAGCA |
| siCAND1-1 | GGGCAACCTTCCTGAATAT |
| siCAND1-2 | GCCCTTGATATTCTAATAA |
| siCAND1-3 | GGGCATCATATTGACTTAA |
| has_circ_0000023-shRNA | GGATCCAACTATCAAATGAGAAAGATTTCAAGAGAATCTTTCTCATTTGATAGTTTTTTTTGAATTC |
| has_circ_0001947-shRNA | GGATCCAACAAGTGAAACAAACAAAGTTCAAGAGACTTTGTTTGTTTCACTTGTTTTTTTTGAATTC |
| has_circ_0000734-shRNA | GGATCCTATAATGAAGGCCATCATGCTTCAAGAGAGCATGATGGCCTTCATTATATTTTTTGAATTC |
| ALKBH5 shRNA-1 | GGATCCCCCATCGTGTCCGTGTCCTTCTTTATTCAAGAGATAAAGAAGGACACGGACACGATGGGTTTTTTGAATTC |
| ALKBH5 shRNA-2 | GGATCCGAAGCGTGACTGTGCTCAGTGGATATTCAAGAGATATCCACTGAGCACAGTCACGCTTCTTTTTTGAATTC |
| ALKBH5 shRNA-3 | GGATCCCGGTTGGAAACAAAGTCCCTGAGCATTCAAGAGATGCTCAGGGACTTTGTTTCCAACCGTTTTTTGAATTC |
| YTHDF2 shRNA-1 | CCGGTACTGATTAAGTCAGGATTAACTCGAGTTAATCCTGACTTAATCAGTATTTTTG |
| YTHDF2 shRNA-2 | CCGGGCTACTCTGAGGACGATATTCCTCGAGGAATATCGTCCTCAGAGTAGCTTTTTG |

**Supplementary table 2 Sequences of primers used in the study**

| **Gene** | **Forward primer (5’-3’)** | **Reverse primer (5’-3’)** |
| --- | --- | --- |
| CircAFF2 | CTTGGATGGAAAACCCAGTG | AGTTTCCAAGCGTGTTCTGG |
| METTL14 | CTACCCATCCTCACTGTCAGTC | GGATGTTCCTGTTTGACCTGAGG |
| RBM15 | TCCCACCTTGTGAGTTCTCC | GTCAGCGCCAAGTTTTCTCT |
| WTAP | CTTCCCAAGAAGGTTCGATTGA | TCAGACTCTCTTAGGCCAGTTAC |
| VIRMA | AATCCTGTGGGAAGATCAGC | ACACGTAAGGCAGTGGTAAG |
| FTO | CCAGAACCTGAGGAGAGAATGG | CGATGTCTGTGAGGTCAAACGG |
| ALKBH5 | CCAGCTATGCTTCAGATCGCCT | GGTTCTCTTCCTTGTCCATCTCC |
| GAPDH | TGACTTCAACAGCGACACCCA | CACCCTGTTGCTGTAGCCAAA |

**Supplementary table 3 Sequences of primers** **for Me-RIP-qPCR used in the study**

| **Gene** | **Forward primer (5’-3’)** | **Reverse primer (5’-3’)** |
| --- | --- | --- |
| hsa_circ_0000228 | TGTGGGGTGTGAGAACTTGA | TCAAGCAGACAGTAGCCAAA |
| hsa_circ_0001277 | CATCGCGGACACAAACATCA | AGCTTCTTCATCAGGACCCA |
| hsa_circ_0005310 | TCACCACCTCCAGACTATCC | TTGTTCTCCTGCCAGTTCAG |
| hsa_circ_0000023 | CGAAGACCTGCTCTACCACT | TGGCTGGCAGAAAGGGTAG |
| hsa_circ_0003322 | GCAGGCTTGGAGTTGAACTG | ACCTTCCACAGTCGAAATCTCT |
| hsa_circ_0001947 | CTTGGATGGAAAACCCAGTG | AGTTTCCAAGCGTGTTCTGG |
| hsa_circ_0000734 | AGACGGAAGGAGAGTAGTTATCT | GATAACGCGGCGGACTATTC |
| hsa_circ_0005736 | TGCTACTTGATTAGGCTAAGACA | CCGGGAACAAGGCAGGATAT |
| hsa_circ_0000690 | GCGAGCGAGTTTGTGAAAAT | TCGATGTTGCCACTGTCAGT |
| hsa_circ_0008518 | CACCATTTACCCAAGCCAGAG | GTAAGGAGGTGGGAAGCTGT |
| hsa_circ_0008498 | TGCCTTTGAGACTCCCGAAA | GGTCTTCATGAACTCGGTCC |
| hsa_circ_0004568 | GTACCCCAGTTTCCACCCAG | TGCCATAGTAACAGATCATTCCA |
| hsa_circ_0008967 | TCCGTTGTCCTGATGGCATA | CCCCACTAGCACCATGTAGA |
| hsa_circ_0042231 | CCTGTCTCCACACCGAAGAG | TGTGTCGCTGGATGGTGAG |
| hsa_circ_0000271 | TGAAGCTGGCCCTGTGTG | CCTCGATGATACAGTCCACCA |
| hsa_circ_0003448 | TCCTTTGTGAAGCCCAAGGA | AGAGCCAATGATACCTTCCTGT |

**Supplementary table 4 The binding protein of circAFF2 was identified by RNA Pull Down & LC-MS**

| Protein Group ID | # Proteins | # Unique Peptides | # Peptides | # PSMs | Group Description |
| --- | --- | --- | --- | --- | --- |
| 244 | 14 | 33 | 33 | 54 | [Master Protein] cDNA, FLJ95666, highly similar to Homo sapiens albumin (ALB), mRNA OS=Homo sapiens OX=9606 PE=2 SV=1 |
| 379 | 7 | 28 | 32 | 54 | [Master Protein] Cytokeratin-1 OS=Homo sapiens OX=9606 GN=KRT1 PE=3 SV=1 |
| 357 | 15 | 26 | 35 | 47 | [Master Protein] Keratin, type II cytoskeletal 2 epidermal OS=Homo sapiens OX=9606 GN=KRT2 PE=1 SV=2 |
| 667 | 5 | 26 | 26 | 29 | [Master Protein] Neuroblast differentiation-associated protein AHNAK OS=Homo sapiens OX=9606 GN=AHNAK PE=1 SV=2 |
| 813 | 3 | 23 | 23 | 30 | [Master Protein] IgGFc-binding protein OS=Homo sapiens OX=9606 GN=FCGBP PE=1 SV=3 |
| 574 | 37 | 22 | 22 | 32 | [Master Protein] Sodium/potassium-transporting ATPase subunit alpha (Fragment) OS=Homo sapiens OX=9606 PE=2 SV=1 |
| 769 | 8 | 22 | 22 | 25 | [Master Protein] Clathrin heavy chain OS=Homo sapiens OX=9606 GN=CLTC PE=1 SV=1 |
| 706 | 27 | 21 | 21 | 25 | [Master Protein] Annexin A2 OS=Homo sapiens OX=9606 GN=ANXA2 PE=1 SV=2 |
| 847 | 2 | 20 | 21 | 41 | [Master Protein] Keratin, type I cytoskeletal 9 OS=Homo sapiens OX=9606 GN=KRT9 PE=1 SV=3 |
| 257 | 6 | 19 | 19 | 25 | [Master Protein] Protein disulfide-isomerase OS=Homo sapiens OX=9606 PE=2 SV=1 |
| 527 | 22 | 18 | 22 | 25 | [Master Protein] Keratin, type I cytoskeletal 10 OS=Homo sapiens OX=9606 GN=KRT10 PE=1 SV=6 |
| 120 | 6 | 16 | 17 | 20 | [Master Protein] Filamin-B OS=Homo sapiens OX=9606 GN=FLNB PE=1 SV=1 |
| 399 | 4 | 16 | 16 | 22 | [Master Protein] Calcium-activated chloride channel regulator 1 OS=Homo sapiens OX=9606 GN=CLCA1 PE=1 SV=3 |
| 474 | 29 | 15 | 15 | 19 | [Master Protein] Catenin (Cadherin-associated protein), alpha 1, 102kDa, isoform CRA_b OS=Homo sapiens OX=9606 GN=CTNNA1 PE=2 SV=1 |
| 507 | 14 | 13 | 13 | 13 | [Master Protein] Beta-1 metal-binding globulin OS=Homo sapiens OX=9606 GN=TF PE=1 SV=1 |
| 497 | 15 | 12 | 13 | 13 | [Master Protein] Filamin-A OS=Homo sapiens OX=9606 GN=FLNA PE=1 SV=4 |
| 27 | 15 | 12 | 12 | 15 | [Master Protein] Major vault protein OS=Homo sapiens OX=9606 PE=2 SV=1 |
| 109 | 13 | 12 | 12 | 15 | [Master Protein] Annexin (Fragment) OS=Homo sapiens OX=9606 GN=ANXA6 PE=2 SV=1 |
| 421 | 1 | 12 | 12 | 13 | [Master Protein] Hornerin OS=Homo sapiens OX=9606 GN=HRNR PE=1 SV=2 |
| 169 | 12 | 11 | 12 | 13 | [Master Protein] Epididymis luminal protein 35 OS=Homo sapiens OX=9606 GN=HEL-S-125m PE=2 SV=1 |
| 846 | 11 | 11 | 11 | 16 | [Master Protein] Integrin beta-1 OS=Homo sapiens OX=9606 GN=ITGB1 PE=1 SV=2 |
| 721 | 1 | 11 | 11 | 13 | [Master Protein] Polymeric immunoglobulin receptor OS=Homo sapiens OX=9606 GN=PIGR PE=1 SV=4 |
| 639 | 7 | 11 | 11 | 12 | [Master Protein] Desmoplakin OS=Homo sapiens OX=9606 GN=DSP PE=1 SV=3 |
| 854 | 38 | 10 | 20 | 23 | [Master Protein] Keratin, type II cytoskeletal 5 OS=Homo sapiens OX=9606 GN=KRT5 PE=1 SV=3 |
| 755 | 8 | 10 | 10 | 12 | [Master Protein] ATP synthase subunit alpha, mitochondrial OS=Homo sapiens OX=9606 GN=ATP5F1A PE=1 SV=1 |
| 638 | 8 | 10 | 10 | 10 | [Master Protein] Dolichyl-diphosphooligosaccharide--protein glycosyltransferase subunit 1 (Fragment) OS=Homo sapiens OX=9606 PE=2 SV=1 |
| 802 | 4 | 9 | 9 | 12 | [Master Protein] Integrin alpha-6 OS=Homo sapiens OX=9606 GN=ITGA6 PE=1 SV=5 |
| 326 | 11 | 9 | 9 | 12 | [Master Protein] Catenin delta-1 OS=Homo sapiens OX=9606 GN=CTNND1 PE=1 SV=2 |
| 761 | 7 | 9 | 9 | 11 | [Master Protein] Triosephosphate isomerase OS=Homo sapiens OX=9606 GN=HEL-S-49 PE=2 SV=1 |
| 333 | 8 | 9 | 9 | 10 | [Master Protein] Transitional endoplasmic reticulum ATPase OS=Homo sapiens OX=9606 GN=VCP PE=1 SV=4 |
| 495 | 10 | 9 | 9 | 9 | [Master Protein] Adenosylhomocysteinase 3 (Fragment) OS=Homo sapiens OX=9606 GN=AHCYL2 PE=1 SV=1 |
| 327 | 6 | 9 | 9 | 9 | [Master Protein] Spectrin beta chain OS=Homo sapiens OX=9606 GN=SPTBN1 PE=2 SV=1 |
| 404 | 38 | 8 | 12 | 15 | [Master Protein] Keratin, type I cytoskeletal 14 OS=Homo sapiens OX=9606 GN=KRT14 PE=1 SV=4 |
| 520 | 9 | 8 | 11 | 12 | [Master Protein] Non-muscle myosin heavy chain 9 OS=Homo sapiens OX=9606 GN=MYH9 PE=2 SV=1 |
| 314 | 40 | 8 | 10 | 11 | [Master Protein] Vimentin OS=Homo sapiens OX=9606 GN=HEL113 PE=2 SV=1 |
| 715 | 3 | 8 | 9 | 10 | [Master Protein] 78 kDa glucose-regulated protein OS=Homo sapiens OX=9606 GN=HEL-S-89n PE=2 SV=1 |
| 778 | 15 | 8 | 8 | 11 | [Master Protein] Elongation factor 2 OS=Homo sapiens OX=9606 GN=EEF2 PE=1 SV=4 |
| 814 | 1 | 8 | 8 | 11 | [Master Protein] Epididymis secretory sperm binding protein OS=Homo sapiens OX=9606 PE=2 SV=1 |
| 562 | 9 | 8 | 8 | 10 | [Master Protein] Annexin A4 OS=Homo sapiens OX=9606 GN=ANXA4 PE=1 SV=4 |
| 819 | 6 | 8 | 8 | 10 | [Master Protein] Unconventional myosin-Id OS=Homo sapiens OX=9606 GN=MYO1D PE=1 SV=2 |
| 264 | 3 | 8 | 8 | 9 | [Master Protein] Protein disulfide-isomerase A4 OS=Homo sapiens OX=9606 GN=ERP70 PE=2 SV=1 |
| 676 | 13 | 8 | 8 | 9 | [Master Protein] Actin-depolymerizing factor OS=Homo sapiens OX=9606 PE=2 SV=1 |
| 204 | 5 | 8 | 8 | 9 | [Master Protein] cDNA FLJ53009, highly similar to Calreticulin OS=Homo sapiens OX=9606 PE=2 SV=1 |
| 190 | 9 | 8 | 8 | 8 | [Master Protein] Integrin beta OS=Homo sapiens OX=9606 GN=ITGB4 PE=3 SV=1 |
| 727 | 24 | 8 | 8 | 8 | [Master Protein] Pyruvate kinase OS=Homo sapiens OX=9606 GN=PKM PE=1 SV=1 |
| 833 | 43 | 7 | 14 | 17 | [Master Protein] Keratin, type II cytoskeletal 8 OS=Homo sapiens OX=9606 GN=KRT8 PE=1 SV=7 |
| 771 | 12 | 7 | 8 | 8 | [Master Protein] Actinin alpha 4 isoform 1 (Fragment) OS=Homo sapiens OX=9606 GN=ACTN4 PE=2 SV=1 |
| 597 | 2 | 7 | 7 | 14 | [Master Protein] Cell surface A33 antigen OS=Homo sapiens OX=9606 GN=GPA33 PE=1 SV=1 |
| 852 | 4 | 7 | 7 | 10 | [Master Protein] Galectin OS=Homo sapiens OX=9606 GN=LGALS4 PE=2 SV=1 |
| 97 | 15 | 7 | 7 | 9 | [Master Protein] 40S ribosomal protein S3a OS=Homo sapiens OX=9606 GN=RPS3A PE=2 SV=1 |
| 386 | 7 | 7 | 7 | 9 | [Master Protein] Glyceraldehyde-3-phosphate dehydrogenase OS=Homo sapiens OX=9606 PE=2 SV=1 |
| 384 | 7 | 7 | 7 | 8 | [Master Protein] cDNA FLJ57023, highly similar to Homo sapiens collagen, type VI, alpha 3 (COL6A3), transcript variant 3, mRNA OS=Homo sapiens OX=9606 PE=2 SV=1 |
| 536 | 9 | 7 | 7 | 7 | [Master Protein] Epididymis secretory sperm binding protein OS=Homo sapiens OX=9606 PE=2 SV=1 |
| 661 | 10 | 7 | 7 | 7 | [Master Protein] Solute carrier family 12 (Sodium/potassium/chloride transporters), member 2, isoform CRA_a OS=Homo sapiens OX=9606 GN=SLC12A2 PE=1 SV=1 |
| 553 | 44 | 6 | 17 | 21 | [Master Protein] HCG2039812, isoform CRA_b (Fragment) OS=Homo sapiens OX=9606 GN=KRT6A PE=2 SV=1 |
| 235 | 12 | 6 | 9 | 11 | [Master Protein] Myosin, heavy polypeptide 11, smooth muscle, isoform CRA_b OS=Homo sapiens OX=9606 GN=MYH11 PE=3 SV=1 |
| 672 | 29 | 6 | 8 | 9 | [Master Protein] Epididymis luminal protein 33 OS=Homo sapiens OX=9606 GN=HEL-S-72p PE=2 SV=1 |
| 16 | 17 | 6 | 7 | 10 | [Master Protein] Fructose-bisphosphate aldolase OS=Homo sapiens OX=9606 GN=HEL-S-87p PE=2 SV=1 |
| 809 | 11 | 6 | 6 | 13 | [Master Protein] Alpha-enolase OS=Homo sapiens OX=9606 GN=ENO1 PE=1 SV=2 |
| 565 | 6 | 6 | 6 | 8 | [Master Protein] Galectin (Fragment) OS=Homo sapiens OX=9606 PE=2 SV=1 |
| 649 | 18 | 6 | 6 | 7 | [Master Protein] Peripheral plasma membrane protein CASK OS=Homo sapiens OX=9606 GN=CASK PE=1 SV=1 |
| 528 | 8 | 6 | 6 | 7 | [Master Protein] Testicular tissue protein Li 96 OS=Homo sapiens OX=9606 PE=2 SV=1 |
| 518 | 10 | 6 | 6 | 7 | [Master Protein] RPS4X protein (Fragment) OS=Homo sapiens OX=9606 GN=RPS4X PE=2 SV=2 |
| 471 | 19 | 6 | 6 | 6 | [Master Protein] Protein disulfide-isomerase OS=Homo sapiens OX=9606 GN=P4HB PE=1 SV=2 |
| 718 | 8 | 6 | 6 | 6 | [Master Protein] Junction plakoglobin (Fragment) OS=Homo sapiens OX=9606 GN=JUP PE=2 SV=1 |
| 492 | 2 | 6 | 6 | 6 | [Master Protein] Talin-1 OS=Homo sapiens OX=9606 GN=TLN1 PE=1 SV=3 |
| 262 | 8 | 6 | 6 | 6 | [Master Protein] Dopamine receptor interacting protein 4 OS=Homo sapiens OX=9606 GN=DRIP4 PE=2 SV=1 |
| 840 | 24 | 6 | 6 | 6 | [Master Protein] Receptor of activated protein C kinase 1 OS=Homo sapiens OX=9606 GN=RACK1 PE=1 SV=3 |
| 812 | 9 | 5 | 8 | 10 | [Master Protein] Myosin-14 OS=Homo sapiens OX=9606 GN=MYH14 PE=1 SV=2 |
| 444 | 31 | 5 | 7 | 8 | [Master Protein] Heat shock 70 kDa protein 1B OS=Homo sapiens OX=9606 GN=HSPA1B PE=1 SV=1 |
| 741 | 42 | 5 | 6 | 8 | [Master Protein] Uncharacterized protein OS=Homo sapiens OX=9606 GN=DKFZp686K03196 PE=1 SV=1 |
| 359 | 11 | 5 | 6 | 6 | [Master Protein] Protein transport protein Sec23B OS=Homo sapiens OX=9606 GN=SEC23B PE=1 SV=2 |
| 22 | 20 | 5 | 6 | 6 | [Master Protein] Epididymis luminal protein 4 OS=Homo sapiens OX=9606 GN=YWHAZ PE=2 SV=1 |
| 33 | 75 | 5 | 5 | 12 | [Master Protein] Elongation factor 1-alpha (Fragment) OS=Homo sapiens OX=9606 PE=2 SV=1 |
| 252 | 13 | 5 | 5 | 10 | [Master Protein] Carbonic anhydrase (Fragment) OS=Homo sapiens OX=9606 GN=CA1 PE=1 SV=1 |
| 779 | 3 | 5 | 5 | 8 | [Master Protein] Epithelial cell adhesion molecule OS=Homo sapiens OX=9606 GN=EPCAM PE=1 SV=2 |
| 114 | 6 | 5 | 5 | 6 | [Master Protein] ATP synthase subunit beta (Fragment) OS=Homo sapiens OX=9606 GN=ATP5B PE=2 SV=1 |
| 606 | 4 | 5 | 5 | 6 | [Master Protein] Annexin OS=Homo sapiens OX=9606 PE=2 SV=1 |
| 653 | 7 | 5 | 5 | 6 | [Master Protein] Phosphoglycerate kinase OS=Homo sapiens OX=9606 GN=HEL-S-68p PE=2 SV=1 |
| 640 | 19 | 5 | 5 | 6 | [Master Protein] cDNA, FLJ92803, highly similar to Homo sapiens hydroxysteroid (17-beta) dehydrogenase 4 (HSD17B4), mRNA OS=Homo sapiens OX=9606 PE=2 SV=1 |
| 85 | 27 | 5 | 5 | 6 | [Master Protein] Annexin OS=Homo sapiens OX=9606 PE=3 SV=1 |
| 430 | 13 | 5 | 5 | 6 | [Master Protein] 60S ribosomal protein L6 OS=Homo sapiens OX=9606 GN=RPL6 PE=3 SV=1 |
| 478 | 10 | 5 | 5 | 5 | [Master Protein] Isocitrate dehydrogenase [NADP] cytoplasmic OS=Homo sapiens OX=9606 GN=IDH1 PE=1 SV=2 |
| 122 | 15 | 5 | 5 | 5 | [Master Protein] Plasma membrane calcium-transporting ATPase 1 OS=Homo sapiens OX=9606 GN=ATP2B1 PE=1 SV=4 |
| 776 | 5 | 5 | 5 | 5 | [Master Protein] Prostaglandin F2 receptor negative regulator OS=Homo sapiens OX=9606 GN=PTGFRN PE=1 SV=2 |
| 289 | 5 | 5 | 5 | 5 | [Master Protein] Junctional adhesion molecule A OS=Homo sapiens OX=9606 GN=F11R PE=1 SV=1 |
| 849 | 4 | 5 | 5 | 5 | [Master Protein] Cytoskeleton-associated protein 4, isoform CRA_c OS=Homo sapiens OX=9606 GN=CKAP4 PE=4 SV=1 |
| 355 | 15 | 5 | 5 | 5 | [Master Protein] Flotillin (Fragment) OS=Homo sapiens OX=9606 GN=FLOT1 PE=2 SV=1 |
| 816 | 12 | 5 | 5 | 5 | [Master Protein] Actin-like protein 3 OS=Homo sapiens OX=9606 GN=ACTR3 PE=3 SV=1 |
| 539 | 8 | 5 | 5 | 5 | [Master Protein] RRBP1 protein OS=Homo sapiens OX=9606 GN=RRBP1 PE=2 SV=1 |
| 508 | 5 | 5 | 5 | 5 | [Master Protein] cDNA FLJ75526, highly similar to Homo sapiens proliferation-associated 2G4, 38kDa (PA2G4), mRNA (Fragment) OS=Homo sapiens OX=9606 PE=2 SV=1 |
| 682 | 2 | 5 | 5 | 5 | [Master Protein] Calcium-transporting ATPase OS=Homo sapiens OX=9606 PE=2 SV=1 |
| 754 | 9 | 5 | 5 | 5 | [Master Protein] B-cell receptor-associated protein 31 OS=Homo sapiens OX=9606 GN=BCAP31 PE=1 SV=3 |
| 541 | 10 | 5 | 5 | 5 | [Master Protein] 60S ribosomal protein L4 OS=Homo sapiens OX=9606 GN=RPL4 PE=1 SV=5 |
| 161 | 7 | 5 | 5 | 5 | [Master Protein] Metavinculin OS=Homo sapiens OX=9606 GN=VCL PE=3 SV=1 |
| 554 | 6 | 5 | 5 | 5 | [Master Protein] cDNA FLJ55443, highly similar to Ras GTPase-activating-like protein IQGAP1 OS=Homo sapiens OX=9606 PE=2 SV=1 |
| 658 | 5 | 5 | 5 | 5 | [Master Protein] T-complex protein 1 subunit theta OS=Homo sapiens OX=9606 GN=CCT8 PE=1 SV=4 |
| 374 | 6 | 5 | 5 | 5 | [Master Protein] Integrin alpha-2 OS=Homo sapiens OX=9606 GN=ITGA2 PE=1 SV=1 |
| 456 | 19 | 4 | 8 | 9 | [Master Protein] Keratin, type I cytoskeletal 19 OS=Homo sapiens OX=9606 GN=KRT19 PE=1 SV=4 |
| 95 | 3 | 4 | 6 | 9 | [Master Protein] cDNA FLJ25298 fis, clone STM07683, highly similar to Protein Tro alpha1 H,myeloma OS=Homo sapiens OX=9606 PE=2 SV=1 |
| 266 | 18 | 4 | 4 | 6 | [Master Protein] Glucose-6-phosphate isomerase OS=Homo sapiens OX=9606 GN=GPI PE=1 SV=1 |
| 405 | 6 | 4 | 4 | 6 | [Master Protein] Annexin OS=Homo sapiens OX=9606 PE=2 SV=1 |
| 472 | 8 | 4 | 4 | 6 | [Master Protein] Hephaestin OS=Homo sapiens OX=9606 GN=HEPH PE=1 SV=1 |
| 660 | 8 | 4 | 4 | 5 | [Master Protein] Secretory carrier-associated membrane protein 2 OS=Homo sapiens OX=9606 GN=SCAMP2 PE=1 SV=2 |
| 644 | 11 | 4 | 4 | 5 | [Master Protein] Vesicle-fusing ATPase OS=Homo sapiens OX=9606 PE=2 SV=1 |
| 127 | 9 | 4 | 4 | 5 | [Master Protein] cDNA FLJ61290, highly similar to Neutral alpha-glucosidase AB OS=Homo sapiens OX=9606 PE=2 SV=1 |
| 104 | 8 | 4 | 4 | 5 | [Master Protein] Multidrug resistance associated protein OS=Homo sapiens OX=9606 GN=MRP3 PE=3 SV=1 |
| 144 | 15 | 4 | 4 | 5 | [Master Protein] Epididymis secretory sperm binding protein OS=Homo sapiens OX=9606 PE=2 SV=1 |
| 643 | 4 | 4 | 4 | 5 | [Master Protein] Suppressor of tumorigenicity 14 protein homolog OS=Homo sapiens OX=9606 PE=2 SV=1 |
| 744 | 2 | 4 | 4 | 5 | [Master Protein] Carnitine O-palmitoyltransferase OS=Homo sapiens OX=9606 PE=2 SV=1 |
| 76 | 6 | 4 | 4 | 5 | [Master Protein] Annexin OS=Homo sapiens OX=9606 GN=ANXA5 PE=1 SV=1 |
| 106 | 8 | 4 | 4 | 5 | [Master Protein] SEC31-like 1 (S. cerevisiae), isoform CRA_b OS=Homo sapiens OX=9606 GN=SEC31L1 PE=3 SV=1 |
| 803 | 5 | 4 | 4 | 5 | [Master Protein] Scavenger receptor cysteine-rich type 1 protein M130 OS=Homo sapiens OX=9606 GN=CD163 PE=1 SV=1 |
| 757 | 10 | 4 | 4 | 5 | [Master Protein] Calnexin OS=Homo sapiens OX=9606 GN=CANX PE=1 SV=2 |
| 188 | 5 | 4 | 4 | 4 | [Master Protein] Carboxylic ester hydrolase (Fragment) OS=Homo sapiens OX=9606 GN=CES2 PE=2 SV=1 |
| 176 | 17 | 4 | 4 | 4 | [Master Protein] TAP2 OS=Homo sapiens OX=9606 PE=3 SV=1 |
| 167 | 38 | 4 | 4 | 4 | [Master Protein] Tubulin alpha chain OS=Homo sapiens OX=9606 PE=2 SV=1 |
| 158 | 5 | 4 | 4 | 4 | [Master Protein] Unconventional myosin-Ic OS=Homo sapiens OX=9606 GN=MYO1C PE=1 SV=4 |
| 222 | 9 | 4 | 4 | 4 | [Master Protein] HCG2001986, isoform CRA_a OS=Homo sapiens OX=9606 GN=hCG_2001986 PE=4 SV=1 |
| 538 | 2 | 4 | 4 | 4 | [Master Protein] Sulfate transporter OS=Homo sapiens OX=9606 GN=SLC26A2 PE=1 SV=2 |
| 139 | 5 | 4 | 4 | 4 | [Master Protein] Sodium/potassium-transporting ATPase subunit beta-1 OS=Homo sapiens OX=9606 GN=ATP1B1 PE=1 SV=1 |
| 843 | 4 | 4 | 4 | 4 | [Master Protein] Carbonic anhydrase 2 OS=Homo sapiens OX=9606 GN=CA2 PE=1 SV=2 |
| 239 | 3 | 4 | 4 | 4 | [Master Protein] Phospholipid-transporting ATPase IC OS=Homo sapiens OX=9606 GN=ATP8B1 PE=1 SV=3 |
| 168 | 9 | 4 | 4 | 4 | [Master Protein] Plastin-1 OS=Homo sapiens OX=9606 GN=PLS1 PE=1 SV=2 |
| 90 | 14 | 4 | 4 | 4 | [Master Protein] cDNA FLJ56074, highly similar to 150 kDa oxygen-regulated protein (Orp150) OS=Homo sapiens OX=9606 PE=2 SV=1 |
| 756 | 5 | 4 | 4 | 4 | [Master Protein] Ubiquitin-activating enzyme E1 OS=Homo sapiens OX=9606 GN=UBE1 PE=2 SV=1 |
| 72 | 16 | 4 | 4 | 4 | [Master Protein] Heterogeneous nuclear ribonucleoprotein U OS=Homo sapiens OX=9606 GN=HNRNPU PE=1 SV=1 |
| 452 | 12 | 4 | 4 | 4 | [Master Protein] 60 kDa chaperonin OS=Homo sapiens OX=9606 GN=HSPD1 PE=2 SV=1 |
| 459 | 9 | 4 | 4 | 4 | [Master Protein] RAB11A, member RAS oncogene family, isoform CRA_a OS=Homo sapiens OX=9606 GN=RAB11A PE=4 SV=1 |
| 269 | 7 | 4 | 4 | 4 | [Master Protein] Annexin OS=Homo sapiens OX=9606 PE=2 SV=1 |
| 39 | 8 | 4 | 4 | 4 | [Master Protein] Methanethiol oxidase OS=Homo sapiens OX=9606 GN=SELENBP1 PE=1 SV=2 |
| 228 | 2 | 4 | 4 | 4 | [Master Protein] cDNA FLJ77398, highly similar to Homo sapiens UDP-glucose ceramide glucosyltransferase-like 1, transcript variant 2, mRNA OS=Homo sapiens OX=9606 PE=2 SV=1 |
| 83 | 15 | 4 | 4 | 4 | [Master Protein] 60S ribosomal protein L3 OS=Homo sapiens OX=9606 GN=RPL3 PE=1 SV=2 |
| 380 | 5 | 4 | 4 | 4 | [Master Protein] cDNA FLJ12728 fis, clone NT2RP2000040, highly similar to Protein FAM62A OS=Homo sapiens OX=9606 PE=2 SV=1 |
| 247 | 7 | 4 | 4 | 4 | [Master Protein] cDNA FLJ75774, highly similar to Homo sapiens lectin, mannose-binding 2 (LMAN2), mRNA OS=Homo sapiens OX=9606 PE=2 SV=1 |
| 784 | 7 | 4 | 4 | 4 | [Master Protein] 40S ribosomal protein S9 OS=Homo sapiens OX=9606 GN=RPS9 PE=1 SV=1 |
| 703 | 17 | 4 | 4 | 4 | [Master Protein] 40S ribosomal protein S3 OS=Homo sapiens OX=9606 GN=RPS3 PE=1 SV=2 |
| 801 | 6 | 4 | 4 | 4 | [Master Protein] 60S ribosomal protein L7 OS=Homo sapiens OX=9606 GN=RPL7 PE=1 SV=1 |
| 821 | 3 | 4 | 4 | 4 | [Master Protein] Carcinoembryonic antigen-related cell adhesion molecule 7 OS=Homo sapiens OX=9606 GN=CEACAM7 PE=1 SV=1 |
| 319 | 7 | 4 | 4 | 4 | [Master Protein] 60S ribosomal protein L14 OS=Homo sapiens OX=9606 GN=RPL14 PE=1 SV=4 |
| 502 | 3 | 4 | 4 | 4 | [Master Protein] 40S ribosomal protein S8 OS=Homo sapiens OX=9606 GN=RPS8 PE=2 SV=1 |
| 786 | 4 | 4 | 4 | 4 | [Master Protein] Endoplasmic reticulum-Golgi intermediate compartment protein 1 OS=Homo sapiens OX=9606 GN=ERGIC1 PE=1 SV=1 |
| 575 | 67 | 3 | 12 | 20 | [Master Protein] Beta actin variant (Fragment) OS=Homo sapiens OX=9606 PE=2 SV=1 |
| 611 | 14 | 3 | 7 | 7 | [Master Protein] Heat shock protein 90kDa alpha (Cytosolic), class B member 1, isoform CRA_a OS=Homo sapiens OX=9606 GN=HSP90AB1 PE=3 SV=1 |
| 646 | 15 | 3 | 6 | 6 | [Master Protein] Moesin OS=Homo sapiens OX=9606 GN=MSN PE=1 SV=3 |
| 842 | 266 | 3 | 5 | 6 | [Master Protein] cDNA FLJ41981 fis, clone SMINT2011888, highly similar to Protein Tro alpha1 H,myeloma OS=Homo sapiens OX=9606 PE=2 SV=1 |
| 704 | 28 | 3 | 5 | 5 | [Master Protein] IGK@ protein OS=Homo sapiens OX=9606 GN=IGK@ PE=1 SV=1 |
| 630 | 15 | 3 | 5 | 5 | [Master Protein] Keratin, type I cytoskeletal 18 OS=Homo sapiens OX=9606 GN=KRT18 PE=1 SV=2 |
| 303 | 10 | 3 | 5 | 5 | [Master Protein] ADP/ATP translocase 3 OS=Homo sapiens OX=9606 GN=SLC25A6 PE=1 SV=4 |
| 696 | 869 | 3 | 5 | 5 | [Master Protein] MHC class I antigen OS=Homo sapiens OX=9606 GN=HLA-A PE=3 SV=1 |
| 579 | 17 | 3 | 4 | 5 | [Master Protein] L-lactate dehydrogenase OS=Homo sapiens OX=9606 GN=LDHB PE=1 SV=1 |
| 563 | 14 | 3 | 4 | 4 | [Master Protein] Tyrosine 3-monooxygenase/tryptophan 5-monooxygenase activation protein, eta polypeptide, isoform CRA_b OS=Homo sapiens OX=9606 GN=YWHAH PE=3 SV=1 |
| 323 | 11 | 3 | 3 | 5 | [Master Protein] Prohibitin (Fragment) OS=Homo sapiens OX=9606 PE=2 SV=1 |
| 238 | 6 | 3 | 3 | 5 | [Master Protein] 60S ribosomal protein L8 (Fragment) OS=Homo sapiens OX=9606 GN=RPL8 PE=1 SV=1 |
| 524 | 9 | 3 | 3 | 4 | [Master Protein] 60S ribosomal protein L18 (Fragment) OS=Homo sapiens OX=9606 GN=RPL18 PE=1 SV=1 |
| 316 | 8 | 3 | 3 | 4 | [Master Protein] Creatine kinase (Fragment) OS=Homo sapiens OX=9606 GN=CKB PE=2 SV=1 |
| 608 | 8 | 3 | 3 | 4 | [Master Protein] Rab GDP dissociation inhibitor OS=Homo sapiens OX=9606 PE=2 SV=1 |
| 89 | 3 | 3 | 3 | 4 | [Master Protein] Chloride intracellular channel protein OS=Homo sapiens OX=9606 PE=3 SV=1 |
| 306 | 6 | 3 | 3 | 4 | [Master Protein] Flotillin OS=Homo sapiens OX=9606 GN=FLOT2 PE=2 SV=1 |
| 254 | 6 | 3 | 3 | 4 | [Master Protein] cDNA FLJ46245 fis, clone TESTI4020596, highly similar to Homo sapiens calpain 5 (CAPN5) OS=Homo sapiens OX=9606 PE=2 SV=1 |
| 142 | 4 | 3 | 3 | 4 | [Master Protein] SFPQ protein (Fragment) OS=Homo sapiens OX=9606 GN=SFPQ PE=2 SV=2 |
| 841 | 1 | 3 | 3 | 4 | [Master Protein] Syntaxin-binding protein 3 OS=Homo sapiens OX=9606 GN=STXBP3 PE=1 SV=2 |
| 589 | 6 | 3 | 3 | 3 | [Master Protein] Microsomal signal peptidase 25 kDa subunit OS=Homo sapiens OX=9606 GN=SPCS2 PE=1 SV=1 |
| 294 | 15 | 3 | 3 | 3 | [Master Protein] cDNA FLJ77680, highly similar to Homo sapiens protein phosphatase 2 (formerly 2A), regulatory subunit A (PR 65), alpha isoform (PPP2R1A), mRNA OS=Homo sapiens OX=9606 PE=2 SV=1 |
| 304 | 11 | 3 | 3 | 3 | [Master Protein] Glucosidase 2 subunit beta OS=Homo sapiens OX=9606 PE=2 SV=1 |
| 585 | 6 | 3 | 3 | 3 | [Master Protein] P-type Ca(2+) transporter (Fragment) OS=Homo sapiens OX=9606 GN=ATP2A2 PE=1 SV=1 |
| 229 | 12 | 3 | 3 | 3 | [Master Protein] Eukaryotic translation initiation factor 3 subunit C OS=Homo sapiens OX=9606 GN=EIF3C PE=2 SV=1 |
| 462 | 5 | 3 | 3 | 3 | [Master Protein] Endosome/lysosome-associated apoptosis and autophagy regulator 1 (Fragment) OS=Homo sapiens OX=9606 GN=ELAPOR1 PE=1 SV=1 |
| 285 | 15 | 3 | 3 | 3 | [Master Protein] Catenin beta-1 OS=Homo sapiens OX=9606 GN=CTNNB1 PE=1 SV=1 |
| 569 | 4 | 3 | 3 | 3 | [Master Protein] Na(+)/H(+) exchange regulatory cofactor NHE-RF1 OS=Homo sapiens OX=9606 GN=SLC9A3R1 PE=1 SV=4 |
| 451 | 9 | 3 | 3 | 3 | [Master Protein] Nodal modulator 2 OS=Homo sapiens OX=9606 GN=NOMO2 PE=1 SV=1 |
| 466 | 4 | 3 | 3 | 3 | [Master Protein] Apolipoprotein A1 (Fragment) OS=Homo sapiens OX=9606 PE=4 SV=1 |
| 594 | 4 | 3 | 3 | 3 | [Master Protein] Aldo-keto reductase family 1 member A1 OS=Homo sapiens OX=9606 GN=AKR1A1 PE=1 SV=3 |
| 477 | 5 | 3 | 3 | 3 | [Master Protein] 60S ribosomal protein L28 OS=Homo sapiens OX=9606 GN=RPL28 PE=1 SV=1 |
| 220 | 7 | 3 | 3 | 3 | [Master Protein] Enoyl-CoA hydratase OS=Homo sapiens OX=9606 PE=2 SV=1 |
| 243 | 5 | 3 | 3 | 3 | [Master Protein] VAMP (Vesicle-associated membrane protein)-associated protein B and C OS=Homo sapiens OX=9606 GN=VAPB PE=1 SV=1 |
| 370 | 11 | 3 | 3 | 3 | [Master Protein] Heat shock protein beta-1 OS=Homo sapiens OX=9606 GN=HSPB1 PE=1 SV=2 |
| 369 | 4 | 3 | 3 | 3 | [Master Protein] Phenylalanine--tRNA ligase beta subunit OS=Homo sapiens OX=9606 GN=FARSB PE=1 SV=3 |
| 571 | 5 | 3 | 3 | 3 | [Master Protein] 60S ribosomal protein L19 (Fragment) OS=Homo sapiens OX=9606 PE=2 SV=1 |
| 388 | 4 | 3 | 3 | 3 | [Master Protein] Dolichyl-diphosphooligosaccharide--protein glycosyltransferase 48 kDa subunit OS=Homo sapiens OX=9606 GN=DDOST PE=1 SV=4 |
| 339 | 5 | 3 | 3 | 3 | [Master Protein] 40S ribosomal protein S5 OS=Homo sapiens OX=9606 GN=RPS5 PE=1 SV=1 |
| 796 | 3 | 3 | 3 | 3 | [Master Protein] Peptidyl-prolyl cis-trans isomerase FKBP4 OS=Homo sapiens OX=9606 GN=FKBP4 PE=1 SV=3 |
| 160 | 18 | 3 | 3 | 3 | [Master Protein] 60S acidic ribosomal protein P0 OS=Homo sapiens OX=9606 GN=RPLP0 PE=1 SV=1 |
| 719 | 5 | 3 | 3 | 3 | [Master Protein] Coatomer subunit delta OS=Homo sapiens OX=9606 GN=ARCN1 PE=2 SV=1 |
| 32 | 4 | 3 | 3 | 3 | [Master Protein] Membrane primary amine oxidase OS=Homo sapiens OX=9606 GN=AOC3 PE=1 SV=3 |
| 34 | 8 | 3 | 3 | 3 | [Master Protein] C-1-tetrahydrofolate synthase, cytoplasmic OS=Homo sapiens OX=9606 GN=MTHFD1 PE=1 SV=3 |
| 99 | 11 | 3 | 3 | 3 | [Master Protein] cDNA FLJ60429, highly similar to Aconitate hydratase, mitochondrial OS=Homo sapiens OX=9606 PE=2 SV=1 |
| 641 | 16 | 3 | 3 | 3 | [Master Protein] Cytoplasmic FMR1 interacting protein 1 isoform A (Fragment) OS=Homo sapiens OX=9606 GN=CYFIP1 PE=2 SV=1 |
| 712 | 46 | 3 | 3 | 3 | [Master Protein] Receptor protein-tyrosine kinase (Fragment) OS=Homo sapiens OX=9606 GN=EPHB2 variant protein PE=2 SV=1 |
| 162 | 34 | 3 | 3 | 3 | [Master Protein] KIF5B-RET(NM_020630)_K22R12 fusion protein OS=Homo sapiens OX=9606 GN=KIF5B-RET(NM_020630)_K22;R12 PE=2 SV=1 |
| 829 | 8 | 3 | 3 | 3 | [Master Protein] Coatomer subunit alpha OS=Homo sapiens OX=9606 GN=COPA PE=1 SV=2 |
| 140 | 3 | 3 | 3 | 3 | [Master Protein] Myoferlin OS=Homo sapiens OX=9606 GN=MYOF PE=1 SV=1 |
| 663 | 4 | 3 | 3 | 3 | [Master Protein] 60S ribosomal protein L7a (Fragment) OS=Homo sapiens OX=9606 GN=RPL7A PE=1 SV=1 |
| 785 | 3 | 3 | 3 | 3 | [Master Protein] Integrin, alpha V OS=Homo sapiens OX=9606 GN=ITGAV PE=3 SV=1 |
| 671 | 6 | 3 | 3 | 3 | [Master Protein] Collagen alpha-1(VI) chain OS=Homo sapiens OX=9606 GN=COL6A1 PE=1 SV=1 |
| 678 | 3 | 3 | 3 | 3 | [Master Protein] Alpha-2-macroglobulin OS=Homo sapiens OX=9606 GN=A2M PE=1 SV=3 |
| 746 | 3 | 3 | 3 | 3 | [Master Protein] Scavenger receptor cysteine-rich type 1 protein M160 OS=Homo sapiens OX=9606 GN=CD163L1 PE=1 SV=2 |
| 685 | 15 | 3 | 3 | 3 | [Master Protein] Calcium-activated neutral proteinase 1 OS=Homo sapiens OX=9606 GN=CAPN1 PE=3 SV=1 |
| 789 | 1 | 3 | 3 | 3 | [Master Protein] Epididymis secretory sperm binding protein OS=Homo sapiens OX=9606 PE=2 SV=1 |
| 837 | 1 | 3 | 3 | 3 | [Master Protein] Desmoglein-2 OS=Homo sapiens OX=9606 GN=DSG2 PE=1 SV=2 |
| 19 | 12 | 3 | 3 | 3 | [Master Protein] AP-2 complex subunit mu OS=Homo sapiens OX=9606 PE=2 SV=1 |
| 166 | 6 | 3 | 3 | 3 | [Master Protein] cDNA, FLJ96465, highly similar to Homo sapiens solute carrier family 25 (mitochondrial carrier;phosphate carrier), member 3 (SLC25A3), nuclear gene encodingmitochondrial protein, transcript variant... OS=Homo sapiens OX=9606 PE=2 SV=1 |
| 774 | 30 | 3 | 3 | 3 | [Master Protein] RNA helicase OS=Homo sapiens OX=9606 GN=EIF4A2 PE=1 SV=1 |
| 346 | 7 | 3 | 3 | 3 | [Master Protein] Vacuolar proton pump subunit B OS=Homo sapiens OX=9606 PE=2 SV=1 |
| 738 | 2 | 3 | 3 | 3 | [Master Protein] cDNA FLJ52792, highly similar to Long-chain-fatty-acid--CoA ligase 5 OS=Homo sapiens OX=9606 PE=2 SV=1 |
| 808 | 3 | 3 | 3 | 3 | [Master Protein] Plakophilin-2 OS=Homo sapiens OX=9606 GN=PKP2 PE=1 SV=2 |
| 624 | 3 | 3 | 3 | 3 | [Master Protein] Nck-associated protein 1 OS=Homo sapiens OX=9606 GN=NCKAP1 PE=1 SV=1 |
| 614 | 9 | 3 | 3 | 3 | [Master Protein] Ribonuclease inhibitor OS=Homo sapiens OX=9606 PE=2 SV=1 |
| 747 | 14 | 3 | 3 | 3 | [Master Protein] Probable ATP-dependent RNA helicase DDX17 OS=Homo sapiens OX=9606 GN=DDX17 PE=1 SV=2 |
| 115 | 5 | 3 | 3 | 3 | [Master Protein] SERPINE1 mRNA binding protein 1, isoform CRA_c OS=Homo sapiens OX=9606 GN=SERBP1 PE=4 SV=1 |
| 616 | 3 | 3 | 3 | 3 | [Master Protein] Vacuolar protein sorting-associated protein 35 OS=Homo sapiens OX=9606 GN=VPS35 PE=1 SV=2 |
| 183 | 8 | 3 | 3 | 3 | [Master Protein] Tetraspanin (Fragment) OS=Homo sapiens OX=9606 GN=CD151 PE=1 SV=1 |
| 617 | 3 | 3 | 3 | 3 | [Master Protein] AIR carboxylase (Fragment) OS=Homo sapiens OX=9606 GN=PAICS PE=1 SV=1 |
| 14 | 15 | 3 | 3 | 3 | [Master Protein] UTP--glucose-1-phosphate uridylyltransferase OS=Homo sapiens OX=9606 GN=UGP2 PE=1 SV=1 |
| 365 | 32 | 2 | 11 | 17 | [Master Protein] Actin, gamma-enteric smooth muscle OS=Homo sapiens OX=9606 GN=ACTG2 PE=1 SV=1 |
| 412 | 2 | 2 | 6 | 11 | [Master Protein] cDNA FLJ53459, highly similar to Cadherin-17 OS=Homo sapiens OX=9606 PE=2 SV=1 |
| 130 | 2 | 2 | 6 | 10 | [Master Protein] Cadherin-17 OS=Homo sapiens OX=9606 GN=CDH17 PE=1 SV=3 |
| 332 | 20 | 2 | 5 | 5 | [Master Protein] Ezrin OS=Homo sapiens OX=9606 GN=EZR PE=2 SV=1 |
| 766 | 33 | 2 | 4 | 7 | [Master Protein] Uncharacterized protein OS=Homo sapiens OX=9606 PE=2 SV=1 |
| 105 | 13 | 2 | 3 | 4 | [Master Protein] L-lactate dehydrogenase OS=Homo sapiens OX=9606 PE=2 SV=1 |
| 496 | 46 | 2 | 3 | 4 | [Master Protein] Ras-related protein Rab-14 OS=Homo sapiens OX=9606 GN=RAB14 PE=1 SV=4 |
| 393 | 16 | 2 | 3 | 4 | [Master Protein] Dihydropyrimidinase-related protein 2 (Fragment) OS=Homo sapiens OX=9606 PE=2 SV=1 |
| 57 | 46 | 2 | 3 | 3 | [Master Protein] Ras-related protein Rab-10 OS=Homo sapiens OX=9606 GN=RAB10 PE=1 SV=1 |
| 758 | 6 | 2 | 3 | 3 | [Master Protein] cDNA, FLJ92164, highly similar to Homo sapiens peroxiredoxin 1 (PRDX1), mRNA OS=Homo sapiens OX=9606 PE=2 SV=1 |
| 708 | 2 | 2 | 3 | 3 | [Master Protein] Poly(rC)-binding protein 1 OS=Homo sapiens OX=9606 GN=PCBP1 PE=1 SV=2 |
| 834 | 21 | 2 | 3 | 3 | [Master Protein] Keratin, type II cytoskeletal 78 OS=Homo sapiens OX=9606 GN=KRT78 PE=1 SV=2 |
| 84 | 644 | 2 | 3 | 3 | [Master Protein] MHC class II antigen (Fragment) OS=Homo sapiens OX=9606 GN=HLA-DRB1 PE=4 SV=1 |
| 116 | 14 | 2 | 3 | 3 | [Master Protein] YWHAE/FAM22B fusion protein (Fragment) OS=Homo sapiens OX=9606 GN=YWHAE/FAM22B fusion PE=2 SV=1 |
| 584 | 12 | 2 | 3 | 3 | [Master Protein] cDNA FLJ51843, highly similar to 14-3-3 protein gamma OS=Homo sapiens OX=9606 PE=2 SV=1 |
| 213 | 16 | 2 | 2 | 5 | [Master Protein] Uncharacterized protein OS=Homo sapiens OX=9606 PE=2 SV=1 |
| 271 | 2 | 2 | 2 | 4 | [Master Protein] Endoplasmic reticulum resident protein 29 OS=Homo sapiens OX=9606 GN=ERP29 PE=1 SV=1 |
| 389 | 2 | 2 | 2 | 4 | [Master Protein] 60S ribosomal protein L10a OS=Homo sapiens OX=9606 GN=RPL10A PE=1 SV=2 |
| 6 | 39 | 2 | 2 | 4 | [Master Protein] Ubiquitin-60S ribosomal protein L40 (Fragment) OS=Homo sapiens OX=9606 GN=UBA52 PE=1 SV=1 |
| 265 | 12 | 2 | 2 | 4 | [Master Protein] Phosphoglycerate mutase (Fragment) OS=Homo sapiens OX=9606 GN=PGAM1 PE=2 SV=1 |
| 716 | 5 | 2 | 2 | 3 | [Master Protein] Syntaxin-4 OS=Homo sapiens OX=9606 GN=STX4 PE=1 SV=2 |
| 51 | 8 | 2 | 2 | 3 | [Master Protein] F-actin-capping protein subunit beta OS=Homo sapiens OX=9606 PE=2 SV=1 |
| 111 | 14 | 2 | 2 | 3 | [Master Protein] CD44 antigen OS=Homo sapiens OX=9606 PE=2 SV=1 |
| 742 | 1 | 2 | 2 | 3 | [Master Protein] Choline transporter-like protein 1 OS=Homo sapiens OX=9606 GN=SLC44A1 PE=1 SV=1 |
| 402 | 10 | 2 | 2 | 3 | [Master Protein] Mitogen-activated protein kinase OS=Homo sapiens OX=9606 GN=MAPK3 PE=1 SV=1 |
| 248 | 6 | 2 | 2 | 3 | [Master Protein] 40S ribosomal protein SA OS=Homo sapiens OX=9606 GN=RPSA PE=3 SV=1 |
| 224 | 9 | 2 | 2 | 3 | [Master Protein] Ribosomal protein L15 (Fragment) OS=Homo sapiens OX=9606 GN=RPL15 PE=1 SV=1 |
| 499 | 14 | 2 | 2 | 3 | [Master Protein] 40S ribosomal protein S2 OS=Homo sapiens OX=9606 GN=RPS2 PE=2 SV=1 |
| 334 | 4 | 2 | 2 | 3 | [Master Protein] Malectin OS=Homo sapiens OX=9606 GN=MLEC PE=1 SV=1 |
| 442 | 15 | 2 | 2 | 3 | [Master Protein] AP-1 complex subunit gamma OS=Homo sapiens OX=9606 PE=2 SV=1 |
| 592 | 7 | 2 | 2 | 3 | [Master Protein] GATOR complex protein SEC13 OS=Homo sapiens OX=9606 GN=SEC13 PE=1 SV=1 |
| 351 | 10 | 2 | 2 | 3 | [Master Protein] Basigin (Fragment) OS=Homo sapiens OX=9606 GN=BSG PE=1 SV=8 |
| 573 | 4 | 2 | 2 | 3 | [Master Protein] Extended synaptotagmin-2 OS=Homo sapiens OX=9606 GN=ESYT2 PE=1 SV=1 |
| 835 | 9 | 2 | 2 | 3 | [Master Protein] UDP-glucose 6-dehydrogenase OS=Homo sapiens OX=9606 GN=UGDH PE=1 SV=1 |
| 636 | 5 | 2 | 2 | 3 | [Master Protein] Glutamyl-tRNA synthetase OS=Homo sapiens OX=9606 PE=2 SV=1 |
| 112 | 5 | 2 | 2 | 3 | [Master Protein] TXNDC5 protein OS=Homo sapiens OX=9606 GN=TXNDC5 PE=1 SV=1 |
| 777 | 5 | 2 | 2 | 3 | [Master Protein] MICOS complex subunit MIC19 OS=Homo sapiens OX=9606 GN=CHCHD3 PE=1 SV=2 |
| 149 | 4 | 2 | 2 | 3 | [Master Protein] Villin-1 (Fragment) OS=Homo sapiens OX=9606 PE=2 SV=1 |
| 26 | 18 | 2 | 2 | 3 | [Master Protein] Adenylyl cyclase-associated protein 1 OS=Homo sapiens OX=9606 PE=2 SV=1 |
| 373 | 3 | 2 | 2 | 3 | [Master Protein] Apolipoprotein H OS=Homo sapiens OX=9606 PE=2 SV=1 |
| 684 | 4 | 2 | 2 | 3 | [Master Protein] Epididymis luminal protein 210 OS=Homo sapiens OX=9606 GN=HEL-210 PE=2 SV=1 |
| 686 | 4 | 2 | 2 | 3 | [Master Protein] cDNA FLJ60782, highly similar to Rho-GTPase-activating protein 1 OS=Homo sapiens OX=9606 PE=2 SV=1 |
| 214 | 15 | 2 | 2 | 3 | [Master Protein] Alanine--tRNA ligase OS=Homo sapiens OX=9606 GN=AARS1 PE=1 SV=4 |
| 420 | 5 | 2 | 2 | 2 | [Master Protein] Phosphoinositide phospholipase C OS=Homo sapiens OX=9606 PE=2 SV=1 |
| 781 | 9 | 2 | 2 | 2 | [Master Protein] 60S ribosomal protein L26 OS=Homo sapiens OX=9606 GN=RPL26 PE=1 SV=1 |
| 425 | 9 | 2 | 2 | 2 | [Master Protein] Aminopeptidase OS=Homo sapiens OX=9606 PE=2 SV=1 |
| 427 | 12 | 2 | 2 | 2 | [Master Protein] Lysosome membrane protein 2 OS=Homo sapiens OX=9606 GN=SCARB2 PE=1 SV=1 |
| 415 | 9 | 2 | 2 | 2 | [Master Protein] Cation-dependent mannose-6-phosphate receptor variant (Fragment) OS=Homo sapiens OX=9606 PE=2 SV=1 |
| 598 | 5 | 2 | 2 | 2 | [Master Protein] Dimethylargininase (Fragment) OS=Homo sapiens OX=9606 GN=DDAH2 PE=1 SV=2 |
| 376 | 3 | 2 | 2 | 2 | [Master Protein] Copine-3 OS=Homo sapiens OX=9606 GN=CPNE3 PE=1 SV=1 |
| 790 | 2 | 2 | 2 | 2 | [Master Protein] Adenosylhomocysteinase OS=Homo sapiens OX=9606 GN=AHCY PE=1 SV=4 |
| 792 | 1 | 2 | 2 | 2 | [Master Protein] Endonuclease domain-containing 1 protein OS=Homo sapiens OX=9606 GN=ENDOD1 PE=1 SV=2 |
| 795 | 1 | 2 | 2 | 2 | [Master Protein] Dermcidin OS=Homo sapiens OX=9606 GN=DCD PE=1 SV=2 |
| 807 | 2 | 2 | 2 | 2 | [Master Protein] DnaJ homolog subfamily A member 2 OS=Homo sapiens OX=9606 GN=DNAJA2 PE=1 SV=1 |
| 378 | 5 | 2 | 2 | 2 | [Master Protein] Ras-related protein R-Ras2 OS=Homo sapiens OX=9606 GN=RRAS2 PE=1 SV=1 |
| 375 | 4 | 2 | 2 | 2 | [Master Protein] Programmed cell death protein 4 OS=Homo sapiens OX=9606 PE=2 SV=1 |
| 820 | 7 | 2 | 2 | 2 | [Master Protein] Coatomer subunit beta' OS=Homo sapiens OX=9606 GN=COPB2 PE=1 SV=2 |
| 372 | 14 | 2 | 2 | 2 | [Master Protein] Aldehyde dehydrogenase family 3 member A2 OS=Homo sapiens OX=9606 GN=ALDH3A2 PE=1 SV=1 |
| 830 | 1 | 2 | 2 | 2 | [Master Protein] Membrane-associated progesterone receptor component 2 OS=Homo sapiens OX=9606 GN=PGRMC2 PE=1 SV=1 |
| 367 | 8 | 2 | 2 | 2 | [Master Protein] cDNA FLJ58823, highly similar to Thromboxane-A synthase OS=Homo sapiens OX=9606 PE=2 SV=1 |
| 844 | 2 | 2 | 2 | 2 | [Master Protein] Cathepsin X OS=Homo sapiens OX=9606 PE=2 SV=1 |
| 356 | 8 | 2 | 2 | 2 | [Master Protein] Monoamine oxidase A (Fragment) OS=Homo sapiens OX=9606 GN=MAOA PE=4 SV=1 |
| 347 | 13 | 2 | 2 | 2 | [Master Protein] Protein transport protein Sec61 subunit alpha isoform 1 OS=Homo sapiens OX=9606 GN=SEC61A1 PE=1 SV=1 |
| 851 | 1 | 2 | 2 | 2 | [Master Protein] Keratin, type I cytoskeletal 20 OS=Homo sapiens OX=9606 GN=KRT20 PE=1 SV=1 |
| 401 | 4 | 2 | 2 | 2 | [Master Protein] cDNA FLJ55534, highly similar to 4-trimethylaminobutyraldehyde dehydrogenase OS=Homo sapiens OX=9606 PE=2 SV=1 |
| 765 | 4 | 2 | 2 | 2 | [Master Protein] CCT-beta OS=Homo sapiens OX=9606 GN=HEL-S-100n PE=1 SV=1 |
| 730 | 2 | 2 | 2 | 2 | [Master Protein] Epoxide hydrolase OS=Homo sapiens OX=9606 GN=EPHX1 PE=2 SV=1 |
| 433 | 16 | 2 | 2 | 2 | [Master Protein] Serine/threonine-protein phosphatase OS=Homo sapiens OX=9606 GN=PPP1CC PE=1 SV=1 |
| 515 | 10 | 2 | 2 | 2 | [Master Protein] 26S proteasome regulatory subunit 8 (Fragment) OS=Homo sapiens OX=9606 GN=PSMC5 PE=1 SV=1 |
| 670 | 3 | 2 | 2 | 2 | [Master Protein] Cathepsin S OS=Homo sapiens OX=9606 GN=CTSS PE=1 SV=3 |
| 659 | 7 | 2 | 2 | 2 | [Master Protein] AP-2 complex subunit alpha-1 OS=Homo sapiens OX=9606 GN=AP2A1 PE=1 SV=3 |
| 530 | 6 | 2 | 2 | 2 | [Master Protein] cDNA FLJ41407 fis, clone BRHIP2000819, moderately similar to Human actin binding protein p57 OS=Homo sapiens OX=9606 PE=2 SV=1 |
| 533 | 5 | 2 | 2 | 2 | [Master Protein] Guanine nucleotide-binding protein G, alpha subunit variant (Fragment) OS=Homo sapiens OX=9606 PE=2 SV=1 |
| 655 | 4 | 2 | 2 | 2 | [Master Protein] 60S ribosomal protein L29 OS=Homo sapiens OX=9606 GN=RPL29 PE=1 SV=1 |
| 647 | 6 | 2 | 2 | 2 | [Master Protein] cDNA FLJ55496, highly similar to Aminopeptidase N OS=Homo sapiens OX=9606 PE=2 SV=1 |
| 545 | 8 | 2 | 2 | 2 | [Master Protein] GTP-binding nuclear protein Ran OS=Homo sapiens OX=9606 GN=RAN PE=1 SV=1 |
| 548 | 6 | 2 | 2 | 2 | [Master Protein] Mucin-2 OS=Homo sapiens OX=9606 GN=MUC2 PE=1 SV=2 |
| 635 | 12 | 2 | 2 | 2 | [Master Protein] MICOS complex subunit MIC60 OS=Homo sapiens OX=9606 PE=2 SV=1 |
| 512 | 2 | 2 | 2 | 2 | [Master Protein] Serine incorporator 5 OS=Homo sapiens OX=9606 GN=SERINC5 PE=1 SV=1 |
| 556 | 1 | 2 | 2 | 2 | [Master Protein] Leucine-rich repeat-containing protein 59 OS=Homo sapiens OX=9606 GN=LRRC59 PE=1 SV=1 |
| 631 | 2 | 2 | 2 | 2 | [Master Protein] Mast cell carboxypeptidase A OS=Homo sapiens OX=9606 GN=CPA3 PE=1 SV=2 |
| 629 | 4 | 2 | 2 | 2 | [Master Protein] Solute carrier family 17 (Anion/sugar transporter), member 5, isoform CRA_a OS=Homo sapiens OX=9606 GN=SLC17A5 PE=4 SV=1 |
| 570 | 1 | 2 | 2 | 2 | [Master Protein] Corticosteroid 11-beta-dehydrogenase isozyme 2 OS=Homo sapiens OX=9606 GN=HSD11B2 PE=1 SV=2 |
| 628 | 6 | 2 | 2 | 2 | [Master Protein] Cation-transporting ATPase OS=Homo sapiens OX=9606 GN=ATP13A1 PE=3 SV=1 |
| 578 | 4 | 2 | 2 | 2 | [Master Protein] Neuroplastin OS=Homo sapiens OX=9606 GN=NPTN PE=1 SV=2 |
| 621 | 6 | 2 | 2 | 2 | [Master Protein] Eukaryotic translation initiation factor 3 subunit B OS=Homo sapiens OX=9606 GN=EIF3B PE=2 SV=1 |
| 619 | 1 | 2 | 2 | 2 | [Master Protein] Filaggrin-2 OS=Homo sapiens OX=9606 GN=FLG2 PE=1 SV=1 |
| 593 | 6 | 2 | 2 | 2 | [Master Protein] Acyl-Coenzyme A dehydrogenase, very long chain, isoform CRA_e OS=Homo sapiens OX=9606 GN=ACADVL PE=2 SV=1 |
| 604 | 2 | 2 | 2 | 2 | [Master Protein] Estradiol 17-beta-dehydrogenase 11 OS=Homo sapiens OX=9606 GN=HSD17B11 PE=1 SV=3 |
| 601 | 3 | 2 | 2 | 2 | [Master Protein] Anion exchange protein OS=Homo sapiens OX=9606 GN=SLC4A4 PE=2 SV=1 |
| 560 | 1 | 2 | 2 | 2 | [Master Protein] Protein PRRC1 OS=Homo sapiens OX=9606 GN=PRRC1 PE=1 SV=1 |
| 431 | 3 | 2 | 2 | 2 | [Master Protein] Lysosomal-associated membrane protein 1, isoform CRA_a OS=Homo sapiens OX=9606 GN=LAMP1 PE=3 SV=1 |
| 690 | 2 | 2 | 2 | 2 | [Master Protein] Chymase OS=Homo sapiens OX=9606 GN=CMA1 PE=1 SV=1 |
| 691 | 1 | 2 | 2 | 2 | [Master Protein] Golgi membrane protein 1 OS=Homo sapiens OX=9606 GN=GOLM1 PE=1 SV=1 |
| 759 | 2 | 2 | 2 | 2 | [Master Protein] Electron transfer flavoprotein subunit beta OS=Homo sapiens OX=9606 GN=ETFB PE=1 SV=3 |
| 445 | 5 | 2 | 2 | 2 | [Master Protein] Lysine--tRNA ligase OS=Homo sapiens OX=9606 GN=KARS1 PE=1 SV=3 |
| 446 | 10 | 2 | 2 | 2 | [Master Protein] Receptor-type tyrosine-protein phosphatase C OS=Homo sapiens OX=9606 GN=PTPRC PE=1 SV=3 |
| 447 | 9 | 2 | 2 | 2 | [Master Protein] Ras GTPase-activating-like protein IQGAP2 (Fragment) OS=Homo sapiens OX=9606 GN=IQGAP2 PE=1 SV=1 |
| 753 | 2 | 2 | 2 | 2 | [Master Protein] F-actin-capping protein subunit alpha OS=Homo sapiens OX=9606 PE=2 SV=1 |
| 455 | 232 | 2 | 2 | 2 | [Master Protein] IGH + IGL c119_heavy_IGHV3-30_IGHD4-17_IGHJ4 (Fragment) OS=Homo sapiens OX=9606 PE=2 SV=1 |
| 752 | 7 | 2 | 2 | 2 | [Master Protein] Eukaryotic translation initiation factor 3 subunit A OS=Homo sapiens OX=9606 GN=eIF3a PE=2 SV=1 |
| 460 | 17 | 2 | 2 | 2 | [Master Protein] cDNA FLJ54170, highly similar to Cytosolic nonspecific dipeptidase OS=Homo sapiens OX=9606 PE=2 SV=1 |
| 461 | 4 | 2 | 2 | 2 | [Master Protein] Unconventional myosin-Ia OS=Homo sapiens OX=9606 GN=MYO1A PE=1 SV=1 |
| 740 | 10 | 2 | 2 | 2 | [Master Protein] Reticulon OS=Homo sapiens OX=9606 GN=RTN4 PE=2 SV=1 |
| 504 | 2 | 2 | 2 | 2 | [Master Protein] RAB5C, member RAS oncogene family, isoform CRA_a OS=Homo sapiens OX=9606 GN=RAB5C PE=4 SV=1 |
| 736 | 3 | 2 | 2 | 2 | [Master Protein] Eukaryotic translation initiation factor 2 subunit 1 OS=Homo sapiens OX=9606 GN=EIF2S1 PE=2 SV=1 |
| 724 | 2 | 2 | 2 | 2 | [Master Protein] Delta(3,5)-Delta(2,4)-dienoyl-CoA isomerase, mitochondrial OS=Homo sapiens OX=9606 GN=ECH1 PE=1 SV=2 |
| 722 | 7 | 2 | 2 | 2 | [Master Protein] Integrin alpha-X OS=Homo sapiens OX=9606 GN=ITGAX PE=1 SV=3 |
| 717 | 13 | 2 | 2 | 2 | [Master Protein] Cathepsin D OS=Homo sapiens OX=9606 GN=CTSD PE=1 SV=1 |
| 481 | 9 | 2 | 2 | 2 | [Master Protein] Glutamine--fructose-6-phosphate transaminase (isomerizing) OS=Homo sapiens OX=9606 GN=GFPT1 PE=1 SV=1 |
| 485 | 6 | 2 | 2 | 2 | [Master Protein] Prohibitin OS=Homo sapiens OX=9606 GN=PHB2 PE=1 SV=1 |
| 488 | 3 | 2 | 2 | 2 | [Master Protein] Syntaxin-12 OS=Homo sapiens OX=9606 GN=STX12 PE=1 SV=1 |
| 490 | 4 | 2 | 2 | 2 | [Master Protein] Leukotriene A4 hydrolase variant (Fragment) OS=Homo sapiens OX=9606 PE=2 SV=1 |
| 699 | 12 | 2 | 2 | 2 | [Master Protein] Succinate dehydrogenase [ubiquinone] flavoprotein subunit, mitochondrial OS=Homo sapiens OX=9606 GN=SDHA PE=1 SV=2 |
| 693 | 4 | 2 | 2 | 2 | [Master Protein] ATP-dependent RNA helicase DDX1 OS=Homo sapiens OX=9606 GN=DDX1 PE=2 SV=1 |
| 692 | 4 | 2 | 2 | 2 | [Master Protein] Proteasome subunit alpha type-3 OS=Homo sapiens OX=9606 GN=PSMA3 PE=1 SV=2 |
| 469 | 15 | 2 | 2 | 2 | [Master Protein] Heterogeneous nuclear ribonucleoprotein R OS=Homo sapiens OX=9606 GN=HNRNPR PE=1 SV=1 |
| 1 | 10 | 2 | 2 | 2 | [Master Protein] Carcinoembryonic antigen-related cell adhesion molecule 5 OS=Homo sapiens OX=9606 GN=CEACAM5 PE=1 SV=1 |
| 857 | 1 | 2 | 2 | 2 | [Master Protein] Cathepsin G OS=Homo sapiens OX=9606 GN=CTSG PE=1 SV=2 |
| 94 | 5 | 2 | 2 | 2 | [Master Protein] Complex I-75kD OS=Homo sapiens OX=9606 PE=3 SV=1 |
| 136 | 3 | 2 | 2 | 2 | [Master Protein] Lectin, mannose-binding, 1 variant (Fragment) OS=Homo sapiens OX=9606 PE=2 SV=1 |
| 216 | 7 | 2 | 2 | 2 | [Master Protein] HCG40889, isoform CRA_b OS=Homo sapiens OX=9606 GN=hCG_40889 PE=4 SV=1 |
| 297 | 5 | 2 | 2 | 2 | [Master Protein] Dolichyl-diphosphooligosaccharide--protein glycosyltransferase subunit 2 OS=Homo sapiens OX=9606 PE=2 SV=1 |
| 298 | 10 | 2 | 2 | 2 | [Master Protein] Eukaryotic translation initiation factor 3 subunit E OS=Homo sapiens OX=9606 GN=EIF3E PE=2 SV=1 |
| 291 | 7 | 2 | 2 | 2 | [Master Protein] Kinectin OS=Homo sapiens OX=9606 GN=KTN1 PE=1 SV=1 |
| 194 | 6 | 2 | 2 | 2 | [Master Protein] Malate dehydrogenase OS=Homo sapiens OX=9606 GN=MDH2 PE=2 SV=1 |
| 317 | 6 | 2 | 2 | 2 | [Master Protein] Cell migration-inducing protein 22 OS=Homo sapiens OX=9606 PE=2 SV=1 |
| 195 | 8 | 2 | 2 | 2 | [Master Protein] cDNA FLJ51879, highly similar to Prenylcysteine oxidase OS=Homo sapiens OX=9606 PE=2 SV=1 |
| 96 | 8 | 2 | 2 | 2 | [Master Protein] cDNA, FLJ95462, highly similar to Homo sapiens fatty-acid-Coenzyme A ligase, long-chain 3 (FACL3),mRNA OS=Homo sapiens OX=9606 PE=2 SV=1 |
| 141 | 20 | 2 | 2 | 2 | [Master Protein] Uncharacterized protein (Fragment) OS=Homo sapiens OX=9606 PE=3 SV=1 |
| 300 | 10 | 2 | 2 | 2 | [Master Protein] Unconventional myosin-6 OS=Homo sapiens OX=9606 GN=Myo6-008 PE=2 SV=1 |
| 182 | 4 | 2 | 2 | 2 | [Master Protein] Dehydrogenase/reductase SDR family member 7 OS=Homo sapiens OX=9606 GN=DHRS7 PE=1 SV=1 |
| 337 | 5 | 2 | 2 | 2 | [Master Protein] Protein TMED7-TICAM2 OS=Homo sapiens OX=9606 GN=TMED7-TICAM2 PE=3 SV=1 |
| 29 | 434 | 2 | 2 | 2 | [Master Protein] MHC class II antigen (Fragment) OS=Homo sapiens OX=9606 GN=HLA-DQB1 PE=4 SV=1 |
| 280 | 3 | 2 | 2 | 2 | [Master Protein] Glutathione reductase OS=Homo sapiens OX=9606 GN=HEL-75 PE=2 SV=1 |
| 281 | 11 | 2 | 2 | 2 | [Master Protein] cDNA FLJ59702, highly similar to NADPH--cytochrome P450 reductase OS=Homo sapiens OX=9606 PE=2 SV=1 |
| 200 | 20 | 2 | 2 | 2 | [Master Protein] Aminopeptidase (Fragment) OS=Homo sapiens OX=9606 GN=ERAP1 PE=2 SV=1 |
| 296 | 5 | 2 | 2 | 2 | [Master Protein] cDNA FLJ44754 fis, clone BRACE3030748, highly similar to Guanine nucleotide-binding protein G(i), alpha-2 subunit OS=Homo sapiens OX=9606 PE=2 SV=1 |
| 177 | 4 | 2 | 2 | 2 | [Master Protein] Vasodilator-stimulated phosphoprotein (Fragment) OS=Homo sapiens OX=9606 GN=VASP PE=1 SV=1 |
| 21 | 3 | 2 | 2 | 2 | [Master Protein] Parkinson disease protein 7 OS=Homo sapiens OX=9606 GN=PARK7 PE=1 SV=1 |
| 153 | 10 | 2 | 2 | 2 | [Master Protein] Glutamate dehydrogenase OS=Homo sapiens OX=9606 PE=2 SV=1 |
| 172 | 16 | 2 | 2 | 2 | [Master Protein] Far upstream element-binding protein 1 OS=Homo sapiens OX=9606 GN=FUBP1 PE=1 SV=3 |
| 13 | 10 | 2 | 2 | 2 | [Master Protein] Heterogeneous nuclear ribonucleoprotein M (Fragment) OS=Homo sapiens OX=9606 GN=HNRNPM PE=1 SV=8 |
| 81 | 4 | 2 | 2 | 2 | [Master Protein] Gc-globulin OS=Homo sapiens OX=9606 GN=GC PE=1 SV=1 |
| 249 | 13 | 2 | 2 | 2 | [Master Protein] Collagen-binding protein OS=Homo sapiens OX=9606 PE=2 SV=1 |
| 98 | 7 | 2 | 2 | 2 | [Master Protein] LIM domain and actin-binding protein 1 OS=Homo sapiens OX=9606 GN=LIMA1 PE=1 SV=1 |
| 92 | 12 | 2 | 2 | 2 | [Master Protein] START domain-containing protein 10 (Fragment) OS=Homo sapiens OX=9606 GN=STARD10 PE=1 SV=1 |
| 219 | 8 | 2 | 2 | 2 | [Master Protein] 60S ribosomal protein L13a OS=Homo sapiens OX=9606 PE=2 SV=1 |
| 159 | 22 | 2 | 2 | 2 | [Master Protein] Tight junction protein 2 (Zona occludens 2), isoform CRA_c OS=Homo sapiens OX=9606 GN=TJP2 PE=4 SV=1 |
| 46 | 7 | 2 | 2 | 2 | [Master Protein] Interleukin enhancer binding factor 3 isoform c variant (Fragment) OS=Homo sapiens OX=9606 PE=2 SV=1 |
| 88 | 4 | 2 | 2 | 2 | [Master Protein] Sodium/potassium-transporting ATPase subunit beta (Fragment) OS=Homo sapiens OX=9606 GN=ATP1B3 PE=2 SV=1 |
| 330 | 11 | 2 | 2 | 2 | [Master Protein] 60S ribosomal protein L18a OS=Homo sapiens OX=9606 PE=2 SV=1 |
| 86 | 5 | 2 | 2 | 2 | [Master Protein] Annexin OS=Homo sapiens OX=9606 GN=ANXA3 PE=1 SV=1 |
| 223 | 3 | 2 | 2 | 2 | [Master Protein] Eukaryotic translation initiation factor 3 subunit I OS=Homo sapiens OX=9606 GN=EIF3S2 PE=2 SV=1 |
| 4 | 16 | 2 | 2 | 2 | [Master Protein] Rho-related GTP-binding protein RhoC (Fragment) OS=Homo sapiens OX=9606 GN=RHOC PE=1 SV=1 |
| 151 | 10 | 2 | 2 | 2 | [Master Protein] Protein scribble homolog OS=Homo sapiens OX=9606 GN=SCRIB PE=1 SV=1 |
| 449 | 6 | 1 | 7 | 7 | [Master Protein] WD repeat domain 1, isoform CRA_a (Fragment) OS=Homo sapiens OX=9606 GN=WDR1 PE=2 SV=1 |
| 815 | 6 | 1 | 7 | 7 | [Master Protein] Epididymis secretory protein Li 52 OS=Homo sapiens OX=9606 GN=HEL-S-52 PE=2 SV=1 |
| 108 | 46 | 1 | 5 | 14 | [Master Protein] Tubulin beta chain OS=Homo sapiens OX=9606 GN=TUBB2C PE=2 SV=1 |
| 555 | 54 | 1 | 5 | 14 | [Master Protein] cDNA FLJ52378, highly similar to Tubulin beta-7 chain OS=Homo sapiens OX=9606 PE=2 SV=1 |
| 199 | 7 | 1 | 5 | 5 | [Master Protein] Keratin, type II cytoskeletal 1b OS=Homo sapiens OX=9606 GN=KRT77 PE=1 SV=3 |
| 320 | 9 | 1 | 4 | 6 | [Master Protein] Epididymis luminal secretory protein 52 OS=Homo sapiens OX=9606 GN=EL52 PE=2 SV=1 |
| 290 | 4 | 1 | 4 | 5 | [Master Protein] Erlin-1 OS=Homo sapiens OX=9606 GN=ERLIN1 PE=1 SV=2 |
| 702 | 36 | 1 | 4 | 5 | [Master Protein] IGL@ protein OS=Homo sapiens OX=9606 GN=IGL@ PE=1 SV=1 |
| 733 | 3 | 1 | 4 | 5 | [Master Protein] Erlin-2 OS=Homo sapiens OX=9606 GN=ERLIN2 PE=1 SV=1 |
| 73 | 6 | 1 | 4 | 4 | [Master Protein] Protein transport protein SEC23 OS=Homo sapiens OX=9606 GN=SEC23A PE=1 SV=1 |
| 832 | 7 | 1 | 4 | 4 | [Master Protein] Protein transport protein SEC23 OS=Homo sapiens OX=9606 PE=2 SV=1 |
| 311 | 328 | 1 | 3 | 5 | [Master Protein] MHC class I antigen OS=Homo sapiens OX=9606 GN=HLA-C PE=3 SV=1 |
| 234 | 19 | 1 | 3 | 4 | [Master Protein] AP complex subunit beta OS=Homo sapiens OX=9606 GN=DKFZp781K0743 PE=2 SV=1 |
| 625 | 20 | 1 | 3 | 4 | [Master Protein] Immunoglobulin heavy constant gamma 3 (Fragment) OS=Homo sapiens OX=9606 GN=IGHG3 PE=1 SV=1 |
| 385 | 573 | 1 | 3 | 4 | [Master Protein] MHC class I antigen OS=Homo sapiens OX=9606 GN=HLA-B PE=3 SV=1 |
| 856 | 10 | 1 | 3 | 4 | [Master Protein] AP-1 complex subunit beta-1 OS=Homo sapiens OX=9606 GN=AP1B1 PE=1 SV=2 |
| 607 | 4 | 1 | 3 | 3 | [Master Protein] ADP/ATP translocase 2 OS=Homo sapiens OX=9606 GN=SLC25A5 PE=1 SV=7 |
| 178 | 16 | 1 | 2 | 4 | [Master Protein] Guanine nucleotide-binding protein subunit beta-4 OS=Homo sapiens OX=9606 GN=GNB4 PE=1 SV=3 |
| 315 | 14 | 1 | 2 | 3 | [Master Protein] Testicular secretory protein Li 7 OS=Homo sapiens OX=9606 PE=2 SV=1 |
| 681 | 22 | 1 | 2 | 2 | [Master Protein] Cell division control protein 42 homolog OS=Homo sapiens OX=9606 GN=CDC42 PE=1 SV=2 |
| 450 | 264 | 1 | 2 | 2 | [Master Protein] IgG L chain OS=Homo sapiens OX=9606 PE=2 SV=1 |
| 557 | 47 | 1 | 2 | 2 | [Master Protein] Ras-related protein Rab-8B (Fragment) OS=Homo sapiens OX=9606 GN=RAB8B PE=1 SV=1 |
| 423 | 47 | 1 | 2 | 2 | [Master Protein] Ras-related protein Rab-35 (Fragment) OS=Homo sapiens OX=9606 GN=RAB35 PE=1 SV=1 |
| 794 | 15 | 1 | 2 | 2 | [Master Protein] Ras-related protein Rap-1A OS=Homo sapiens OX=9606 GN=RAP1A PE=1 SV=1 |
| 263 | 11 | 1 | 2 | 2 | [Master Protein] cDNA FLJ40884 fis, clone UTERU2000607, highly similar to Alpha-actinin-1 OS=Homo sapiens OX=9606 PE=2 SV=1 |
| 546 | 2 | 1 | 2 | 2 | [Master Protein] cDNA FLJ60461, highly similar to Peroxiredoxin-2 OS=Homo sapiens OX=9606 PE=2 SV=1 |
| 750 | 262 | 1 | 2 | 2 | [Master Protein] IGL c2705_light_IGKV3-20_IGKJ1 (Fragment) OS=Homo sapiens OX=9606 PE=2 SV=1 |
| 535 | 45 | 1 | 2 | 2 | [Master Protein] Ras-related protein Rab-8A OS=Homo sapiens OX=9606 GN=RAB8A PE=1 SV=1 |
| 503 | 15 | 1 | 2 | 2 | [Master Protein] Poly(rC)-binding protein 3 OS=Homo sapiens OX=9606 GN=PCBP3 PE=1 SV=1 |
| 203 | 15 | 1 | 2 | 2 | [Master Protein] Tyrosine 3-monooxygenase/tryptophan 5-monooxygenase activation protein, beta polypeptide variant (Fragment) OS=Homo sapiens OX=9606 PE=2 SV=1 |
| 135 | 48 | 1 | 2 | 2 | [Master Protein] H.sapiens ras-related Hrab1A protein OS=Homo sapiens OX=9606 GN=RAB1A PE=2 SV=1 |
| 48 | 28 | 1 | 2 | 2 | [Master Protein] Ras-related C3 botulinum toxin substrate 2 (Fragment) OS=Homo sapiens OX=9606 GN=RAC2 PE=1 SV=1 |
| 627 | 22 | 1 | 2 | 2 | [Master Protein] Ras-related protein Rap-1b OS=Homo sapiens OX=9606 GN=RAP1B PE=1 SV=1 |
| 147 | 374 | 1 | 2 | 2 | [Master Protein] MHC class II antigen (Fragment) OS=Homo sapiens OX=9606 GN=HLA-DRB3 PE=4 SV=1 |
| 325 | 19 | 1 | 2 | 2 | [Master Protein] Fructose-bisphosphate aldolase OS=Homo sapiens OX=9606 PE=2 SV=1 |
| 3 | 112 | 1 | 2 | 2 | [Master Protein] V2-17 protein (Fragment) OS=Homo sapiens OX=9606 GN=V2-17 PE=1 SV=1 |
| 396 | 9 | 1 | 2 | 2 | [Master Protein] cDNA, FLJ92996, highly similar to Homo sapiens guanine nucleotide binding protein (G protein), beta polypeptide 1 (GNB1), mRNA OS=Homo sapiens OX=9606 PE=2 SV=1 |
| 825 | 206 | 1 | 2 | 2 | [Master Protein] IgG H chain OS=Homo sapiens OX=9606 PE=2 SV=1 |
| 510 | 1 | 1 | 1 | 3 | [Master Protein] Membrane cofactor protein OS=Homo sapiens OX=9606 GN=CD46 PE=1 SV=3 |
| 600 | 3 | 1 | 1 | 2 | [Master Protein] Transaldolase OS=Homo sapiens OX=9606 GN=TALDO1 PE=1 SV=1 |
| 54 | 2 | 1 | 1 | 2 | [Master Protein] Dolichyl-diphosphooligosaccharide--protein glycosyltransferase subunit STT3A OS=Homo sapiens OX=9606 GN=STT3A PE=1 SV=2 |
| 59 | 4 | 1 | 1 | 2 | [Master Protein] 2',3'-cyclic-nucleotide 3'-phosphodiesterase OS=Homo sapiens OX=9606 PE=2 SV=1 |
| 245 | 6 | 1 | 1 | 2 | [Master Protein] Prosomal P27K protein (Fragment) OS=Homo sapiens OX=9606 GN=PSMA6 PE=4 SV=1 |
| 278 | 13 | 1 | 1 | 2 | [Master Protein] Corneodesmosin OS=Homo sapiens OX=9606 PE=4 SV=1 |
| 353 | 2 | 1 | 1 | 2 | [Master Protein] Endophilin-B1 OS=Homo sapiens OX=9606 GN=SH3GLB1 PE=1 SV=1 |
| 274 | 16 | 1 | 1 | 2 | [Master Protein] cAMP-dependent protein kinase catalytic subunit alpha (Fragment) OS=Homo sapiens OX=9606 GN=PRKACA PE=1 SV=1 |
| 434 | 6 | 1 | 1 | 2 | [Master Protein] Transmembrane 9 superfamily member (Fragment) OS=Homo sapiens OX=9606 GN=TM9SF3 PE=2 SV=2 |
| 486 | 5 | 1 | 1 | 2 | [Master Protein] AP-3 complex subunit beta OS=Homo sapiens OX=9606 PE=2 SV=1 |
| 482 | 4 | 1 | 1 | 2 | [Master Protein] cDNA, FLJ79350, highly similar to Protein transport protein Sec24C OS=Homo sapiens OX=9606 PE=2 SV=1 |
| 612 | 2 | 1 | 1 | 2 | [Master Protein] MARCKS protein (Fragment) OS=Homo sapiens OX=9606 GN=MARCKS PE=2 SV=1 |
| 408 | 1 | 1 | 1 | 2 | [Master Protein] Gamma-soluble NSF attachment protein OS=Homo sapiens OX=9606 GN=NAPG PE=1 SV=1 |
| 20 | 5 | 1 | 1 | 2 | [Master Protein] Adenine phosphoribosyltransferase OS=Homo sapiens OX=9606 GN=APRT PE=1 SV=1 |
| 739 | 2 | 1 | 1 | 2 | [Master Protein] MHC class I antigen (Fragment) OS=Homo sapiens OX=9606 GN=HLA-B PE=3 SV=1 |
| 225 | 8 | 1 | 1 | 2 | [Master Protein] Stress-70 protein, mitochondrial OS=Homo sapiens OX=9606 GN=HSPA9 PE=1 SV=1 |
| 567 | 2 | 1 | 1 | 2 | [Master Protein] Proteasome subunit alpha type OS=Homo sapiens OX=9606 PE=2 SV=1 |
| 321 | 4 | 1 | 1 | 2 | [Master Protein] Outer mitochondrial membrane protein porin 2 (Fragment) OS=Homo sapiens OX=9606 GN=VDAC2 PE=1 SV=1 |
| 37 | 4 | 1 | 1 | 2 | [Master Protein] Kininogen 1, isoform CRA_a OS=Homo sapiens OX=9606 GN=KNG1 PE=4 SV=1 |
| 398 | 8 | 1 | 1 | 2 | [Master Protein] Tetraspanin OS=Homo sapiens OX=9606 PE=2 SV=1 |
| 137 | 5 | 1 | 1 | 2 | [Master Protein] T-complex protein 1 subunit eta (Fragment) OS=Homo sapiens OX=9606 GN=CCT7 PE=1 SV=1 |
| 799 | 2 | 1 | 1 | 2 | [Master Protein] cDNA, FLJ94551 OS=Homo sapiens OX=9606 PE=2 SV=1 |
| 588 | 8 | 1 | 1 | 2 | [Master Protein] Trifunctional enzyme subunit beta, mitochondrial OS=Homo sapiens OX=9606 GN=HADHB PE=1 SV=1 |
| 411 | 2 | 1 | 1 | 2 | [Master Protein] Signal peptidase complex subunit 3 OS=Homo sapiens OX=9606 GN=SPCS3 PE=3 SV=1 |
| 196 | 3 | 1 | 1 | 2 | [Master Protein] cDNA FLJ57882, highly similar to Pre-mRNA-processing-splicing factor 8 OS=Homo sapiens OX=9606 PE=2 SV=1 |
| 236 | 4 | 1 | 1 | 2 | [Master Protein] RNA transcription, translation and transport factor protein (Fragment) OS=Homo sapiens OX=9606 GN=RTRAF PE=1 SV=1 |
| 197 | 9 | 1 | 1 | 2 | [Master Protein] Galectin OS=Homo sapiens OX=9606 PE=2 SV=1 |
| 714 | 1 | 1 | 1 | 2 | [Master Protein] Ras-related protein Rab-25 OS=Homo sapiens OX=9606 GN=RAB25 PE=1 SV=2 |
| 701 | 3 | 1 | 1 | 1 | [Master Protein] Golgi apparatus protein 1 OS=Homo sapiens OX=9606 GN=GLG1 PE=1 SV=2 |
| 634 | 2 | 1 | 1 | 1 | [Master Protein] 40S ribosomal protein S11 OS=Homo sapiens OX=9606 GN=RPS11 PE=1 SV=3 |
| 113 | 5 | 1 | 1 | 1 | [Master Protein] 3'(2'),5'-bisphosphate nucleotidase 1 (Fragment) OS=Homo sapiens OX=9606 GN=BPNT1 PE=1 SV=2 |
| 645 | 5 | 1 | 1 | 1 | [Master Protein] Phospholipid-transporting ATPase OS=Homo sapiens OX=9606 PE=2 SV=1 |
| 100 | 4 | 1 | 1 | 1 | [Master Protein] ATP synthase F(0) complex subunit B1, mitochondrial OS=Homo sapiens OX=9606 GN=ATP5PB PE=1 SV=2 |
| 150 | 5 | 1 | 1 | 1 | [Master Protein] Phosphoglucomutase-1 OS=Homo sapiens OX=9606 GN=PGM1 PE=1 SV=1 |
| 698 | 2 | 1 | 1 | 1 | [Master Protein] cDNA FLJ51625, highly similar to Ubiquinol-cytochrome-c reductase complex coreprotein I, mitochondrial OS=Homo sapiens OX=9606 PE=2 SV=1 |
| 101 | 8 | 1 | 1 | 1 | [Master Protein] Trafficking protein particle complex subunit 8 OS=Homo sapiens OX=9606 GN=TRAPPC8 PE=1 SV=1 |
| 110 | 2 | 1 | 1 | 1 | [Master Protein] 60S ribosomal protein L13 OS=Homo sapiens OX=9606 GN=RPL13 PE=1 SV=4 |
| 700 | 2 | 1 | 1 | 1 | [Master Protein] Nucleoporin NUP35 OS=Homo sapiens OX=9606 GN=NUP35 PE=1 SV=1 |
| 152 | 3 | 1 | 1 | 1 | [Master Protein] Alpha-soluble NSF attachment protein (Fragment) OS=Homo sapiens OX=9606 GN=NAPA PE=1 SV=1 |
| 102 | 4 | 1 | 1 | 1 | [Master Protein] Immunoglobulin J chain (Fragment) OS=Homo sapiens OX=9606 GN=JCHAIN PE=1 SV=1 |
| 710 | 1 | 1 | 1 | 1 | [Master Protein] Brain acid soluble protein 1 OS=Homo sapiens OX=9606 GN=BASP1 PE=1 SV=2 |
| 642 | 2 | 1 | 1 | 1 | [Master Protein] Protein MTO1 homolog, mitochondrial OS=Homo sapiens OX=9606 GN=MTO1 PE=1 SV=2 |
| 157 | 18 | 1 | 1 | 1 | [Master Protein] Putative protein FAM90A13P OS=Homo sapiens OX=9606 GN=FAM90A13P PE=5 SV=1 |
| 107 | 25 | 1 | 1 | 1 | [Master Protein] Disks large homolog 1 OS=Homo sapiens OX=9606 GN=DLG1 PE=1 SV=1 |
| 637 | 6 | 1 | 1 | 1 | [Master Protein] Rho GDP-dissociation inhibitor 2 (Fragment) OS=Homo sapiens OX=9606 GN=ARHGDIB PE=1 SV=1 |
| 154 | 3 | 1 | 1 | 1 | [Master Protein] Prominin-2 OS=Homo sapiens OX=9606 GN=PROM2 PE=1 SV=1 |
| 707 | 1 | 1 | 1 | 1 | [Master Protein] Membrane cofactor protein (Fragment) OS=Homo sapiens OX=9606 GN=CD46 PE=1 SV=1 |
| 155 | 8 | 1 | 1 | 1 | [Master Protein] Deoxynucleoside triphosphate triphosphohydrolase SAMHD1 OS=Homo sapiens OX=9606 GN=SAMHD1 PE=1 SV=1 |
| 697 | 2 | 1 | 1 | 1 | [Master Protein] COMM domain-containing protein 9 OS=Homo sapiens OX=9606 GN=COMMD9 PE=1 SV=2 |
| 156 | 4 | 1 | 1 | 1 | [Master Protein] PDHA1/LOC79064 protein (Fragment) OS=Homo sapiens OX=9606 GN=PDHA1/LOC79064 PE=2 SV=1 |
| 103 | 3 | 1 | 1 | 1 | [Master Protein] DNA-(apurinic or apyrimidinic site) endonuclease OS=Homo sapiens OX=9606 GN=APEX1 PE=2 SV=1 |
| 709 | 2 | 1 | 1 | 1 | [Master Protein] Cation-independent mannose-6-phosphate receptor OS=Homo sapiens OX=9606 GN=IGF2R PE=1 SV=3 |
| 705 | 6 | 1 | 1 | 1 | [Master Protein] 5'-nucleotidase (Fragment) OS=Homo sapiens OX=9606 GN=NT5E PE=1 SV=1 |
| 711 | 1 | 1 | 1 | 1 | [Master Protein] Torsin-1A-interacting protein 2 OS=Homo sapiens OX=9606 GN=TOR1AIP2 PE=1 SV=1 |
| 689 | 1 | 1 | 1 | 1 | [Master Protein] IGH + IGL c476_heavy_IGHV3-74_IGHD3-10_IGHJ4 (Fragment) OS=Homo sapiens OX=9606 PE=2 SV=1 |
| 695 | 2 | 1 | 1 | 1 | [Master Protein] HCG2044799 OS=Homo sapiens OX=9606 GN=HNRNPUL2-BSCL2 PE=4 SV=1 |
| 129 | 1 | 1 | 1 | 1 | [Master Protein] DING protein p38 OS=Homo sapiens OX=9606 PE=3 SV=1 |
| 677 | 2 | 1 | 1 | 1 | [Master Protein] Charged multivesicular body protein 6 (Fragment) OS=Homo sapiens OX=9606 GN=CHMP6 PE=1 SV=1 |
| 131 | 7 | 1 | 1 | 1 | [Master Protein] Tapasin OS=Homo sapiens OX=9606 GN=TAPBP PE=1 SV=1 |
| 656 | 4 | 1 | 1 | 1 | [Master Protein] Anoctamin-6 OS=Homo sapiens OX=9606 GN=ANO6 PE=1 SV=2 |
| 657 | 3 | 1 | 1 | 1 | [Master Protein] Voltage-dependent anion-selective channel protein 1 OS=Homo sapiens OX=9606 PE=2 SV=1 |
| 675 | 1 | 1 | 1 | 1 | [Master Protein] Sialate O-acetylesterase OS=Homo sapiens OX=9606 GN=SIAE PE=1 SV=1 |
| 674 | 1 | 1 | 1 | 1 | [Master Protein] IGL c3325_light_IGLV7-46_IGLJ3 (Fragment) OS=Homo sapiens OX=9606 PE=2 SV=1 |
| 143 | 9 | 1 | 1 | 1 | [Master Protein] Charged multivesicular body protein 1a OS=Homo sapiens OX=9606 GN=CHMP1A PE=1 SV=1 |
| 679 | 3 | 1 | 1 | 1 | [Master Protein] Hyaluronoglucosaminidase OS=Homo sapiens OX=9606 PE=2 SV=1 |
| 138 | 2 | 1 | 1 | 1 | [Master Protein] X-ray repair cross-complementing protein 5 OS=Homo sapiens OX=9606 GN=XRCC5 PE=1 SV=3 |
| 132 | 3 | 1 | 1 | 1 | [Master Protein] Cleft lip and palate transmembrane protein 1 (Fragment) OS=Homo sapiens OX=9606 GN=CLPTM1 PE=1 SV=1 |
| 662 | 1 | 1 | 1 | 1 | [Master Protein] Inactive N-acetylated-alpha-linked acidic dipeptidase-like protein 2 OS=Homo sapiens OX=9606 GN=NAALADL2 PE=1 SV=3 |
| 664 | 9 | 1 | 1 | 1 | [Master Protein] Golgi reassembly-stacking protein 1 OS=Homo sapiens OX=9606 GN=GORASP1 PE=1 SV=3 |
| 133 | 2 | 1 | 1 | 1 | [Master Protein] Heterogeneous nuclear ribonucleoprotein A2/B1, isoform CRA_d OS=Homo sapiens OX=9606 GN=HNRPA2B1 PE=4 SV=1 |
| 134 | 32 | 1 | 1 | 1 | [Master Protein] IGL c3320_light_IGKV1-5_IGKJ1 (Fragment) OS=Homo sapiens OX=9606 PE=2 SV=1 |
| 669 | 1 | 1 | 1 | 1 | [Master Protein] Testicular tissue protein Li 227 OS=Homo sapiens OX=9606 PE=2 SV=1 |
| 668 | 1 | 1 | 1 | 1 | [Master Protein] Unconventional myosin-Vb OS=Homo sapiens OX=9606 GN=MYO5B PE=1 SV=3 |
| 665 | 1 | 1 | 1 | 1 | [Master Protein] IGH c427_heavy__IGHV3-74_IGHD7-27_IGHJ4 (Fragment) OS=Homo sapiens OX=9606 PE=2 SV=1 |
| 673 | 5 | 1 | 1 | 1 | [Master Protein] 40S ribosomal protein S15a (Fragment) OS=Homo sapiens OX=9606 PE=3 SV=2 |
| 117 | 4 | 1 | 1 | 1 | [Master Protein] Synaptophysin-like protein 1 (Fragment) OS=Homo sapiens OX=9606 GN=SYPL1 PE=1 SV=1 |
| 145 | 5 | 1 | 1 | 1 | [Master Protein] Alkyldihydroxyacetonephosphate synthase, peroxisomal OS=Homo sapiens OX=9606 GN=AGPS PE=1 SV=1 |
| 680 | 2 | 1 | 1 | 1 | [Master Protein] cDNA FLJ59669, highly similar to Homo sapiens phospholipase D family, member 3 (PLD3), transcript variant 1, mRNA OS=Homo sapiens OX=9606 PE=2 SV=1 |
| 694 | 2 | 1 | 1 | 1 | [Master Protein] cDNA FLJ41755 fis, clone HSYRA2009102, highly similar to Adenosine 3'-phospho 5'-phosphosulfate transporter 1 OS=Homo sapiens OX=9606 PE=2 SV=1 |
| 118 | 6 | 1 | 1 | 1 | [Master Protein] 26S proteasome non-ATPase regulatory subunit 8 OS=Homo sapiens OX=9606 GN=PSMD8 PE=1 SV=2 |
| 148 | 2 | 1 | 1 | 1 | [Master Protein] Protein XRP2 OS=Homo sapiens OX=9606 GN=RP2 PE=1 SV=4 |
| 119 | 11 | 1 | 1 | 1 | [Master Protein] Biglycan OS=Homo sapiens OX=9606 PE=2 SV=1 |
| 121 | 7 | 1 | 1 | 1 | [Master Protein] VPS35 endosomal protein-sorting factor-like OS=Homo sapiens OX=9606 GN=VPS35L PE=1 SV=2 |
| 123 | 9 | 1 | 1 | 1 | [Master Protein] Serine/threonine-protein phosphatase OS=Homo sapiens OX=9606 GN=PPP3CB PE=1 SV=1 |
| 648 | 3 | 1 | 1 | 1 | [Master Protein] Mitogen-activated protein kinase OS=Homo sapiens OX=9606 GN=MAPK1 PE=3 SV=1 |
| 688 | 3 | 1 | 1 | 1 | [Master Protein] IGH + IGL c180_light_IGKV3-15_IGKJ3 (Fragment) OS=Homo sapiens OX=9606 PE=2 SV=1 |
| 654 | 4 | 1 | 1 | 1 | [Master Protein] Aminopeptidase B OS=Homo sapiens OX=9606 GN=RNPEP PE=3 SV=1 |
| 687 | 5 | 1 | 1 | 1 | [Master Protein] Sphingomyelin phosphodiesterase 3 OS=Homo sapiens OX=9606 GN=SMPD3 PE=1 SV=1 |
| 125 | 5 | 1 | 1 | 1 | [Master Protein] Heterogeneous nuclear ribonucleoprotein K OS=Homo sapiens OX=9606 PE=2 SV=1 |
| 650 | 4 | 1 | 1 | 1 | [Master Protein] Eukaryotic translation initiation factor 3 subunit F OS=Homo sapiens OX=9606 GN=EIF3F PE=2 SV=1 |
| 126 | 4 | 1 | 1 | 1 | [Master Protein] Phenylalanine--tRNA ligase alpha subunit OS=Homo sapiens OX=9606 GN=FARSA PE=1 SV=1 |
| 683 | 8 | 1 | 1 | 1 | [Master Protein] Ras-related protein Rab-7a OS=Homo sapiens OX=9606 GN=RAB7A PE=1 SV=1 |
| 128 | 5 | 1 | 1 | 1 | [Master Protein] T-complex protein 1 subunit gamma (Fragment) OS=Homo sapiens OX=9606 PE=2 SV=1 |
| 651 | 2 | 1 | 1 | 1 | [Master Protein] Lysophosphatidylserine lipase ABHD12 OS=Homo sapiens OX=9606 GN=ABHD12 PE=1 SV=2 |
| 652 | 5 | 1 | 1 | 1 | [Master Protein] Malate dehydrogenase (Fragment) OS=Homo sapiens OX=9606 GN=MDH1 PE=1 SV=1 |
| 146 | 9 | 1 | 1 | 1 | [Master Protein] Caveolin (Fragment) OS=Homo sapiens OX=9606 PE=2 SV=1 |
| 124 | 7 | 1 | 1 | 1 | [Master Protein] Signal sequence receptor subunit alpha OS=Homo sapiens OX=9606 GN=SSR1 PE=1 SV=1 |
| 666 | 3 | 1 | 1 | 1 | [Master Protein] Palladin (Fragment) OS=Homo sapiens OX=9606 GN=PALLD PE=1 SV=1 |
| 79 | 3 | 1 | 1 | 1 | [Master Protein] Clathrin light chain OS=Homo sapiens OX=9606 GN=CLTB PE=3 SV=1 |
| 93 | 3 | 1 | 1 | 1 | [Master Protein] Cytosol aminopeptidase OS=Homo sapiens OX=9606 GN=LAP3 PE=1 SV=3 |
| 28 | 2 | 1 | 1 | 1 | [Master Protein] 2,4-dienoyl-CoA reductase, mitochondrial (Fragment) OS=Homo sapiens OX=9606 GN=DECR1 PE=1 SV=1 |
| 818 | 1 | 1 | 1 | 1 | [Master Protein] Leucine-rich repeat-containing protein 47 OS=Homo sapiens OX=9606 GN=LRRC47 PE=1 SV=1 |
| 817 | 1 | 1 | 1 | 1 | [Master Protein] Protein FAM98B OS=Homo sapiens OX=9606 GN=FAM98B PE=1 SV=2 |
| 30 | 5 | 1 | 1 | 1 | [Master Protein] cDNA FLJ33764 fis, clone BRCOC2000360, highly similar to Vacuolar ATP synthase subunit S1 OS=Homo sapiens OX=9606 PE=2 SV=1 |
| 31 | 4 | 1 | 1 | 1 | [Master Protein] Elongation factor 1-gamma OS=Homo sapiens OX=9606 GN=EEF1G PE=1 SV=3 |
| 35 | 7 | 1 | 1 | 1 | [Master Protein] Alcohol dehydrogenase 1C (Fragment) OS=Homo sapiens OX=9606 GN=ADH1C PE=1 SV=1 |
| 36 | 1 | 1 | 1 | 1 | [Master Protein] Calcineurin B homologous protein 2 OS=Homo sapiens OX=9606 GN=CHP2 PE=1 SV=3 |
| 811 | 2 | 1 | 1 | 1 | [Master Protein] Splicing factor U2AF 35 kDa subunit OS=Homo sapiens OX=9606 GN=U2AF1 PE=1 SV=3 |
| 810 | 1 | 1 | 1 | 1 | [Master Protein] 3-mercaptopyruvate sulfurtransferase OS=Homo sapiens OX=9606 GN=MPST PE=1 SV=3 |
| 38 | 11 | 1 | 1 | 1 | [Master Protein] IG c1497_light_IGKV3-20_IGKJ1 (Fragment) OS=Homo sapiens OX=9606 PE=2 SV=1 |
| 40 | 12 | 1 | 1 | 1 | [Master Protein] Glucosylceramidase OS=Homo sapiens OX=9606 GN=GBA PE=1 SV=1 |
| 25 | 11 | 1 | 1 | 1 | [Master Protein] cDNA FLJ55918, highly similar to Echinoderm microtubule-associated protein-like 2 OS=Homo sapiens OX=9606 PE=2 SV=1 |
| 806 | 1 | 1 | 1 | 1 | [Master Protein] Polyadenylate-binding protein 2 OS=Homo sapiens OX=9606 GN=PABPN1 PE=1 SV=3 |
| 804 | 1 | 1 | 1 | 1 | [Master Protein] CD2-associated protein OS=Homo sapiens OX=9606 GN=CD2AP PE=1 SV=1 |
| 41 | 17 | 1 | 1 | 1 | [Master Protein] BCR/ABL fusion protein isoform Y2 OS=Homo sapiens OX=9606 GN=BCR/ABL fusion PE=2 SV=1 |
| 42 | 4 | 1 | 1 | 1 | [Master Protein] Cullin-associated NEDD8-dissociated protein 1 (Fragment) OS=Homo sapiens OX=9606 GN=CAND1 PE=1 SV=1 |
| 43 | 42 | 1 | 1 | 1 | [Master Protein] IGH + IGL c426_light_IGKV3-20_IGKJ3 (Fragment) OS=Homo sapiens OX=9606 PE=2 SV=1 |
| 800 | 3 | 1 | 1 | 1 | [Master Protein] Collagen alpha-1(XIV) chain (Fragment) OS=Homo sapiens OX=9606 GN=COL14A1 PE=1 SV=1 |
| 798 | 1 | 1 | 1 | 1 | [Master Protein] MHC class II antigen (Fragment) OS=Homo sapiens OX=9606 GN=HLA-DRB1 PE=4 SV=1 |
| 797 | 2 | 1 | 1 | 1 | [Master Protein] Golgi integral membrane protein 4 OS=Homo sapiens OX=9606 GN=GOLIM4 PE=1 SV=1 |
| 44 | 5 | 1 | 1 | 1 | [Master Protein] Intercellular adhesion molecule 3 (Fragment) OS=Homo sapiens OX=9606 GN=ICAM3 PE=1 SV=8 |
| 793 | 2 | 1 | 1 | 1 | [Master Protein] Leukocyte elastase inhibitor OS=Homo sapiens OX=9606 GN=SERPINB1 PE=1 SV=1 |
| 47 | 9 | 1 | 1 | 1 | [Master Protein] Heterogeneous nuclear ribonucleoprotein A/B OS=Homo sapiens OX=9606 GN=HNRNPAB PE=1 SV=2 |
| 791 | 4 | 1 | 1 | 1 | [Master Protein] ATP synthase subunit O, mitochondrial OS=Homo sapiens OX=9606 GN=ATP5PO PE=1 SV=1 |
| 805 | 1 | 1 | 1 | 1 | [Master Protein] Actin-related protein 2/3 complex subunit OS=Homo sapiens OX=9606 GN=ARPC1B PE=2 SV=1 |
| 49 | 7 | 1 | 1 | 1 | [Master Protein] Secretory carrier-associated membrane protein OS=Homo sapiens OX=9606 PE=2 SV=1 |
| 822 | 1 | 1 | 1 | 1 | [Master Protein] Peroxisomal biogenesis factor 11B, isoform CRA_a OS=Homo sapiens OX=9606 GN=PEX11B PE=4 SV=1 |
| 824 | 1 | 1 | 1 | 1 | [Master Protein] Peroxisomal bifunctional enzyme OS=Homo sapiens OX=9606 GN=EHHADH PE=1 SV=3 |
| 855 | 2 | 1 | 1 | 1 | [Master Protein] Golgi phosphoprotein 3-like OS=Homo sapiens OX=9606 GN=GOLPH3L PE=1 SV=1 |
| 2 | 5 | 1 | 1 | 1 | [Master Protein] Tetraspanin (Fragment) OS=Homo sapiens OX=9606 GN=CD63 PE=2 SV=1 |
| 853 | 1 | 1 | 1 | 1 | [Master Protein] Lipase maturation factor 2 OS=Homo sapiens OX=9606 GN=LMF2 PE=1 SV=2 |
| 850 | 1 | 1 | 1 | 1 | [Master Protein] Filamin-C OS=Homo sapiens OX=9606 GN=FLNC PE=1 SV=3 |
| 5 | 5 | 1 | 1 | 1 | [Master Protein] Cytoplasmic dynein 1 heavy chain 1 (Fragment) OS=Homo sapiens OX=9606 GN=DYNC1H1 PE=1 SV=1 |
| 848 | 1 | 1 | 1 | 1 | [Master Protein] Acyl-Coenzyme A binding domain containing 3, isoform CRA_a OS=Homo sapiens OX=9606 GN=ACBD3 PE=4 SV=1 |
| 7 | 3 | 1 | 1 | 1 | [Master Protein] Probable cytosolic iron-sulfur protein assembly protein CIAO1 OS=Homo sapiens OX=9606 GN=CIAO1 PE=1 SV=1 |
| 8 | 9 | 1 | 1 | 1 | [Master Protein] NAD(P) transhydrogenase, mitochondrial OS=Homo sapiens OX=9606 GN=NNT PE=1 SV=3 |
| 845 | 1 | 1 | 1 | 1 | [Master Protein] Testis tissue sperm-binding protein Li 39a OS=Homo sapiens OX=9606 PE=2 SV=1 |
| 9 | 4 | 1 | 1 | 1 | [Master Protein] Protein kinase C and casein kinase substrate in neurons 2, isoform CRA_a OS=Homo sapiens OX=9606 GN=PACSIN2 PE=4 SV=1 |
| 10 | 9 | 1 | 1 | 1 | [Master Protein] cDNA FLJ32530 fis, clone SMINT2000185, highly similar to TRANSKETOLASE OS=Homo sapiens OX=9606 PE=2 SV=1 |
| 823 | 1 | 1 | 1 | 1 | [Master Protein] Emopamil binding protein OS=Homo sapiens OX=9606 GN=EBP PE=2 SV=1 |
| 11 | 4 | 1 | 1 | 1 | [Master Protein] Fibrillin 1 OS=Homo sapiens OX=9606 GN=FBN1 PE=3 SV=1 |
| 839 | 1 | 1 | 1 | 1 | [Master Protein] Disintegrin and metalloproteinase domain-containing protein 10 OS=Homo sapiens OX=9606 GN=ADAM10 PE=1 SV=1 |
| 838 | 1 | 1 | 1 | 1 | [Master Protein] Fatty acid-binding protein 5 OS=Homo sapiens OX=9606 GN=FABP5 PE=1 SV=3 |
| 15 | 1 | 1 | 1 | 1 | [Master Protein] Ubiquitin carboxyl-terminal hydrolase OS=Homo sapiens OX=9606 PE=2 SV=1 |
| 836 | 1 | 1 | 1 | 1 | [Master Protein] Protein MAL2 OS=Homo sapiens OX=9606 GN=MAL2 PE=1 SV=1 |
| 18 | 7 | 1 | 1 | 1 | [Master Protein] Cytochrome b-c1 complex subunit 2, mitochondrial OS=Homo sapiens OX=9606 GN=UQCRC2 PE=1 SV=1 |
| 831 | 1 | 1 | 1 | 1 | [Master Protein] Protein POF1B OS=Homo sapiens OX=9606 GN=POF1B PE=1 SV=3 |
| 23 | 7 | 1 | 1 | 1 | [Master Protein] cDNA FLJ56054, highly similar to 26S proteasome non-ATPase regulatory subunit 3 OS=Homo sapiens OX=9606 PE=2 SV=1 |
| 24 | 15 | 1 | 1 | 1 | [Master Protein] Septin-7 OS=Homo sapiens OX=9606 GN=SEPT7 PE=3 SV=1 |
| 828 | 2 | 1 | 1 | 1 | [Master Protein] Mitochondrial 2-oxoglutarate/malate carrier protein OS=Homo sapiens OX=9606 GN=SLC25A11 PE=1 SV=3 |
| 827 | 2 | 1 | 1 | 1 | [Master Protein] Sialoadhesin OS=Homo sapiens OX=9606 GN=SIGLEC1 PE=1 SV=2 |
| 826 | 2 | 1 | 1 | 1 | [Master Protein] Quinone oxidoreductase PIG3 OS=Homo sapiens OX=9606 GN=TP53I3 PE=1 SV=2 |
| 12 | 22 | 1 | 1 | 1 | [Master Protein] MHC class I antigen (Fragment) OS=Homo sapiens OX=9606 GN=HLA-E PE=3 SV=1 |
| 720 | 2 | 1 | 1 | 1 | [Master Protein] FEX1 OS=Homo sapiens OX=9606 PE=2 SV=1 |
| 788 | 1 | 1 | 1 | 1 | [Master Protein] IGL c1156_light_IGKV3-20_IGKJ2 (Fragment) OS=Homo sapiens OX=9606 PE=2 SV=1 |
| 50 | 68 | 1 | 1 | 1 | [Master Protein] IG c681_heavy_IGHV3-9_IGHD6-19_IGHJ4 (Fragment) OS=Homo sapiens OX=9606 PE=2 SV=1 |
| 74 | 7 | 1 | 1 | 1 | [Master Protein] Tyrosine-protein kinase (Fragment) OS=Homo sapiens OX=9606 GN=JAK1 PE=1 SV=1 |
| 751 | 2 | 1 | 1 | 1 | [Master Protein] Heterogeneous nuclear ribonucleoprotein F OS=Homo sapiens OX=9606 GN=HNRNPF PE=1 SV=3 |
| 749 | 3 | 1 | 1 | 1 | [Master Protein] Chromosome 6 open reading frame 55, isoform CRA_b OS=Homo sapiens OX=9606 GN=VTA1 PE=1 SV=1 |
| 748 | 3 | 1 | 1 | 1 | [Master Protein] Protein transport protein Sec24B OS=Homo sapiens OX=9606 GN=SEC24B PE=1 SV=2 |
| 75 | 6 | 1 | 1 | 1 | [Master Protein] Vigilin OS=Homo sapiens OX=9606 GN=HDLBP PE=1 SV=2 |
| 77 | 4 | 1 | 1 | 1 | [Master Protein] Methyltransferase-like protein 7A (Fragment) OS=Homo sapiens OX=9606 GN=METTL7A PE=1 SV=1 |
| 745 | 1 | 1 | 1 | 1 | [Master Protein] Epididymis secretory protein Li 112 OS=Homo sapiens OX=9606 GN=HEL-S-112 PE=2 SV=1 |
| 78 | 38 | 1 | 1 | 1 | [Master Protein] Beta globin (Fragment) OS=Homo sapiens OX=9606 GN=HBB PE=3 SV=1 |
| 743 | 4 | 1 | 1 | 1 | [Master Protein] ATP-citrate synthase (Fragment) OS=Homo sapiens OX=9606 GN=ACLY PE=1 SV=1 |
| 633 | 3 | 1 | 1 | 1 | [Master Protein] GPI-anchor transamidase OS=Homo sapiens OX=9606 GN=PIGK PE=1 SV=2 |
| 80 | 5 | 1 | 1 | 1 | [Master Protein] Amino acid transporter OS=Homo sapiens OX=9606 GN=SLC1A5 PE=1 SV=1 |
| 71 | 3 | 1 | 1 | 1 | [Master Protein] Mitochondrial aldehyde dehydrogenase 2 variant (Fragment) OS=Homo sapiens OX=9606 PE=2 SV=1 |
| 82 | 6 | 1 | 1 | 1 | [Master Protein] Monocarboxylate transporter 1 (Fragment) OS=Homo sapiens OX=9606 GN=SLC16A1 PE=1 SV=1 |
| 735 | 2 | 1 | 1 | 1 | [Master Protein] Arginase-1 OS=Homo sapiens OX=9606 GN=ARG1 PE=1 SV=2 |
| 734 | 2 | 1 | 1 | 1 | [Master Protein] cDNA FLJ53108, highly similar to Guanine nucleotide-binding protein alpha-13 subunit OS=Homo sapiens OX=9606 PE=2 SV=1 |
| 732 | 2 | 1 | 1 | 1 | [Master Protein] GDP-mannose 4,6 dehydratase OS=Homo sapiens OX=9606 GN=GMDS PE=1 SV=1 |
| 731 | 1 | 1 | 1 | 1 | [Master Protein] Vacuolar protein sorting-associated protein 29 OS=Homo sapiens OX=9606 GN=VPS29 PE=1 SV=1 |
| 87 | 11 | 1 | 1 | 1 | [Master Protein] Septin-9 (Fragment) OS=Homo sapiens OX=9606 GN=SEPTIN9 PE=1 SV=1 |
| 729 | 1 | 1 | 1 | 1 | [Master Protein] Glycerol-3-phosphate phosphatase OS=Homo sapiens OX=9606 GN=PGP PE=1 SV=1 |
| 728 | 2 | 1 | 1 | 1 | [Master Protein] Ribosome maturation protein SBDS OS=Homo sapiens OX=9606 GN=SBDS PE=1 SV=4 |
| 726 | 3 | 1 | 1 | 1 | [Master Protein] Unconventional myosin-VIIa OS=Homo sapiens OX=9606 GN=MYO7A PE=1 SV=2 |
| 725 | 1 | 1 | 1 | 1 | [Master Protein] Mannosyl-oligosaccharide 1,2-alpha-mannosidase IA OS=Homo sapiens OX=9606 GN=MAN1A1 PE=1 SV=3 |
| 91 | 5 | 1 | 1 | 1 | [Master Protein] Transferrin receptor protein 1 OS=Homo sapiens OX=9606 GN=TFRC PE=1 SV=1 |
| 723 | 1 | 1 | 1 | 1 | [Master Protein] Dehydrogenase/reductase SDR family member 11 OS=Homo sapiens OX=9606 GN=DHRS11 PE=1 SV=1 |
| 737 | 3 | 1 | 1 | 1 | [Master Protein] Cytochrome b-245 heavy chain OS=Homo sapiens OX=9606 GN=CYBB PE=1 SV=2 |
| 787 | 3 | 1 | 1 | 1 | [Master Protein] Guanine nucleotide-binding protein G(q) subunit alpha (Fragment) OS=Homo sapiens OX=9606 GN=GNAQ PE=1 SV=1 |
| 70 | 7 | 1 | 1 | 1 | [Master Protein] Apolipoprotein E (Fragment) OS=Homo sapiens OX=9606 GN=APOE PE=1 SV=1 |
| 68 | 2 | 1 | 1 | 1 | [Master Protein] Cleavage stimulation factor subunit 3 OS=Homo sapiens OX=9606 GN=CSTF3 PE=1 SV=1 |
| 52 | 8 | 1 | 1 | 1 | [Master Protein] Tetraspanin OS=Homo sapiens OX=9606 PE=2 SV=1 |
| 53 | 2 | 1 | 1 | 1 | [Master Protein] MHC class I antigen (Fragment) OS=Homo sapiens OX=9606 GN=HLA-B PE=3 SV=1 |
| 783 | 1 | 1 | 1 | 1 | [Master Protein] Pyruvate dehydrogenase E1 component subunit beta OS=Homo sapiens OX=9606 PE=2 SV=1 |
| 782 | 3 | 1 | 1 | 1 | [Master Protein] Leukocyte surface antigen CD47 OS=Homo sapiens OX=9606 GN=CD47 PE=1 SV=1 |
| 55 | 3 | 1 | 1 | 1 | [Master Protein] cAMP-dependent protein kinase type I-alpha regulatory subunit OS=Homo sapiens OX=9606 GN=PRKAR1A PE=1 SV=1 |
| 56 | 5 | 1 | 1 | 1 | [Master Protein] NAD+-dependent 15-hydroxyprostaglandin dehydrogenase (Fragment) OS=Homo sapiens OX=9606 GN=PGDH PE=4 SV=1 |
| 58 | 5 | 1 | 1 | 1 | [Master Protein] IG c655_light_IGKV3-20_IGKJ3 (Fragment) OS=Homo sapiens OX=9606 PE=2 SV=1 |
| 775 | 1 | 1 | 1 | 1 | [Master Protein] Protein Niban 1 OS=Homo sapiens OX=9606 GN=NIBAN1 PE=1 SV=1 |
| 60 | 4 | 1 | 1 | 1 | [Master Protein] cDNA FLJ77864, highly similar to Homo sapiens HSNFRK (HSNFRK) mRNA OS=Homo sapiens OX=9606 PE=2 SV=1 |
| 773 | 1 | 1 | 1 | 1 | [Master Protein] Golgin subfamily A member 3 OS=Homo sapiens OX=9606 GN=GOLGA3 PE=1 SV=2 |
| 772 | 3 | 1 | 1 | 1 | [Master Protein] Carbonyl reductase [NADPH] 1 OS=Homo sapiens OX=9606 GN=CBR1 PE=1 SV=3 |
| 69 | 4 | 1 | 1 | 1 | [Master Protein] cDNA, FLJ92775, highly similar to Homo sapiens melanoma cell adhesion molecule (MCAM), mRNA OS=Homo sapiens OX=9606 PE=2 SV=1 |
| 770 | 2 | 1 | 1 | 1 | [Master Protein] Chromatin modifying protein 2B OS=Homo sapiens OX=9606 GN=CHMP2B PE=2 SV=1 |
| 768 | 3 | 1 | 1 | 1 | [Master Protein] MAGUK p55 subfamily member 7 OS=Homo sapiens OX=9606 PE=2 SV=1 |
| 767 | 3 | 1 | 1 | 1 | [Master Protein] Protein tyrosine phosphatase type IVA 1 OS=Homo sapiens OX=9606 GN=PTP4A1 PE=1 SV=1 |
| 63 | 10 | 1 | 1 | 1 | [Master Protein] Chitinase domain-containing protein 1 (Fragment) OS=Homo sapiens OX=9606 GN=CHID1 PE=1 SV=1 |
| 64 | 8 | 1 | 1 | 1 | [Master Protein] Dipeptidyl peptidase 3 (Fragment) OS=Homo sapiens OX=9606 GN=DPP3 PE=1 SV=1 |
| 764 | 1 | 1 | 1 | 1 | [Master Protein] MAJIN OS=Homo sapiens OX=9606 GN=MAJIN PE=2 SV=1 |
| 763 | 2 | 1 | 1 | 1 | [Master Protein] BRO1 domain-containing protein BROX OS=Homo sapiens OX=9606 GN=BROX PE=1 SV=1 |
| 762 | 2 | 1 | 1 | 1 | [Master Protein] Integrin alpha-L OS=Homo sapiens OX=9606 GN=ITGAL PE=1 SV=3 |
| 65 | 12 | 1 | 1 | 1 | [Master Protein] FAS variant OS=Homo sapiens OX=9606 GN=FAS PE=2 SV=1 |
| 760 | 3 | 1 | 1 | 1 | [Master Protein] Catalase OS=Homo sapiens OX=9606 PE=2 SV=1 |
| 66 | 2 | 1 | 1 | 1 | [Master Protein] cDNA FLJ75348, highly similar to Homo sapiens metabotropic glutamate receptor 8b OS=Homo sapiens OX=9606 PE=2 SV=1 |
| 67 | 11 | 1 | 1 | 1 | [Master Protein] Monocarboxylate transporter 4 (Fragment) OS=Homo sapiens OX=9606 GN=SLC16A3 PE=1 SV=1 |
| 62 | 7 | 1 | 1 | 1 | [Master Protein] V-type proton ATPase subunit OS=Homo sapiens OX=9606 GN=ATP6V0D1 PE=1 SV=1 |
| 632 | 4 | 1 | 1 | 1 | [Master Protein] Huntingtin-interacting protein 1-related protein OS=Homo sapiens OX=9606 GN=HIP1R PE=1 SV=2 |
| 338 | 3 | 1 | 1 | 1 | [Master Protein] TJP3 protein (Fragment) OS=Homo sapiens OX=9606 GN=TJP3 PE=2 SV=2 |
| 164 | 4 | 1 | 1 | 1 | [Master Protein] Alpha-2-HS-glycoprotein OS=Homo sapiens OX=9606 PE=2 SV=1 |
| 279 | 13 | 1 | 1 | 1 | [Master Protein] Desmocollin 3 (Fragment) OS=Homo sapiens OX=9606 GN=DSC3 PE=2 SV=1 |
| 443 | 5 | 1 | 1 | 1 | [Master Protein] Inactive tyrosine-protein kinase 7 OS=Homo sapiens OX=9606 GN=PTK7 PE=1 SV=1 |
| 441 | 6 | 1 | 1 | 1 | [Master Protein] Peptidylprolyl isomerase OS=Homo sapiens OX=9606 PE=2 SV=1 |
| 440 | 4 | 1 | 1 | 1 | [Master Protein] Carcinoembryonic antigen-related cell adhesion molecule 1 OS=Homo sapiens OX=9606 GN=CEACAM1 PE=1 SV=2 |
| 439 | 3 | 1 | 1 | 1 | [Master Protein] Prolargin OS=Homo sapiens OX=9606 GN=PRELP PE=1 SV=1 |
| 438 | 2 | 1 | 1 | 1 | [Master Protein] Ras-related protein Ral-A OS=Homo sapiens OX=9606 GN=RALA PE=1 SV=1 |
| 437 | 5 | 1 | 1 | 1 | [Master Protein] Vesicle transport through interaction with t-SNAREs homolog 1B OS=Homo sapiens OX=9606 GN=VTI1B PE=1 SV=3 |
| 436 | 4 | 1 | 1 | 1 | [Master Protein] Putative RNA-binding protein Luc7-like 2 OS=Homo sapiens OX=9606 GN=LUC7L2 PE=1 SV=2 |
| 435 | 1 | 1 | 1 | 1 | [Master Protein] Epididymis secretory sperm binding protein OS=Homo sapiens OX=9606 GN=PEF1 PE=2 SV=1 |
| 282 | 3 | 1 | 1 | 1 | [Master Protein] Succinyl-CoA:3-ketoacid CoA transferase (Fragment) OS=Homo sapiens OX=9606 GN=OXCT1 PE=2 SV=1 |
| 432 | 7 | 1 | 1 | 1 | [Master Protein] Solute carrier family 52, riboflavin transporter, member 2 OS=Homo sapiens OX=9606 GN=SLC52A2 PE=1 SV=1 |
| 276 | 8 | 1 | 1 | 1 | [Master Protein] Collagen, type XI, alpha 1, isoform CRA_b OS=Homo sapiens OX=9606 GN=COL11A1 PE=4 SV=1 |
| 283 | 3 | 1 | 1 | 1 | [Master Protein] Splicing factor 3B subunit 3 OS=Homo sapiens OX=9606 GN=SF3B3 PE=1 SV=4 |
| 428 | 5 | 1 | 1 | 1 | [Master Protein] NCKAP1L protein (Fragment) OS=Homo sapiens OX=9606 GN=NCKAP1L PE=2 SV=2 |
| 287 | 7 | 1 | 1 | 1 | [Master Protein] Bifunctional epoxide hydrolase 2 OS=Homo sapiens OX=9606 GN=EPHX2 PE=1 SV=1 |
| 426 | 5 | 1 | 1 | 1 | [Master Protein] Squalene synthase (Fragment) OS=Homo sapiens OX=9606 GN=FDFT1 PE=1 SV=1 |
| 288 | 5 | 1 | 1 | 1 | [Master Protein] cDNA FLJ52530, highly similar to Tumor protein D54 OS=Homo sapiens OX=9606 PE=2 SV=1 |
| 424 | 1 | 1 | 1 | 1 | [Master Protein] Retinol dehydrogenase 14 OS=Homo sapiens OX=9606 GN=RDH14 PE=1 SV=1 |
| 422 | 2 | 1 | 1 | 1 | [Master Protein] Phospholipase A2 OS=Homo sapiens OX=9606 PE=2 SV=1 |
| 418 | 10 | 1 | 1 | 1 | [Master Protein] Vacuolar protein sorting-associated protein 45 OS=Homo sapiens OX=9606 GN=VPS45 PE=1 SV=1 |
| 417 | 2 | 1 | 1 | 1 | [Master Protein] Protein transport protein Sec24A OS=Homo sapiens OX=9606 GN=SEC24A PE=1 SV=2 |
| 416 | 2 | 1 | 1 | 1 | [Master Protein] cDNA FLJ52703, highly similar to Asparaginyl-tRNA synthetase, cytoplasmic (EC6.1.1.22) OS=Homo sapiens OX=9606 PE=2 SV=1 |
| 292 | 6 | 1 | 1 | 1 | [Master Protein] Tyrosine-protein kinase OS=Homo sapiens OX=9606 PE=2 SV=1 |
| 414 | 3 | 1 | 1 | 1 | [Master Protein] 40S ribosomal protein S6 OS=Homo sapiens OX=9606 GN=RPS6 PE=1 SV=1 |
| 284 | 8 | 1 | 1 | 1 | [Master Protein] Testis-expressed protein 264 (Fragment) OS=Homo sapiens OX=9606 GN=TEX264 PE=1 SV=1 |
| 413 | 4 | 1 | 1 | 1 | [Master Protein] 26S proteasome non-ATPase regulatory subunit 2 OS=Homo sapiens OX=9606 GN=PSMD2 PE=1 SV=3 |
| 448 | 5 | 1 | 1 | 1 | [Master Protein] cDNA FLJ75504, highly similar to Homo sapiens glucosidase I, mRNA OS=Homo sapiens OX=9606 PE=2 SV=1 |
| 453 | 2 | 1 | 1 | 1 | [Master Protein] Argininosuccinate synthase OS=Homo sapiens OX=9606 GN=ASS PE=2 SV=1 |
| 479 | 6 | 1 | 1 | 1 | [Master Protein] Xaa-Pro dipeptidase (Fragment) OS=Homo sapiens OX=9606 GN=PEPD PE=1 SV=3 |
| 253 | 5 | 1 | 1 | 1 | [Master Protein] UMP-CMP kinase OS=Homo sapiens OX=9606 GN=CMPK1 PE=1 SV=3 |
| 255 | 7 | 1 | 1 | 1 | [Master Protein] CMP-sialic acid transporter OS=Homo sapiens OX=9606 GN=SLC35A1 PE=1 SV=1 |
| 475 | 4 | 1 | 1 | 1 | [Master Protein] Coatomer subunit epsilon OS=Homo sapiens OX=9606 PE=2 SV=1 |
| 256 | 9 | 1 | 1 | 1 | [Master Protein] IST1 homolog (Fragment) OS=Homo sapiens OX=9606 GN=IST1 PE=1 SV=1 |
| 473 | 3 | 1 | 1 | 1 | [Master Protein] WD repeat-containing protein 5 OS=Homo sapiens OX=9606 GN=WDR5 PE=1 SV=1 |
| 258 | 4 | 1 | 1 | 1 | [Master Protein] Aldehyde dehydrogenase family 16 member A1 OS=Homo sapiens OX=9606 GN=ALDH16A1 PE=1 SV=2 |
| 259 | 4 | 1 | 1 | 1 | [Master Protein] Unconventional myosin-Ib OS=Homo sapiens OX=9606 GN=MYO1B PE=1 SV=1 |
| 470 | 3 | 1 | 1 | 1 | [Master Protein] Ectonucleoside triphosphate diphosphohydrolase 5 OS=Homo sapiens OX=9606 GN=ENTPD5 PE=1 SV=1 |
| 260 | 6 | 1 | 1 | 1 | [Master Protein] NF45 OS=Homo sapiens OX=9606 PE=2 SV=1 |
| 468 | 5 | 1 | 1 | 1 | [Master Protein] Beta-2-syntrophin (Fragment) OS=Homo sapiens OX=9606 GN=SNTB2 PE=1 SV=1 |
| 275 | 15 | 1 | 1 | 1 | [Master Protein] GTPase KRas OS=Homo sapiens OX=9606 GN=KRAS PE=1 SV=1 |
| 467 | 5 | 1 | 1 | 1 | [Master Protein] 60S ribosomal protein L5 OS=Homo sapiens OX=9606 GN=RPL5 PE=1 SV=1 |
| 465 | 12 | 1 | 1 | 1 | [Master Protein] N-myc downstream-regulated gene 1 protein OS=Homo sapiens OX=9606 GN=TRG14 PE=2 SV=1 |
| 464 | 4 | 1 | 1 | 1 | [Master Protein] Plastin-2 OS=Homo sapiens OX=9606 GN=LCP1 PE=1 SV=6 |
| 463 | 2 | 1 | 1 | 1 | [Master Protein] Epididymis secretory sperm binding protein OS=Homo sapiens OX=9606 GN=HPX PE=1 SV=1 |
| 267 | 5 | 1 | 1 | 1 | [Master Protein] Acylamino-acid-releasing enzyme OS=Homo sapiens OX=9606 GN=APEH PE=1 SV=4 |
| 268 | 1 | 1 | 1 | 1 | [Master Protein] Tissue alpha-L-fucosidase OS=Homo sapiens OX=9606 GN=FUCA1 PE=1 SV=4 |
| 270 | 3 | 1 | 1 | 1 | [Master Protein] 60S ribosomal protein L11 OS=Homo sapiens OX=9606 GN=RPL11 PE=1 SV=2 |
| 458 | 6 | 1 | 1 | 1 | [Master Protein] Nucleobindin-1 OS=Homo sapiens OX=9606 PE=2 SV=1 |
| 457 | 3 | 1 | 1 | 1 | [Master Protein] cDNA FLJ50778, highly similar to Protein flightless-1 homolog OS=Homo sapiens OX=9606 PE=2 SV=1 |
| 272 | 6 | 1 | 1 | 1 | [Master Protein] P2X purinoceptor 4 (Fragment) OS=Homo sapiens OX=9606 GN=P2RX4 PE=1 SV=1 |
| 273 | 6 | 1 | 1 | 1 | [Master Protein] Polypyrimidine tract-binding protein 1 OS=Homo sapiens OX=9606 GN=PTBP1 PE=1 SV=4 |
| 454 | 1 | 1 | 1 | 1 | [Master Protein] 60S ribosomal protein L34 OS=Homo sapiens OX=9606 GN=RPL34 PE=1 SV=3 |
| 261 | 4 | 1 | 1 | 1 | [Master Protein] Magnesium transporter protein 1 OS=Homo sapiens OX=9606 GN=MAGT1 PE=1 SV=1 |
| 480 | 2 | 1 | 1 | 1 | [Master Protein] Chromosome 9 open reading frame 88, isoform CRA_a OS=Homo sapiens OX=9606 GN=C9orf88 PE=3 SV=1 |
| 293 | 3 | 1 | 1 | 1 | [Master Protein] Epididymis secretory sperm binding protein OS=Homo sapiens OX=9606 PE=2 SV=1 |
| 409 | 4 | 1 | 1 | 1 | [Master Protein] NSFL1 cofactor p47 OS=Homo sapiens OX=9606 GN=NSFL1C PE=1 SV=1 |
| 322 | 1 | 1 | 1 | 1 | [Master Protein] Docking protein 3 OS=Homo sapiens OX=9606 GN=DOK3 PE=4 SV=1 |
| 364 | 5 | 1 | 1 | 1 | [Master Protein] Cell cycle control protein OS=Homo sapiens OX=9606 GN=TMEM30A PE=1 SV=1 |
| 363 | 3 | 1 | 1 | 1 | [Master Protein] S-(hydroxymethyl)glutathione dehydrogenase OS=Homo sapiens OX=9606 PE=2 SV=1 |
| 362 | 3 | 1 | 1 | 1 | [Master Protein] Lysosomal alpha-glucosidase OS=Homo sapiens OX=9606 GN=GAA PE=1 SV=4 |
| 361 | 6 | 1 | 1 | 1 | [Master Protein] cDNA FLJ55002, highly similar to Alpha-centractin OS=Homo sapiens OX=9606 PE=2 SV=1 |
| 360 | 1 | 1 | 1 | 1 | [Master Protein] Protein FAM3D OS=Homo sapiens OX=9606 GN=FAM3D PE=1 SV=1 |
| 324 | 5 | 1 | 1 | 1 | [Master Protein] DNA damage-binding protein 1 OS=Homo sapiens OX=9606 GN=DDB1 PE=1 SV=1 |
| 358 | 4 | 1 | 1 | 1 | [Master Protein] Polypeptide N-acetylgalactosaminyltransferase 2 OS=Homo sapiens OX=9606 GN=GALNT2 PE=1 SV=1 |
| 328 | 6 | 1 | 1 | 1 | [Master Protein] Reticulophagy regulator 3 OS=Homo sapiens OX=9606 GN=RETREG3 PE=1 SV=1 |
| 329 | 10 | 1 | 1 | 1 | [Master Protein] Glycoprotein IIIb OS=Homo sapiens OX=9606 GN=CD36 PE=2 SV=1 |
| 354 | 5 | 1 | 1 | 1 | [Master Protein] Lipolysis-stimulated lipoprotein receptor OS=Homo sapiens OX=9606 GN=LSR PE=1 SV=4 |
| 366 | 6 | 1 | 1 | 1 | [Master Protein] Nicastrin OS=Homo sapiens OX=9606 GN=NCSTN PE=1 SV=2 |
| 352 | 13 | 1 | 1 | 1 | [Master Protein] Receptor protein-tyrosine kinase OS=Homo sapiens OX=9606 PE=2 SV=1 |
| 349 | 2 | 1 | 1 | 1 | [Master Protein] Serine palmitoyltransferase 2 (Fragment) OS=Homo sapiens OX=9606 GN=SPTLC2 PE=1 SV=1 |
| 348 | 6 | 1 | 1 | 1 | [Master Protein] Acetyl-CoA acetyltransferase, mitochondrial OS=Homo sapiens OX=9606 GN=ACAT1 PE=1 SV=1 |
| 331 | 2 | 1 | 1 | 1 | [Master Protein] Vitronectin OS=Homo sapiens OX=9606 GN=VTN PE=4 SV=1 |
| 335 | 15 | 1 | 1 | 1 | [Master Protein] Complement decay-accelerating factor (Fragment) OS=Homo sapiens OX=9606 GN=CD55 PE=1 SV=1 |
| 345 | 4 | 1 | 1 | 1 | [Master Protein] Phospholipid scramblase (Fragment) OS=Homo sapiens OX=9606 GN=PLSCR1 PE=1 SV=1 |
| 344 | 10 | 1 | 1 | 1 | [Master Protein] Y-box-binding protein 3 OS=Homo sapiens OX=9606 GN=YBX3 PE=1 SV=4 |
| 343 | 3 | 1 | 1 | 1 | [Master Protein] Glutathione hydrolase 5 proenzyme OS=Homo sapiens OX=9606 GN=GGT5 PE=1 SV=2 |
| 342 | 1 | 1 | 1 | 1 | [Master Protein] Transmembrane protein 238 OS=Homo sapiens OX=9606 GN=TMEM238 PE=1 SV=1 |
| 341 | 5 | 1 | 1 | 1 | [Master Protein] Tax1-binding protein 2 isoform 2 OS=Homo sapiens OX=9606 GN=TAX1BP2 PE=2 SV=1 |
| 340 | 9 | 1 | 1 | 1 | [Master Protein] Ubiquitin thioesterase OS=Homo sapiens OX=9606 GN=OTUB1 PE=1 SV=2 |
| 336 | 4 | 1 | 1 | 1 | [Master Protein] ATP synthase subunit gamma (Fragment) OS=Homo sapiens OX=9606 PE=2 SV=1 |
| 350 | 3 | 1 | 1 | 1 | [Master Protein] H.sapiens ras-related Hrab2 protein OS=Homo sapiens OX=9606 PE=2 SV=1 |
| 410 | 4 | 1 | 1 | 1 | [Master Protein] Fructose-bisphosphatase OS=Homo sapiens OX=9606 PE=2 SV=1 |
| 368 | 4 | 1 | 1 | 1 | [Master Protein] Sortilin OS=Homo sapiens OX=9606 GN=SORT1 PE=1 SV=3 |
| 371 | 8 | 1 | 1 | 1 | [Master Protein] RNA-binding protein 47 OS=Homo sapiens OX=9606 GN=RBM47 PE=1 SV=2 |
| 407 | 1 | 1 | 1 | 1 | [Master Protein] Bis(5'-adenosyl)-triphosphatase ENPP4 OS=Homo sapiens OX=9606 GN=ENPP4 PE=1 SV=3 |
| 406 | 2 | 1 | 1 | 1 | [Master Protein] Acyl-protein thioesterase 2 OS=Homo sapiens OX=9606 GN=LYPLA2 PE=1 SV=1 |
| 295 | 3 | 1 | 1 | 1 | [Master Protein] Protein LYRIC (Fragment) OS=Homo sapiens OX=9606 GN=MTDH PE=1 SV=1 |
| 403 | 2 | 1 | 1 | 1 | [Master Protein] Tetraspanin (Fragment) OS=Homo sapiens OX=9606 PE=2 SV=1 |
| 400 | 3 | 1 | 1 | 1 | [Master Protein] UDP-Gal:betaGlcNAc beta 1,4-galactosyltransferase, polypeptide 4 OS=Homo sapiens OX=9606 GN=B4GALT4 PE=2 SV=1 |
| 299 | 2 | 1 | 1 | 1 | [Master Protein] T-complex protein 1 subunit delta OS=Homo sapiens OX=9606 GN=CCT4 PE=1 SV=4 |
| 397 | 5 | 1 | 1 | 1 | [Master Protein] Sorting nexin-27 OS=Homo sapiens OX=9606 GN=SNX27 PE=1 SV=1 |
| 395 | 2 | 1 | 1 | 1 | [Master Protein] cDNA FLJ53760, highly similar to Syntaxin-7 OS=Homo sapiens OX=9606 PE=2 SV=1 |
| 394 | 6 | 1 | 1 | 1 | [Master Protein] Galectin-3-binding protein OS=Homo sapiens OX=9606 PE=2 SV=1 |
| 392 | 4 | 1 | 1 | 1 | [Master Protein] Plexin-B2 (Fragment) OS=Homo sapiens OX=9606 GN=PLXNB2 PE=1 SV=1 |
| 391 | 2 | 1 | 1 | 1 | [Master Protein] Fatty acid synthase OS=Homo sapiens OX=9606 GN=FASN PE=1 SV=3 |
| 318 | 2 | 1 | 1 | 1 | [Master Protein] Sialic acid synthase OS=Homo sapiens OX=9606 GN=NANS PE=1 SV=2 |
| 390 | 2 | 1 | 1 | 1 | [Master Protein] Bone marrow stromal antigen 2 OS=Homo sapiens OX=9606 GN=BST2 PE=1 SV=1 |
| 302 | 6 | 1 | 1 | 1 | [Master Protein] Abl interactor 1 OS=Homo sapiens OX=9606 GN=ABI1 PE=1 SV=4 |
| 305 | 8 | 1 | 1 | 1 | [Master Protein] Polypeptide N-acetylgalactosaminyltransferase 3 OS=Homo sapiens OX=9606 GN=GALNT3 PE=1 SV=2 |
| 307 | 11 | 1 | 1 | 1 | [Master Protein] cDNA FLJ50442, highly similar to T-complex protein 1 subunit epsilon OS=Homo sapiens OX=9606 PE=2 SV=1 |
| 382 | 1 | 1 | 1 | 1 | [Master Protein] Saccharopine dehydrogenase-like oxidoreductase OS=Homo sapiens OX=9606 GN=SCCPDH PE=1 SV=1 |
| 381 | 18 | 1 | 1 | 1 | [Master Protein] cDNA FLJ52360, highly similar to Heat-shock protein 105 kDa OS=Homo sapiens OX=9606 PE=2 SV=1 |
| 308 | 9 | 1 | 1 | 1 | [Master Protein] Tetraspanin (Fragment) OS=Homo sapiens OX=9606 GN=CD81 PE=1 SV=1 |
| 309 | 5 | 1 | 1 | 1 | [Master Protein] Calcium-transporting ATPase OS=Homo sapiens OX=9606 GN=DKFZp686M088 PE=2 SV=1 |
| 310 | 13 | 1 | 1 | 1 | [Master Protein] Tryptase alpha/beta 1 OS=Homo sapiens OX=9606 GN=TPSAB1 PE=2 SV=1 |
| 377 | 3 | 1 | 1 | 1 | [Master Protein] Adhesion G protein-coupled receptor E5 OS=Homo sapiens OX=9606 GN=ADGRE5 PE=1 SV=4 |
| 312 | 2 | 1 | 1 | 1 | [Master Protein] Adipocyte plasma membrane-associated protein (Fragment) OS=Homo sapiens OX=9606 GN=APMAP PE=1 SV=1 |
| 313 | 9 | 1 | 1 | 1 | [Master Protein] Syntaxin-16 OS=Homo sapiens OX=9606 GN=STX16 PE=1 SV=1 |
| 301 | 7 | 1 | 1 | 1 | [Master Protein] Tetraspanin-31 OS=Homo sapiens OX=9606 GN=TSPAN31 PE=3 SV=1 |
| 163 | 4 | 1 | 1 | 1 | [Master Protein] Proteasome subunit alpha type (Fragment) OS=Homo sapiens OX=9606 GN=PSMA7 PE=2 SV=1 |
| 251 | 4 | 1 | 1 | 1 | [Master Protein] Nicotinate phosphoribosyltransferase OS=Homo sapiens OX=9606 GN=NAPRT PE=1 SV=2 |
| 484 | 5 | 1 | 1 | 1 | [Master Protein] Ras-related protein Rap-2 (Fragment) OS=Homo sapiens OX=9606 GN=DKFZp547A0616 PE=2 SV=1 |
| 189 | 2 | 1 | 1 | 1 | [Master Protein] cDNA FLJ57240, highly similar to Mitochondrial proteins import receptor OS=Homo sapiens OX=9606 PE=2 SV=1 |
| 591 | 1 | 1 | 1 | 1 | [Master Protein] IG c1409_heavy_IGHV4-39_IGHD3-3_IGHJ4 (Fragment) OS=Homo sapiens OX=9606 PE=2 SV=1 |
| 590 | 2 | 1 | 1 | 1 | [Master Protein] 3'-phosphate/5'-hydroxy nucleic acid ligase OS=Homo sapiens OX=9606 PE=2 SV=1 |
| 191 | 1 | 1 | 1 | 1 | [Master Protein] Protein disulfide-isomerase A5 OS=Homo sapiens OX=9606 GN=PDIA5 PE=1 SV=1 |
| 587 | 1 | 1 | 1 | 1 | [Master Protein] Coatomer subunit gamma-1 OS=Homo sapiens OX=9606 GN=COPG1 PE=1 SV=1 |
| 586 | 3 | 1 | 1 | 1 | [Master Protein] 60S ribosomal protein L32 (Fragment) OS=Homo sapiens OX=9606 GN=RPL32 PE=1 SV=1 |
| 192 | 2 | 1 | 1 | 1 | [Master Protein] Epiplakin OS=Homo sapiens OX=9606 GN=EPPK1 PE=1 SV=3 |
| 193 | 8 | 1 | 1 | 1 | [Master Protein] 60S ribosomal protein L22 (Fragment) OS=Homo sapiens OX=9606 GN=RPL22 PE=1 SV=1 |
| 583 | 4 | 1 | 1 | 1 | [Master Protein] Pleckstrin homology domain-containing family A member 6 (Fragment) OS=Homo sapiens OX=9606 GN=PLEKHA6 PE=1 SV=1 |
| 582 | 2 | 1 | 1 | 1 | [Master Protein] Metal transporter CNNM2 OS=Homo sapiens OX=9606 GN=CNNM2 PE=1 SV=2 |
| 580 | 1 | 1 | 1 | 1 | [Master Protein] Oxidoreductase HTATIP2 OS=Homo sapiens OX=9606 GN=HTATIP2 PE=1 SV=2 |
| 187 | 8 | 1 | 1 | 1 | [Master Protein] CD59 blood group antigen (Fragment) OS=Homo sapiens OX=9606 PE=4 SV=1 |
| 577 | 1 | 1 | 1 | 1 | [Master Protein] Inhibitor of nuclear factor kappa-B kinase-interacting protein OS=Homo sapiens OX=9606 GN=IKBIP PE=1 SV=1 |
| 198 | 6 | 1 | 1 | 1 | [Master Protein] Cathepsin B OS=Homo sapiens OX=9606 PE=2 SV=1 |
| 572 | 2 | 1 | 1 | 1 | [Master Protein] Splicing factor 3B subunit 1 OS=Homo sapiens OX=9606 GN=SF3B1 PE=1 SV=3 |
| 201 | 4 | 1 | 1 | 1 | [Master Protein] cDNA FLJ11224 fis, clone PLACE1008273, moderately similar to COATOMER GAMMA SUBUNIT OS=Homo sapiens OX=9606 PE=2 SV=1 |
| 568 | 6 | 1 | 1 | 1 | [Master Protein] Atlastin-2 (Fragment) OS=Homo sapiens OX=9606 GN=ATL2 PE=1 SV=1 |
| 566 | 2 | 1 | 1 | 1 | [Master Protein] Secretory carrier-associated membrane protein 3 OS=Homo sapiens OX=9606 GN=SCAMP3 PE=1 SV=3 |
| 202 | 6 | 1 | 1 | 1 | [Master Protein] Nucleolin (Fragment) OS=Homo sapiens OX=9606 GN=NCL PE=1 SV=8 |
| 564 | 4 | 1 | 1 | 1 | [Master Protein] Dynamin 2 isoform 4 variant (Fragment) OS=Homo sapiens OX=9606 PE=2 SV=1 |
| 205 | 5 | 1 | 1 | 1 | [Master Protein] cDNA FLJ61181, highly similar to Homo sapiens hydroxysteroid (17-beta) dehydrogenase 12 (HSD17B12), mRNA OS=Homo sapiens OX=9606 PE=2 SV=1 |
| 561 | 1 | 1 | 1 | 1 | [Master Protein] ATPase, H+ transporting, lysosomal 31kDa, V1 subunit E isoform 1 OS=Homo sapiens OX=9606 GN=ATP6V1E1 PE=2 SV=1 |
| 206 | 15 | 1 | 1 | 1 | [Master Protein] Band 4.1-like protein 3 OS=Homo sapiens OX=9606 GN=EPB41L3 PE=1 SV=1 |
| 559 | 1 | 1 | 1 | 1 | [Master Protein] Junctional adhesion molecule C OS=Homo sapiens OX=9606 GN=JAM3 PE=1 SV=1 |
| 576 | 3 | 1 | 1 | 1 | [Master Protein] Monocyte differentiation antigen CD14 OS=Homo sapiens OX=9606 PE=2 SV=1 |
| 207 | 13 | 1 | 1 | 1 | [Master Protein] 2-oxoglutarate dehydrogenase, mitochondrial OS=Homo sapiens OX=9606 GN=OGDH PE=1 SV=1 |
| 186 | 12 | 1 | 1 | 1 | [Master Protein] cDNA FLJ44468 fis, clone UTERU2026025, moderately similar to SPLICING FACTOR, ARGININE/SERINE-RICH 2 OS=Homo sapiens OX=9606 PE=2 SV=1 |
| 596 | 3 | 1 | 1 | 1 | [Master Protein] Chemokine-like receptor 1 (Fragment) OS=Homo sapiens OX=9606 GN=CMKLR1 PE=3 SV=1 |
| 165 | 6 | 1 | 1 | 1 | [Master Protein] cDNA FLJ53692, moderately similar to Granulins OS=Homo sapiens OX=9606 PE=2 SV=1 |
| 170 | 4 | 1 | 1 | 1 | [Master Protein] Dynactin subunit 1 OS=Homo sapiens OX=9606 GN=DCTN1 PE=1 SV=3 |
| 626 | 2 | 1 | 1 | 1 | [Master Protein] Coatomer subunit beta (Fragment) OS=Homo sapiens OX=9606 GN=COPB1 PE=1 SV=8 |
| 171 | 5 | 1 | 1 | 1 | [Master Protein] Long-chain fatty acid transport protein 1 OS=Homo sapiens OX=9606 GN=SLC27A1 PE=1 SV=1 |
| 623 | 1 | 1 | 1 | 1 | [Master Protein] Down syndrome critical region protein 9 OS=Homo sapiens OX=9606 GN=DSCR9 PE=2 SV=1 |
| 622 | 1 | 1 | 1 | 1 | [Master Protein] Kallikrein 13 splice variant 7 OS=Homo sapiens OX=9606 GN=KLK13 PE=2 SV=1 |
| 620 | 1 | 1 | 1 | 1 | [Master Protein] Cytochrome b reductase 1 OS=Homo sapiens OX=9606 GN=CYBRD1 PE=1 SV=1 |
| 173 | 4 | 1 | 1 | 1 | [Master Protein] Fibrinogen beta chain OS=Homo sapiens OX=9606 GN=FGB PE=4 SV=1 |
| 618 | 4 | 1 | 1 | 1 | [Master Protein] ATP-dependent 6-phosphofructokinase, platelet type OS=Homo sapiens OX=9606 GN=PFKP PE=1 SV=2 |
| 174 | 2 | 1 | 1 | 1 | [Master Protein] Transmembrane protein 179B OS=Homo sapiens OX=9606 GN=TMEM179B PE=1 SV=1 |
| 175 | 5 | 1 | 1 | 1 | [Master Protein] GO2-q chimeric G-protein OS=Homo sapiens OX=9606 PE=2 SV=1 |
| 595 | 6 | 1 | 1 | 1 | [Master Protein] Glutathione synthetase OS=Homo sapiens OX=9606 PE=2 SV=1 |
| 615 | 4 | 1 | 1 | 1 | [Master Protein] Cullin 1 protein (Fragment) OS=Homo sapiens OX=9606 GN=CUL1 PE=2 SV=1 |
| 610 | 2 | 1 | 1 | 1 | [Master Protein] Peptidyl-prolyl cis-trans isomerase OS=Homo sapiens OX=9606 GN=HEL-S-39 PE=2 SV=1 |
| 609 | 3 | 1 | 1 | 1 | [Master Protein] GlcNAc kinase OS=Homo sapiens OX=9606 PE=2 SV=1 |
| 179 | 3 | 1 | 1 | 1 | [Master Protein] Partitioning defective 6 homolog gamma OS=Homo sapiens OX=9606 GN=PARD6G PE=1 SV=1 |
| 180 | 3 | 1 | 1 | 1 | [Master Protein] 6-phosphogluconolactonase (Fragment) OS=Homo sapiens OX=9606 GN=PGLS PE=1 SV=1 |
| 605 | 3 | 1 | 1 | 1 | [Master Protein] Inositol 1,4,5-trisphosphate receptor type 3 OS=Homo sapiens OX=9606 GN=ITPR3 PE=1 SV=2 |
| 181 | 3 | 1 | 1 | 1 | [Master Protein] Aspartate aminotransferase OS=Homo sapiens OX=9606 PE=2 SV=1 |
| 603 | 1 | 1 | 1 | 1 | [Master Protein] Keratin, type II cytoskeletal 80 OS=Homo sapiens OX=9606 GN=KRT80 PE=1 SV=2 |
| 602 | 2 | 1 | 1 | 1 | [Master Protein] Lymphocyte antigen 75 OS=Homo sapiens OX=9606 GN=LY75 PE=1 SV=3 |
| 599 | 2 | 1 | 1 | 1 | [Master Protein] 3'-phosphoadenosine-5'-phosphosulfate synthase OS=Homo sapiens OX=9606 PE=2 SV=1 |
| 184 | 3 | 1 | 1 | 1 | [Master Protein] FLJ00144 protein (Fragment) OS=Homo sapiens OX=9606 GN=FLJ00144 PE=2 SV=1 |
| 185 | 4 | 1 | 1 | 1 | [Master Protein] 60S ribosomal protein L12 OS=Homo sapiens OX=9606 GN=RPL12 PE=1 SV=1 |
| 613 | 2 | 1 | 1 | 1 | [Master Protein] Protein mono-ADP-ribosyltransferase PARP4 OS=Homo sapiens OX=9606 GN=PARP4 PE=1 SV=3 |
| 208 | 1 | 1 | 1 | 1 | [Master Protein] 5'-AMP-activated protein kinase catalytic subunit alpha-1 OS=Homo sapiens OX=9606 GN=PRKAA1 PE=1 SV=4 |
| 209 | 5 | 1 | 1 | 1 | [Master Protein] Apoptotic chromatin condensation inducer in the nucleus OS=Homo sapiens OX=9606 GN=ACIN1 PE=1 SV=2 |
| 551 | 1 | 1 | 1 | 1 | [Master Protein] DC30 OS=Homo sapiens OX=9606 PE=2 SV=1 |
| 516 | 4 | 1 | 1 | 1 | [Master Protein] Vacuolar protein sorting-associated protein 26A OS=Homo sapiens OX=9606 GN=VPS26A PE=1 SV=1 |
| 230 | 1 | 1 | 1 | 1 | [Master Protein] MHC class I protein (Fragment) OS=Homo sapiens OX=9606 GN=HLA-B PE=3 SV=1 |
| 514 | 3 | 1 | 1 | 1 | [Master Protein] Unconventional myosin-Ig OS=Homo sapiens OX=9606 GN=MYO1G PE=1 SV=1 |
| 513 | 1 | 1 | 1 | 1 | [Master Protein] Mitotic interactor and substrate of PLK1 OS=Homo sapiens OX=9606 GN=MISP PE=1 SV=1 |
| 231 | 5 | 1 | 1 | 1 | [Master Protein] Thymidine phosphorylase (Fragment) OS=Homo sapiens OX=9606 GN=TYMP PE=1 SV=1 |
| 509 | 4 | 1 | 1 | 1 | [Master Protein] Protein GOLM2 OS=Homo sapiens OX=9606 GN=GOLM2 PE=1 SV=2 |
| 232 | 4 | 1 | 1 | 1 | [Master Protein] Protein ABHD14B OS=Homo sapiens OX=9606 GN=ABHD14B PE=1 SV=1 |
| 233 | 3 | 1 | 1 | 1 | [Master Protein] Immunoglobulin mu heavy chain OS=Homo sapiens OX=9606 PE=1 SV=2 |
| 506 | 1 | 1 | 1 | 1 | [Master Protein] General vesicular transport factor p115 OS=Homo sapiens OX=9606 GN=USO1 PE=1 SV=2 |
| 505 | 2 | 1 | 1 | 1 | [Master Protein] Mannose-1-phosphate guanyltransferase beta OS=Homo sapiens OX=9606 GN=GMPPB PE=1 SV=2 |
| 501 | 2 | 1 | 1 | 1 | [Master Protein] Elongation factor Tu OS=Homo sapiens OX=9606 GN=TUFM PE=1 SV=1 |
| 517 | 3 | 1 | 1 | 1 | [Master Protein] Wiskott-Aldrich syndrome protein family member OS=Homo sapiens OX=9606 PE=2 SV=1 |
| 500 | 4 | 1 | 1 | 1 | [Master Protein] Testicular tissue protein Li 70 OS=Homo sapiens OX=9606 PE=2 SV=1 |
| 498 | 5 | 1 | 1 | 1 | [Master Protein] Macrophage-capping protein (Fragment) OS=Homo sapiens OX=9606 GN=CAPG PE=1 SV=1 |
| 240 | 3 | 1 | 1 | 1 | [Master Protein] Tumor differentially expressed protein 1 variant (Fragment) OS=Homo sapiens OX=9606 PE=2 SV=1 |
| 241 | 8 | 1 | 1 | 1 | [Master Protein] Serine/threonine-protein phosphatase OS=Homo sapiens OX=9606 GN=PPP2CA PE=2 SV=1 |
| 242 | 5 | 1 | 1 | 1 | [Master Protein] Protein NDRG2 OS=Homo sapiens OX=9606 PE=2 SV=1 |
| 494 | 1 | 1 | 1 | 1 | [Master Protein] Cell cycle control protein 50B OS=Homo sapiens OX=9606 GN=TMEM30B PE=1 SV=1 |
| 493 | 4 | 1 | 1 | 1 | [Master Protein] Villin-like variant (Fragment) OS=Homo sapiens OX=9606 PE=2 SV=1 |
| 491 | 5 | 1 | 1 | 1 | [Master Protein] MOB kinase activator 1B (Fragment) OS=Homo sapiens OX=9606 GN=MOB1B PE=1 SV=1 |
| 246 | 5 | 1 | 1 | 1 | [Master Protein] Proteasome subunit beta type-8 OS=Homo sapiens OX=9606 GN=PSMB8 PE=1 SV=1 |
| 489 | 8 | 1 | 1 | 1 | [Master Protein] Acetyltransferase component of pyruvate dehydrogenase complex OS=Homo sapiens OX=9606 PE=2 SV=1 |
| 487 | 2 | 1 | 1 | 1 | [Master Protein] cDNA, FLJ93804, highly similar to Homo sapiens gp25L2 protein (HSGP25L2G), mRNA OS=Homo sapiens OX=9606 PE=2 SV=1 |
| 250 | 5 | 1 | 1 | 1 | [Master Protein] Solute carrier family 30 member 9 OS=Homo sapiens OX=9606 PE=2 SV=1 |
| 237 | 7 | 1 | 1 | 1 | [Master Protein] Endothelin-converting enzyme 1 OS=Homo sapiens OX=9606 GN=ECE1 PE=1 SV=2 |
| 483 | 1 | 1 | 1 | 1 | [Master Protein] Serum amyloid P-component OS=Homo sapiens OX=9606 GN=APCS PE=1 SV=2 |
| 227 | 4 | 1 | 1 | 1 | [Master Protein] Sulfhydryl oxidase OS=Homo sapiens OX=9606 GN=QSCN6 PE=2 SV=1 |
| 217 | 3 | 1 | 1 | 1 | [Master Protein] 1,4-beta-N-acetylmuramidase C OS=Homo sapiens OX=9606 GN=LYZ PE=1 SV=1 |
| 550 | 4 | 1 | 1 | 1 | [Master Protein] Plasma membrane citrate carrier OS=Homo sapiens OX=9606 GN=SLC25A1 PE=2 SV=1 |
| 549 | 2 | 1 | 1 | 1 | [Master Protein] Complement C3 OS=Homo sapiens OX=9606 GN=C3 PE=1 SV=2 |
| 210 | 2 | 1 | 1 | 1 | [Master Protein] Synaptogyrin OS=Homo sapiens OX=9606 GN=SYNGR2 PE=3 SV=1 |
| 547 | 3 | 1 | 1 | 1 | [Master Protein] Calcium-activated neutral proteinase 2 OS=Homo sapiens OX=9606 PE=2 SV=1 |
| 211 | 8 | 1 | 1 | 1 | [Master Protein] HP protein OS=Homo sapiens OX=9606 GN=HP PE=2 SV=1 |
| 544 | 10 | 1 | 1 | 1 | [Master Protein] Histone H1.5 OS=Homo sapiens OX=9606 GN=H1-5 PE=1 SV=3 |
| 543 | 1 | 1 | 1 | 1 | [Master Protein] Multi-functional protein MFP OS=Homo sapiens OX=9606 PE=2 SV=1 |
| 542 | 1 | 1 | 1 | 1 | [Master Protein] Delta-1-pyrroline-5-carboxylate synthase OS=Homo sapiens OX=9606 GN=ALDH18A1 PE=1 SV=2 |
| 212 | 10 | 1 | 1 | 1 | [Master Protein] Tripeptidyl aminopeptidase OS=Homo sapiens OX=9606 PE=2 SV=1 |
| 540 | 4 | 1 | 1 | 1 | [Master Protein] Bromodomain adjacent to zinc finger domain protein 1A OS=Homo sapiens OX=9606 GN=BAZ1A PE=1 SV=2 |
| 215 | 6 | 1 | 1 | 1 | [Master Protein] RuvB-like 2 OS=Homo sapiens OX=9606 GN=RUVBL2 PE=1 SV=3 |
| 537 | 2 | 1 | 1 | 1 | [Master Protein] IG c1739_heavy_IGHV3-21_IGHD3-9_IGHJ5 (Fragment) OS=Homo sapiens OX=9606 PE=2 SV=1 |
| 429 | 6 | 1 | 1 | 1 | [Master Protein] cDNA FLJ56281, highly similar to 6-phosphofructokinase, muscle type OS=Homo sapiens OX=9606 PE=2 SV=1 |
| 534 | 6 | 1 | 1 | 1 | [Master Protein] cDNA FLJ51066, highly similar to Ras-related protein Rab-6A OS=Homo sapiens OX=9606 PE=2 SV=1 |
| 218 | 8 | 1 | 1 | 1 | [Master Protein] Integrin beta OS=Homo sapiens OX=9606 GN=ITGB2 PE=1 SV=1 |
| 532 | 1 | 1 | 1 | 1 | [Master Protein] Protein S100-A8 OS=Homo sapiens OX=9606 GN=S100A8 PE=1 SV=1 |
| 531 | 2 | 1 | 1 | 1 | [Master Protein] Transient receptor potential cation channel subfamily M member 4 OS=Homo sapiens OX=9606 GN=TRPM4 PE=1 SV=1 |
| 529 | 4 | 1 | 1 | 1 | [Master Protein] Dihydrolipoyl dehydrogenase OS=Homo sapiens OX=9606 PE=2 SV=1 |
| 221 | 5 | 1 | 1 | 1 | [Master Protein] 60S ribosomal protein L9 OS=Homo sapiens OX=9606 GN=RPL9 PE=1 SV=1 |
| 526 | 5 | 1 | 1 | 1 | [Master Protein] cDNA FLJ77899, highly similar to Homo sapiens caspase 1, apoptosis-related cysteine protease(interleukin 1, beta, convertase) (CASP1), transcript variantalpha, mRNA OS=Homo sapiens OX=9606 PE=2 SV=1 |
| 525 | 5 | 1 | 1 | 1 | [Master Protein] Terpene cyclase/mutase family member OS=Homo sapiens OX=9606 PE=2 SV=1 |
| 523 | 7 | 1 | 1 | 1 | [Master Protein] Myeloid-associated differentiation marker (Fragment) OS=Homo sapiens OX=9606 GN=MYADM PE=1 SV=8 |
| 522 | 4 | 1 | 1 | 1 | [Master Protein] cDNA FLJ52867, highly similar to Rho guanine nucleotide exchange factor 16 OS=Homo sapiens OX=9606 PE=2 SV=1 |
| 521 | 4 | 1 | 1 | 1 | [Master Protein] Sorting nexin-2 (Fragment) OS=Homo sapiens OX=9606 PE=2 SV=1 |
| 226 | 3 | 1 | 1 | 1 | [Master Protein] Serum paraoxonase/arylesterase 1 OS=Homo sapiens OX=9606 GN=PON1 PE=1 SV=3 |
| 519 | 6 | 1 | 1 | 1 | [Master Protein] Vesicle transport-related protein isoform a variant (Fragment) OS=Homo sapiens OX=9606 PE=2 SV=1 |
| 61 | 468 | 0 | 3 | 3 | [Master Protein] MHC class I antigen OS=Homo sapiens OX=9606 GN=HLA-C PE=3 SV=1 |
